# Reappraisal of the extinct seal “*Phoca*” *vitulinoides* from the Neogene of the North Sea Basin, with bearing on its geological age, phylogenetic affinities, and locomotion

**DOI:** 10.7717/peerj.3316

**Published:** 2017-05-16

**Authors:** Leonard Dewaele, Eli Amson, Olivier Lambert, Stephen Louwye

**Affiliations:** 1 Department of Geology, Ghent University, Ghent, Belgium; 2 O.D. Earth and History of Life, Royal Belgian Institute of Natural Sciences, Brussels, Belgium; 3 Arbeitsgruppe Morphologie und Formengeschichte, Humboldt Universität Berlin, Berlin, Germany; 4 Paläontologisches Institut und Museum, University of Zürich, Zürich, Switzerland

**Keywords:** Phocidae, Neogene, North Sea Basin, Belgium, Redescription, Taxonomy, Locomotion

## Abstract

**Background:**

Discovered on the southern margin of the North Sea Basin, “*Phoca*” *vitulinoides* represents one of the best-known extinct species of Phocidae. However, little attention has been given to the species ever since its original 19th century description. Newly discovered material, including the most complete specimen of fossil Phocidae from the North Sea Basin, prompted the redescription of the species. Also, the type material of “*Phoca*” *vitulinoides* is lost.

**Methods:**

“*Phoca*” *vitulinoides* is redescribed. Its phylogenetic position among Phocinae is assessed through phylogenetic analysis. Dinoflagellate cyst biostratigraphy is used to determine and reassess the geological age of the species. Myological descriptions of extant taxa are used to infer muscle attachments, and basic comparative anatomy of the gross morphology and biomechanics are applied to reconstruct locomotion.

**Results:**

Detailed redescription of “*Phoca*” *vitulinoides* indicates relatively little affinities with the genus *Phoca*, but rather asks for the establishment of a new genus: *Nanophoca* gen. nov. Hence, “*Phoca*” *vitulinoides* is recombined into *Nanophoca vitulinoides*. This reassignment is confirmed by the phylogenetic analysis, grouping the genus *Nanophoca* and other extinct phocine taxa as stem phocines. Biostratigraphy and lithostratigraphy expand the known stratigraphic range of *N. vitulinoides* from the late Langhian to the late Serravallian. The osteological anatomy of *N. vitulinoides* indicates a relatively strong development of muscles used for fore flipper propulsion and increased flexibility for the hind flipper.

**Discussion:**

The extended stratigraphic range of *N. vitulinoides* into the middle Miocene confirms relatively early diversification of Phocinae in the North Atlantic. Morphological features on the fore- and hindlimb of the species point toward an increased use of the fore flipper and greater flexibility of the hind flipper as compared to extant Phocinae, clearly indicating less derived locomotor strategies in this Miocene phocine species. Estimations of the overall body size indicate that *N. vitulinoides* is much smaller than *Pusa*, the smallest extant genus of Phocinae (and Phocidae), and than most extinct phocines.

## Introduction

The fossil record of Phocidae Gray, 1821 (Mammalia, Carnivora) is poorly known and largely consists of isolated and fragmentary material (Ray, 1976; Koretsky, 2001). Apart from a limited number of isolated localities (Tavani, 1941; Muizon & Bond, 1982; Walsh & Naish, 2002; Valenzuela-Toro et al., 2013), virtually all Neogene fossil material comes from five relatively phocid fossil-rich areas dispersed around the world: (1) the Miocene of the Paratethys region and the Mediterranean region (Koretsky, 2001), (2) the Miocene and Pliocene of the North American East Coast (True, 1906; Ray, 1976; Koretsky & Ray, 2008), (3) the Miocene and (presumably) Pliocene of the southern North Sea Basin, including both the Belgian Antwerp area and the Netherlands (Van Beneden, 1859, 1871, 1876, 1877; Koretsky & Peters, 2008; Koretsky, Ray & Peters, 2012; Koretsky, Peters & Rahmat, 2015), (4) the Miocene/Pliocene Pisco Formation of Peru (Muizon, 1981; Amson & Muizon, 2014; Valenzuela-Toro et al., 2016), and (5) the Miocene and Pliocene of Langebaanweg, South Africa (Hendey & Repenning, 1971; Muizon & Hendey, 1980; Govender, Chinsamy & Rogers Ackermann, 2012).

The family Phocidae is subdivided in two extant subfamilies: Monachinae Gray, 1869 and Phocinae Gray, 1821; and one extinct subfamily: Devinophocinae Koretsky & Holec, 2002. Devinophocinae only includes *Devinophoca claytoni* Koretsky & Holec, 2002 and *Devinophoca emryi* Koretsky & Rahmat, 2015, both from the Serravalian of Slovakia. The extant subfamilies Monachinae and Phocinae are easily discernable, as has been proven by numerous molecular and morphological phylogenetic analyses (Muizon, 1981; Berta & Wyss, 1994; Bininda-Emonds & Russell, 1996; Higdon et al., 2007; Arnason et al., 2006; Amson & Muizon, 2014). Generally, Monachinae tend to be larger than Phocinae (see Valenzuela-Toro et al., 2016). Despite the co-occurrence of both subfamilies in the Northern Hemisphere during the Neogene (Koretsky & Ray, 2008), they are currently biogeographically separated: Monachinae include the Antarctic seals, the subtropical monk seals (*Monachus* spp.), and the elephant seals (*Mirounga* spp.) along the eastern North Pacific and subantarctic waters, while Phocinae are restricted to the Northern temperate and Arctic coasts. Phocine and monachine ranges only overlap in the North Eastern Pacific, where the range of the harbor seal, *Phoca vitulina* Linaeus, 1758, overlaps with that of the northern elephant seal, *Mirounga angustirostris* Gill, 1866. A number of researchers have grouped the monachine *Mirounga* Gray, 1827 and the phocine hooded seal, *Cystophora cristata* (Erxleben, 1777) into a separate subfamily Cystophorinae Gray, 1869 (Chapskii, 1974; Koretsky & Rahmat, 2013) and some researchers have grouped the Antarctic seals into Lobodontinae Hay, 1930. However, the existence of Cystophorinae has been contradicted by molecular and morphological evidence (King, 1966; Higdon et al., 2007; Fulton & Strobeck, 2010) and members of Lobodontinae are generally considered to make a monachine tribe Lobodontini (Muizon, 1981; Amson & Muizon, 2014; Berta et al., 2015).

Apart from the monachines *Acrophoca longirostris* Muizon, 1981, *Hadrokirus martini* Amson & de Muizon, 2013, *Homiphoca capensis* (Hendey & Repenning, 1971), *Piscophoca pacifica* Muizon, 1981, and *Pliophoca etrusca* Tavani, 1941, in which the skeleton is almost completely known (Tavani, 1941; Hendey & Repenning, 1971; Muizon & Hendey, 1980; Muizon, 1981; Amson & Muizon, 2014; Berta et al., 2015), the overall fossil record of Phocidae predominantly consists of disarticulated cranial and postcranial elements (Van Beneden, 1877). Extinct phocines in particular are nearly exclusively known from isolated bones or sets of a few articulated bones (Koretsky, 2001; Koretsky, Peters & Rahmat, 2015). The species “*Phoca*” *vitulinoides* Van Beneden, 1871, from the Neogene of the southern margin of the North Sea Basin (Antwerp area, Belgium), is arguably one of the most completely known phocine seals (Van Beneden, 1877), apart from *Praepusa vindobonensis* (Toula, 1897), and maybe *Leptophoca proxima* (Van Beneden, 1877); these two species are known based on a series of postcranial remains (Van Beneden, 1877; Toula, 1897; Koretsky, 2001; Dewaele, Lambert & Louwye, 2017). The strong need for a redescription of “*Phoca*” *vitulinoides* has been stated on multiple occasions and it has been proposed that the generic attribution of “*Ph. vitulinoides*” is erroneous (Koretsky & Ray, 2008; Koretsky & Peters, 2008). Indeed, Van Beneden (1877) considered the species referable to the genus *Phoca* Linnaeus, 1758 on the basis of similarities with *Pusa hispida* (Schreber, 1775), at the time considered *Phoca hispida*. Even today, the phylogentic position of *Pusa* Scopoli, 1777 among Phocinae remains questionable, both based on morphological and molecular data (Bininda-Emonds & Russell, 1996; Higdon et al., 2007; Fulton & Strobeck, 2010). Therefore, a redescription of “*Phoca*” *vitulinoides* and an investigation of its phylogenetic affinities are required.

Because the phocid material at the IRSNB has not been reinvestigated for a long time, the proposed stratigraphic range of “*Phoca*” *vitulinoides* does not include more recently discovered specimens nor has the stratigraphic position of the known specimens been reassessed. Neither has it formally been shown that the syntype material of “*Phoca*” *vitulinoides*, presented by Van Beneden (1871), has been lost. The currently described stratigraphic time range for “*Ph*.” *vitulinoides* is far from satisfactory; all published specimens from the IRSNB had been assigned a “Scaldisian” age (Van Beneden, 1877), a confusing and disused term with little precise age determination (Laga & Louwye, 2006). Dinoflagellate cyst biostratigraphy of sediment preserved in cavities of several specimens provides the opportunity to reassess the geologic age and origin of these specimens.

**Figure 1:**
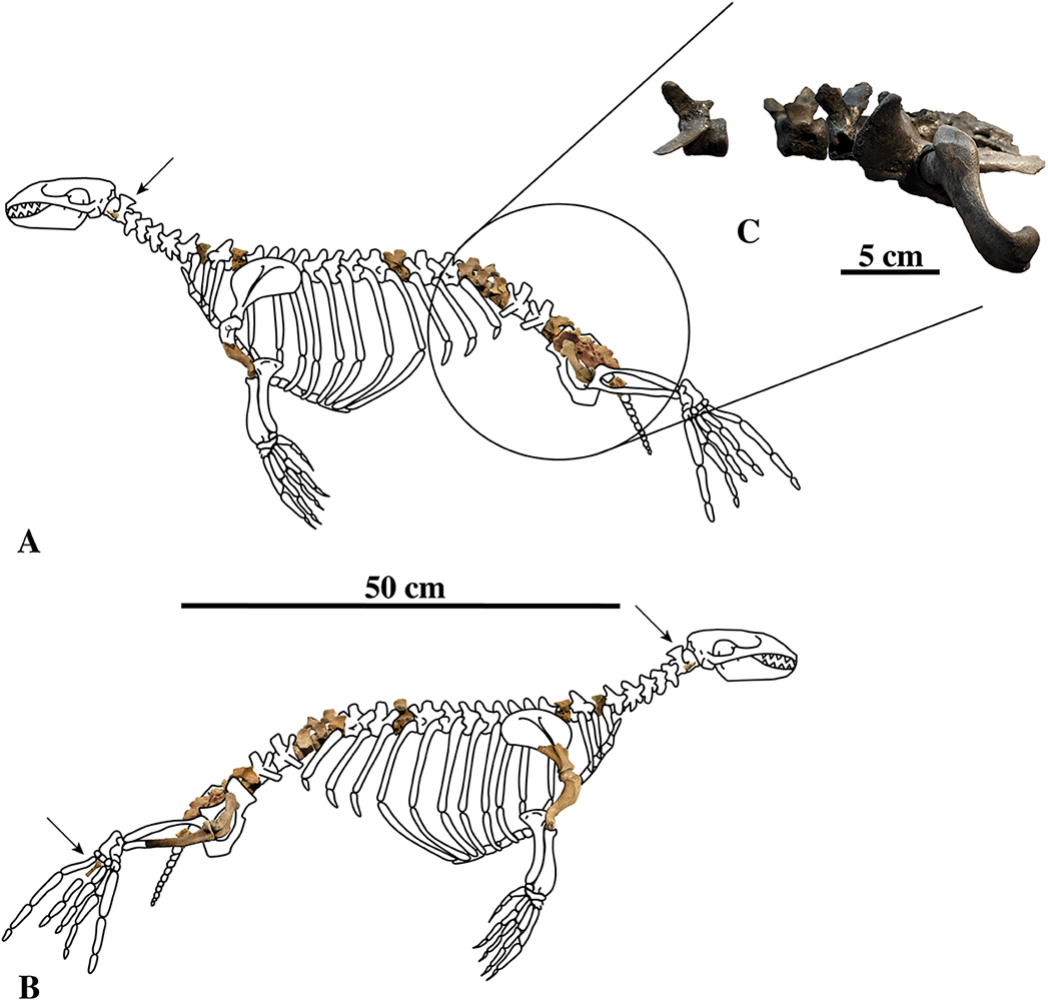
*Nanophoca vitulinoides* neotype and other articulated specimen. Left (A) and right (B) lateral views of a generalised and simplified phocine skeleton with the bones of the neotype specimen of Nanophoca vitulinoides (IRSNB M2276) shown. Black arrows indicate the smaller partial axis (IRSNB M2276i) and metatarsal (IRSNB M2276h). The second most complete specimen of *Nanophoca vitulinoides*, IRSNB 1059-M240 is shown in close-up (C).

Furthermore, the IRSNB recently acquired one partial postcranial skeleton of “*Ph*.” *vitulinoides* (IRSNB M2276a-q), which is the most complete phocid skeleton ever recorded from the North Sea Basin (Fig. 1). Similarly, access to the private collection of Paul and Gigase was provided for study. In agreement with the latter, selected specimens were transferred to the collection of the IRSNB (IRSNB M2269, IRSNB M2270, and IRSNB M2271). The access to new specimens of “*Ph*.” *vitulinoides* further spurred the redescription of the species and the reassessment of its stratigraphic range, phylogenetic position, and paleoecology.

## Historical Background

“*Phoca*” *vitulinoides* was one of the earliest extinct seals from the Antwerp area to be described by Van Beneden (1871). Although Van Beneden (1871, 1877) states that remains of “*Ph*.” *vitulinoides* were first mentioned in 1859 publication on extinct marine mammals from the city of Sint-Niklaas, we could not find any mention of fossils of “*Ph*.” *vitulinoides* in Van Beneden’s 1859 publication. In the 1871 description, a small set of poorly diagnostic, isolated bones was grouped together to establish the species; the original material consisted of a maxilla, an atlas, an ulna, a sacrum, two calcanea, and a phalanx, and illustrations were only provided for the atlas, ulna, sacrum, one of the calcanea (which proves to be an astragalus), and the phalanx (Van Beneden, 1871, p. 1). This original description of “*Ph*.” *vitulinoides* is short and little detailed, and no argument is provided explaining for example the referral of the isolated maxilla to the same species as the other bones. The etymology of the species epithet *vitulinoides* is based on the superficial similarities of the generally poorly diagnostic material with the extant harbor seal *Phoca vitulina* (Van Beneden, 1871).

“*Phoca*” *vitulinoides* is then only shortly mentioned in Van Beneden’s next publication (Van Beneden, 1876), and a more detailed description appears in his 1877 magnum opus on Phocidae from the Antwerp area (Van Beneden, 1877), including the attribution of more recently discovered material to the species. At the time, “*Phoca*” *vitulinoides* was considered the best-known extinct phocid from the Neogene of the southern margin of the North Sea, with 125 specimens in the collection of the IRSNB, ranging from fragmentary elements to seven articulated bones, representing almost the entire postcranial skeleton (Van Beneden, 1877).

Following the redescription of “*Phoca*” *vitulinoides* by Van Beneden (1877), the collection at the IRSNB expanded considerably during the 20th century. Also, private collectors acquired another considerable body of specimens. However, research on extinct seals largely neglected these collections and apart from Friant (1944), “*Ph*.” *vitulinoides* has only been mentioned in research focusing on other taxa (Koretsky & Peters, 2008) or in review studies (Kellogg, 1922; Koretsky & Ray, 2008). Friant (1944) considered the species when erecting the new species *Phocanella straeleni* Friant, 1944 from the “Scaldisian” of the third section of the fortification ring around Antwerp, saying it is a very specialized species (considering the femur), better adapted to an aquatic lifestyle than *Phoca vitulina*. King (1964) accepted the validity of *Phocanella straeleni*, but it was subsequently degraded to a nomen dubium by Koretsky & Ray (2008).

More recently, Koretsky & Ray (2008) briefly dealt with “*Phoca*” *vitulinoides* in their redescription of Pliocene North Atlantic seals. However, their research focused on species occurring along both the eastern and western margins of the North Atlantic realm. Hence, because “*Ph*.” *vitulinoides* is currently only known from the southern margin of the North Sea, they only noted that Van Beneden apparently lumped two species in “*Ph*.” *vitulinoides*. They interpreted the specimens (excluding the sacrum) in Van Beneden (1871) as representing a much larger species than the material presented in the subsequent paper (Van Beneden, 1877). When assigning a lectotype to “*Ph*.” *vitulinoides*, Koretsky & Ray (2008, p. 88) stated the following: “We concluded that under *Phoca vitulinoides* we have to admit the greater seal of Van Beneden (1871), but not the smaller seal, as described and illustrated later by Van Beneden (1877, pp. 72–74, atlas pl. 15). Although none of the specimens in Van Beneden’s original hypodigm is truly satisfactory, we choose the sacrum as the least unsatisfactory lectotype. We believe, however, that this bone is not diagnostic at the species level, and therefore regard *Phoca vitulinoides* as a nomen dubium.” A similar statement is repeated by Koretsky & Peters (2008).

## Materials and Methods

### Specimens studied

#### The IRSNB collection

This collection comprises (1) nearly all fossil seal specimens from the Antwerp area that have been illustrated or described in the past (Van Beneden, 1859, 1871, 1876, 1877), (2) fossil seal specimens that were studied by Van Beneden (1877) but not illustrated, as well as (3) material that has been collected by or donated to the IRSNB in the course of the 20th century. Geographic and stratigraphical data associated with these specimens are of uneven scientific value: for some specimens a relatively precise and accurate positioning can be retrieved, while for other specimens no information exists at all.

#### Recently acquired specimens

The recent acquisition of a number of specimens attributed to “*Phoca*” *vitulinoides* directly spurred the re-investigation of this species. These acquisitions include the “Gommers–Bosselaers specimen” (IRSNB M2276a–q), specimens recently found at the Antwerp International Airport (IRSNB M2272, IRSNB M2273, IRSNB M2274, and IRSNB M2275), and specimens from the Gigase collection (IRSNB M2269, IRSNB M2270, and IRSNB M2271). The Gommers–Bosselaers specimen is the most complete specimen of fossil seal from the Neogene of the Antwerp area (and the whole North Sea Basin), containing seventeen bones attributed to a single individual: the dens of the axis, five thoracic vertebrae, two lumbar vertebrae, the sacrum, one caudal vertebra, the head and neck of the right scapula, the complete left humerus and the distal half of the right humerus, the complete left and right femora, the proximal half of the right tibia, and the right fourth metatarsal with the distal extremity unfused. Dutch fossil collector Henny Gommers recovered the specimen in the 1980s, during road works along the Antwerp R1 ring road. Mark Bosselaers subsequently acquired the specimen in 2015 and donated it to the IRSNB. The geographic and stratigraphic data of this specimen are described and discussed in the corresponding sections.

Material from the Antwerp International Airport (IATA: ANR – ICAO: EBAW) has been collected during construction works at the airport in 2015 by a group of private collectors, including Luc Anthonis, Bert Gijsen, and Frederik Mollen. A total of approximately 60 m^3^ of scooped sediment has been sieved and yielded isolated and associated bones that can be attributed to several individuals of “*Phoca*” *vitulinoides*. Selected specimens have been donated to the IRSNB. The geographic and stratigraphic data of these specimens are described and discussed in the corresponding sections.

Father and son, Paul and Gigase are long-time collectors of fossil vertebrates, including marine mammals from the Antwerp region. With a fossil pinniped collection totaling more than one hundred specimens, the Gigase collection includes numerous isolated bones that were attributed to the species “*Phoca*” (*Nanophoca*) *vitulinoides*. The Gigase donated relevant specimens from their private collection to the IRSNB (see “referred specimens”).

The majority of the specimens have been found isolated. Because of their diagnostic value, isolated humeri and femora can easily be tied to “*Phoca*” (*Nanophoca*) *vitulinoides*. However, the ribs, the radius, the ulna, and the calcaneum are only known from relatively isolated bones. Their assignment to “*Phoca*” (*Nanophoca*) *vitulinoides* remains highly tentative, because they are neither known for other contemporaneous small phocine seals from the North Sea basin (*Batavipusa neerlandica* and *Praepusa boeska*). Similarly, the neotype specimen of the axis of “*Phoca*” (*Nanophoca*) *vitulinoides* (IRSNB M2276i) is very incompletely preserved. A better-preserved specimen (IRSNB M2268) has been found isolated. The assignment of the latter axis to the species is based on its comparable size to the neotype specimen and, hence, tentative.

#### Comparative material

Comparative specimens of extant and extinct taxa are listed as Supplemental Information. Extant taxa are listed as List S1 and extinct taxa are listed as List S2.

### Measurements and body length estimates

Measurements were taken to the nearest 0.1 mm, using analog calipers. For reasons of consistency, these measurements were taken following the same scheme as Koretsky (2001), which has also been applied to *L. proxima*, *P. etrusca,* and *Prophoca rousseaui* more recently (Berta et al., 2015; Dewaele, Lambert & Louwye, 2017). Measurements are presented in Tables S1–S8.

Regarding the body length estimates of the species, a number of published dissections of Phocidae mention the relationship between lengths of individual long bones and total body length (snout-to-tail length). Dissected species and references considered include *Pusa hispida* (as *Phoca hispida*; Howell, 1929), *Leptonychotes weddelli* (Piérard, 1971), and *Ommatophoca rossi* (Piérard & Bisaillon, 1975). For the body length estimates of *N. vitulinoides*, long-bone-to-total-body-length ratios of the aforementioned species are extrapolated for long bone length measurements of *N. vitulinoides*. This is partly in accordance with the body length estimate of another diminutive fossil seal *Australophoca changorum* Valenzuela-Toro, Pyenson, Gutstein, & Suárez, 2015, for which the authors used Howell’s dissection of *Pusa hispida* (Howell, 1929). Additionally, the humerus length to total body length ratio and the femur length to total body length ratio have been calculated for specimens of *Phoca vitulina* (*n* = 5) and *Pusa sibirica* (*n* = 1). These additional ratios are also used to estimate the total body length of *N. vitulinoides*.

### Terminology

In order to be consistent with other recent publications on extinct Phocidae, we adopted the nomenclature and terminology used by Amson & Muizon (2014), Berta et al. (2015), and Dewaele, Lambert & Louwye (2017) to describe the morphological anatomy. Whenever it was not possible to refer to these, we adopted the nomenclature and terminology for the osteological description of the domestic dog by Evans & de Lahunta (2013).

For myological inferences, we refer to published dissections of the ringed seal *Pusa hispida*, the Southern elephant seal *Mirounga leonina* (Linnaeus, 1758), the Weddell seal *L. weddelli*, and the Ross seal *O. rossi* (Howell, 1929; Bryden, 1971; Piérard, 1971; Piérard & Bisaillon, 1975). We also use the myological inferences made for the extinct monachines *A. longirostris* and *P. pacifica*, and their locomotive interpretations (Muizon, 1981).

### Phylogenetic analysis

The phylogenetic analysis was performed using PAUP version 4.0b10 for Macintosh (Swofford, 2001) with a heuristic search option with simple sequence addition, using the tree-bisection-reconnection (TBR) algorithm. Bootstrap values were obtained after a full heuristic search with 10,000 replications with random number seed zero and the best tree saved for each replication. Character states were optimized with accelerated transformation criterion (ACCTRAN). For the Goloboff criterion the *k*-value was set at 3. Formerly, different character matrices resulting in different phylogenetic trees have been used to elucidate the phylogenetic relationships among Phocidae (see, e.g., Bininda-Emonds & Russell, 1996; Koretsky, 2001; Koretsky & Rahmat, 2013; Amson & Muizon, 2014; Berta et al., 2015). In this study, we use 85 morphological characters, either newly described, adopted, or adapted from published phylogenetic analyses incorporating Phocidae (Berta & Wyss, 1994; Bininda-Emonds & Russell, 1996; Cozzuol, 2001; Koretsky, 2001; Koretsky & Grigorescu, 2002; Koretsky & Rahmat, 2013; Amson & Muizon, 2014; Berta et al., 2015; Koretsky, Peters & Rahmat, 2015) (List S3). One character is parsimony-uninformative (24) and three (32, 33, 81) are ordered. A significant number of the phylogenetic characters scored by Koretsky (2001) and Koretsky & Rahmat (2013) are prone to subjective scoring (e.g., character states “deep” versus “shallow”). Therefore, only a limited number of those characters have been adopted for the current analysis. Time-calibration of the phylogenetic analyses presented in this study has been performed by time-fixating the nodes that have been recovered in the molecular phylogenetic analysis from Higdon et al. (2007).

The analysis includes 31 operational taxonomic units (OTUs). Outgroups include the early Miocene pinnipedimorph *Enaliarctos mealsi* Mitchell & Tedford, 1973 and the pinnipediform *Pteronarctos goedertae* Barnes, 1989, the extant South American sea lion *Otaria byronia* Blainville, 1820 and the extinct otariid *Thalassoleon mexicanus* Repenning & Tedford, 1977, and the desmathophocid *Allodesmus kernensis* Kellogg, 1922. Information on outgroup OTUs included in the phylogenetic analysis is based on personal observations and descriptions in the relevant literature. Ingroup taxa include representatives of all extant phocid genera: the Monachinae *Hydrurga leptonyx*, *L. weddelli*, *Lobodon carcinophaga*, *Mirounga leonina*, *Monachus monachus*, and *O. rossi*; and the Phocinae *C. cristata*, *Erignathus barbatus*, *Halichoerus grypus*, *Histriophoca fasciata*, *Pagophilus groenlandicus*, *Phoca vitulina*, *Pusa caspica*, *Pusa hispida*, and *Pusa sibirica*. Extinct phocid taxa included in the analysis are limited to the lobodontin Monachinae *H. martini* Amson & de Muizon, 2013 and *P. pacifica* Muizon, 1981; the Devinophocinae Koretsky & Holec, 2002 *D. claytoni* Koretsky & Holec, 2002 and *Devinophoca emryi* Koretsky & Rahmat, 2013; and the Phocinae *Kawas benegasorum* Cozzuol, 2001, *L. proxima* (Van Beneden, 1877, *Praepusa boeska* Koretsky, Peters & Rahmat, 2015, *Praepusa magyaricus* Koretsky, 2003, *Praepusa pannonica* Kretzoi, 1941, *Praepusa vindobonensis*, and “*Phoca*” (*Nanophoca*) *vitulinoides*. The character matrix is provided as Data S1. We follow Barnes (1972) in considering *Allodesmus kelloggi* as a junior synonym of *Allodesmus kernensis*. Extant Phocidae, *L. proxima*, *Praepusa boeska*, and “*Phoca*” (*Nanophoca*) *vitulinoides* were scored after personal observation. *Praepusa vindobonensis* has been scored after on-hand observations of casts at the USNM, and illustrations and descriptions by Toula (1897) and Koretsky (2001). Koretsky (2001) assigned different isolated cranial, mandibular, and postcranial bones to *Praepusa vindobonensis* on the basis of an ecomorphotype hypothesis presented in her publication. Pending the discovery of new associated or articulated cranial and mandibular material of *Praepusa vindobonensis*, we tentatively score mandibular and cranial characters of *Praepusa vindobonensis* on the basis of mandibles and skulls currently housed in the IZUAN collection, but coding is based on descriptions and illustrations from Koretsky (2001) and are not readily adopted from her character matrix. Character states of other OTUs are scored on the basis of illustrations and descriptions in the literature (Mitchell, 1966; Barnes, 1972; Mitchell & Tedford, 1973; Berta & Ray, 1990; Cozzuol, 2001; Koretsky, 2001; Koretsky & Holec, 2002; Koretsky, Peters & Rahmat, 2015; Koretsky & Rahmat, 2015; Rahmat & Koretsky, 2016; Dewaele, Lambert & Louwye, 2017).

### Nomenclatural acts

The electronic version of this article in portable document format (PDF) will represent a published work according to the International Commission on Zoological Nomenclature (ICZN), and hence the new names contained in the electronic version are effectively published under that code from the electronic edition alone. This published work and the nomenclatural acts it contains have been registered in ZooBank, the online registration system for the ICZN. The ZooBank life science identifiers (LSIDs) can be resolved and the associated information viewed through any standard web browser by appending the LSID to the prefix http://zoobank.org. The LSID for this publication is: urn:lsid:zoobank.org:pub:1310A48E-A725-40E7-AFFB-D0A9043CFE04. The online version of this work is archived and available from the following digital repositories: PeerJ, PubMed Central, and CLOCKSS.

## Results

### Geological context

#### Lithostratigraphy

**Figure 2:**
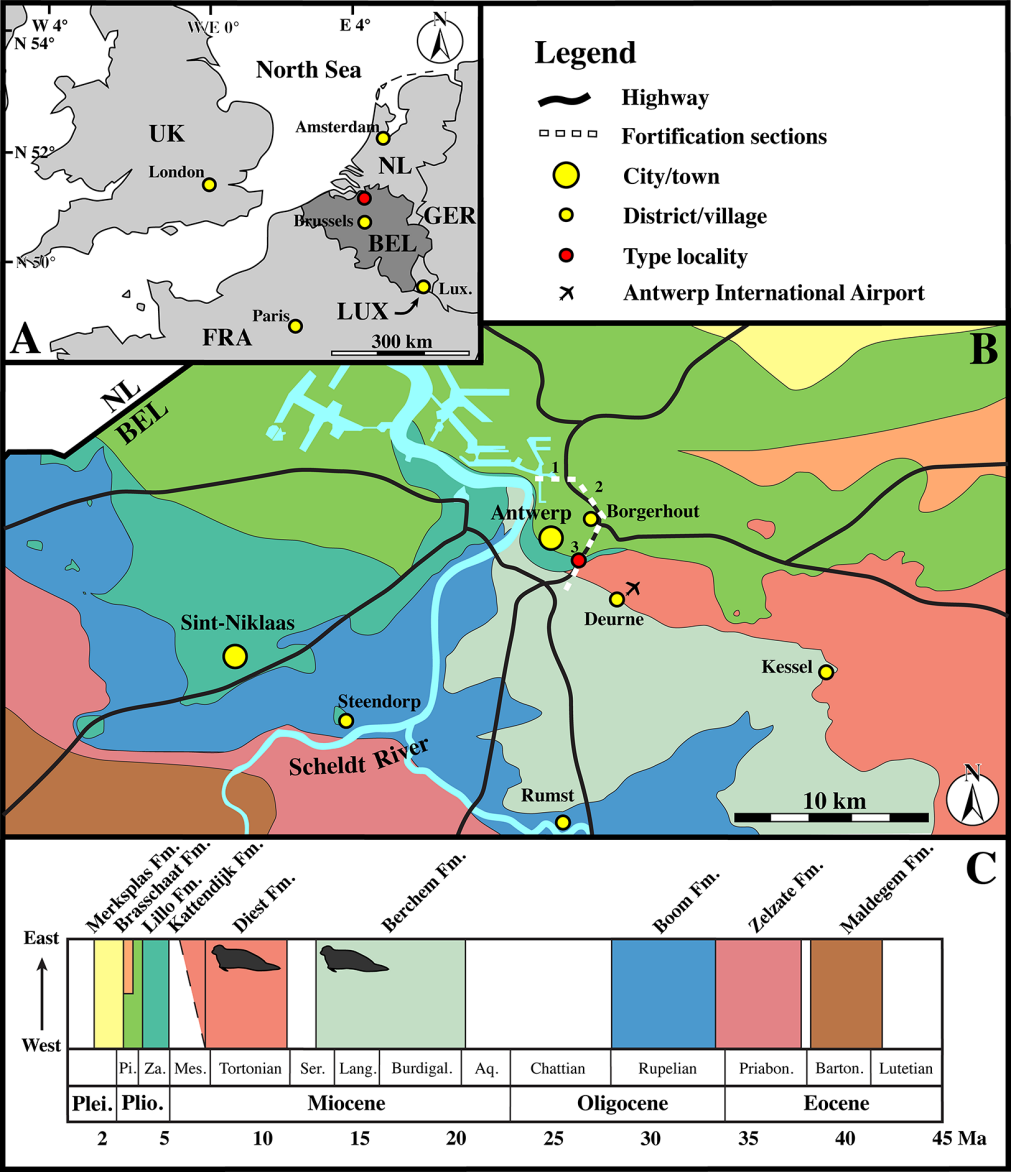
Localities. (A) Regional map showing the southern part of the North Sea basin with bordering countries and labeled capital cities (yellow) and the Antwerp area (red). (B) Close-up of the Antwerp area with color-coding for the outcropping Paleogene and Neogene strata underneath the Quaternary top layer. The sections of the fortification walls around Antwerps used by Van Beneden (1877) as localities for the Neogene marine mammals (including seals) from the Antwerp area are indicated by dashed lines and numbered as in Van Beneden (1877), using Vanden Broeck (1878). (C) Stratigraphic legend for the Paleogene and Neogene strata from the Antwerp Area, with small seals indicating the stratigraphic occurrence of the neotype (IRSNB M2276) and other recently discovered specimens of *Nanophoca vitulinoides* Abbreviations: NL, Netherlands; GER, Germany; LUX, Luxembourg; FRA, France; UK, United Kingdom; BEL, Belgium; Lux., Luxembourg City; Plei., Pleistocene; Plio., Pliocene; Pi., Piacenzian; Za., Zanclean; Mes., Messinian; Ser., Serravallian; Lang., Langhian; Burdigal., Burdigalian; Aq., Aquitanian; Priabon., Priabonian; Barton., Bartonian; Fm., Formation. Image based on data from Dienst Ondergrond Vlaanderen (DOV; dov.vlaanderen.be).

Specimens of “*Phoca*” *vitulinoides* have been recovered (and described) over the course of more than a century, from a number of locations and by a number of different collectors. Historically, the specimens of “*Ph*.” *Vitulinoides* from the Van Beneden collection (1871, 1876, 1877) were collected by the military during the 1860s fortification works around the city of Antwerp (Van Beneden, 1877). Specimens studied by Van Beneden hence came either from construction sites at forts or from different “sections” (i.e., trenches) around the city of Antwerp. These sections have been numbered, with section 1 representing the section north of Antwerp, section 2 northeast of Antwerp, and section 3 east of Antwerp (Fig. 2) (Vanden Broeck, 1878). The location of these sections roughly coincides with that of today’s highway R10 around Antwerp. Van Beneden (1876, 1877) assigned a “Scaldisien” (Scaldisian) age to the specimens of “*Ph*.” *Vitulinoides* he studied. However, the Scaldisian is currently considered an obsolete term (Laga & Louwye, 2006) and there appears to be confusion about what the Scaldisian represented (Laga & Louwye, 2006; and references therein). Although Van Beneden never provided lithostratigraphic data, it is generally accepted that the Scaldisian Van Beneden used to date “*Ph*.” *Vitulinoides* in fact refers to the basal gravel of the Zanclean (early Pliocene) Kattendijk Formation (e.g., Koretsky & Ray, 2008; P. Gigase, 2015, personal communication). Hence, all species Van Beneden “dated” to the Scaldisian are currently considered to be of early Pliocene age or older. However, no lithostratigraphic or biostratigraphic evidence supports this assumption.

Collections from the IRSNB that have not been studied by Van Beneden include the Hasse collection, which entered the IRSNB collection decades after Van Beneden’s work. Quality of stratigraphic data associated with specimens of these collections is low and Hasse’s specimens have been “dated” to the “Boldérien” (Bolderian). As with the Scaldisian, the term Bolderian has currently been abandoned and should not be used anymore (Laga & Louwye, 2006). Following Table 1 in Laga & Louwye (2006), it appears that some researchers considered the early to middle Miocene Berchem Formation to represent the Bolderian stage. In the region of Kessel, where the specimens of the Hasse collection come from, the Berchem Formation crops out (Fig. 2). Sediment recovered from the sacral canal of IRSNB M2277 from the Hasse collection has been dated biostratigraphically using dinoflagellate cysts (see section below).

The collection from the site at the Antwerp International Airport has a detailed stratigraphic framework (Hoedemakers & Dufraing, 2015). The specimens have been recovered from the layer V, overlying the Antwerpen Sands Member of the Berchem Formation and underlying the Deurne Sands Member of the Diest Formation (Hoedemakers & Dufraing, 2015). This layer V has a relatively high vertebrate fossil content, but has not yet been formally studied and dated. However, bracketed by dated sediments of the Berchem and Diest Formations, its age must range between late Serravallian and early Tortonian (late middle to early late Miocene).

Specimens of “*Phoca*” (*Nanophoca*) *vitulinoides* from the Gigase collection from different localities are associated with relatively detailed stratigraphic data. Many of them come from a basal gravel, which has sometimes been identified as the basal gravel of the Kattendijk Formation. Gigase also tentatively assumes that a number of these specimens was reworked from—presumably—Miocene deposits on the basis of their state of preservation, which often consists of abrasion (P. Gigase, 2015, personal communication). One specimen has been found in situ in Miocene deposits: IRSNB M2270 in the Deurne Sands Member of the Diest Formation.

No stratigraphic data has been provided with the Gommers–Bosselaers specimen. However, the geographic location is precisely known: just northwest of the crossing of the Zurenborgbrug (bridge) over the R10 highway. Studying a section at the site is hampered by the presence of a highly disturbed top layer and a dense network of tree roots. Stratigraphic inferences are based on the study of two stratigraphic drillings carried out by the Geological Survey of Belgium (available at Databank Ondergrond Vlaanderen; http://www.dov.vlaanderen.be) and one section (section I B.P.) presented by De Meuter, Wouters & Ringele (1976), from within a 50 m radius of the locality. Both drillings show approximately 1.5 m of (disturbed) quaternary sediments on top of 1.5–2 m of brownish to greenish glauconitic sands from the Berchem Formation, which become greener with increasing depth. In one drilling, the Berchem Formation has been specified to the Antwerpen Sands Member. Similarly, De Meuter, Wouters & Ringele (1976) interpret section I B.P. as the Antwerpen Sands [Member] subsequently covered by reworked Deurne Sands [Member of the Diest Formation] and reworked Kattendijk Sands [i.e., Kattendijk Formation], a lumachelle layer representing reworked Lillo Formation, Quaternary, and “Filling up.” The neotype of *N. vitulinoides* has been recovered from a slope, about two meters below the top of the slope. While the drillings were on top of this slope, the exact location of section I. B.P. in relation to this slope is unknown. However, shell fragments are abundant on the slope at and above the level of the locality of the neotype. Hence, all indications points toward the Berchem Formation (and presumably the Antwerpen Sands Member) as the stratigraphic origin of the neotype IRSNB M2276.

#### Dinoflagellate cyst biostratigraphy

Two sediment samples (sample 1018/1019 from the sacrum of *N. vitulinoides* IRSNB M2276a and sample 1026 from the sacrum IRSNB VERT-8243-07, the latter being not figured in this study) recovered from bone cavities were palynologically analysed for organic-walled dinoflagellate cysts (dinocysts) and acritarchs (Table S9). The palynological preparation of the sediments followed standard techniques described by Louwye, Head & De Schepper (2004). Acid treatments with HCl and HF were applied for the removal of carbonates and silicates, respectively. Sieving of the organic residue was carried out on a nylon screen with a 10 μm mesh size. The residue was placed on glass slides with glycerol gelatine jelly. The microscopic analysis was carried out with a transmitted light microscope Zeiss AxioImager A1 under a 400× magnification. The entire slide was scanned in non-overlapping traverses. The taxonomy of the dinocysts and acritarchs follows Fensome, MacRae & Williams (2008).

The preservation and diversity of the dinocysts in sample 1018/1019 is moderate to good. A total of 21 dinocyst species and one acritarch were recorded (Table S9). A maximum age for the sample is provided by the key species *Habibacysta tectata*, with a lowest occurrence in high latitudes dated at 14.2 Ma (Schreck, Matthiesen & Head, 2012), a datum later confirmed by Quaijtaal et al. (2014) in lower latitudes (Porcupine Basin, off southwest Ireland). A minimum age for the sample is given by *Cleistosphaeridium placacanthum*; several authors report a highest occurrence of this key species in the Serravallian of the North Sea Basin and the North Atlantic realm: mid-Serravallian (chron C5Abn) of New Jersey, USA (de Verteuil & Norris, 1996); mid Serravallian (12.8 Ma) offshore Denmark (Dybkjær & Piasecki, 2010); middle-upper Serravallian of the southern North Sea Basin (Munsterman & Brinkhuis, 2004); and upper Serravallian of the Porcupine Basin offshore southwest Ireland (Louwye et al., 2008). The sediment samples 1018/1019 retrieved from the sacrum IRSNB M2276a are thus not older than late Langhian (14.2 Ma) and not younger than late Serravallian (middle Miocene).

The preservation and diversity of the dinocysts in sample 1026 are poor. Only ten dinocyst species and one acritarch were recorded. Only two dinocysts can be considered as biostratigraphic key species. The lowest occurrence of *H. tectata* has been dated at 14.2 Ma (see above). The presence of *C. placacanthum* provides a minimum age in the late Serravallian (see above). The sediment retrieved from the sacrum IRSNB VERT-8243-07 found at Kessel has thus an age between late Langhian (14.2 Ma) and late Serravallian, corroborating the middle Miocene age of the first sample.

#### Systematic paleontology

Family PHOCIDAE Gray, 1821
Subfamily PHOCINAE Gray, 1821
*NANOPHOCA* gen. nov.

**Type and only included species:** *Nanophoca vitulinoides* (Van Beneden, 1871).

**Diagnosis of genus:** As for the type and only species.

**Etymology:** The name of the genus is derived from the Greek nouns “nanos” (m.), meaning “dwarf,” and “phoké” (f.), meaning “seal.” This name highlights the small size of this seal genus.

*NANOPHOCA VITULINOIDES*. (Van Beneden, 1871)
*Phoca vitulinoides* Van Beneden, 1871.
*Phoca* (*Phoca*) *vitulinoides* Friant, 1944.
“*Phoca*” *vitulinoides* Koretsky & Ray, 2008.

##### Neotype

IRSNB M2276a–q, including the dens of the axis (i), two middle thoracic vertebrae (j, k), two posterior thoracic vertebrae (l, m), three lumbar vertebrae (n, o, p), sacrum (a), ?one caudal vertebra (q), partial right scapula (f), complete right (c) and partial left humeri (b), right and left femora (d, e), partial right tibia (g), and the right fourth metatarsal (h) of a single individual (Fig. 1A).

##### Type locality

North of the Zurenborgbrug and between the R10 road and E19 highway, Berchem District, Antwerp, Antwerp Province, Belgium (Fig. 2).

##### Type horizon and age

A sediment sample recovered from the sacral canal of the Gommers–Bosselaers specimen has been subjected to dinoflagellate cyst biostratigraphy (see section below). Dinoflagellate cyst biostratigraphy of a sediment sample recovered from the sacrum of IRSNB M2276a yield a minimum age ranging from late Langhian to late Serravallian (middle Miocene) age for the neotype of *N. vitulinoides*.

##### Diagnosis

*Nanophoca vitulinoides* is a small seal, estimated to have reached a length of approximately one meter, which is slightly smaller than members of the genus *Pusa* (1.3 m for male *Pusa sibirica* Ciesielski et al., 2006). It differs from other genera of Phocinae in the following characteristics: sacrum with three (also in Monachinae Gray, 1869) to four fused sacral vertebrae (also in other Phocinae); sacral spinous processes fused and dorsally elongate; prominent hook-like ischiatic spine; and a low proximodistally oriented ridge just proximal to the medial condyle of the femur, less than one millimeter raised over the condyle. Additionally, the following characteristics have also been observed in other Phocinae, but their combination is unique to *N. vitulinoides*: a scapular spine and subspinous ridge (see definition below) fuse at scapular neck (also in *H. grypus*, *Phoca*, and *Pusa*) lesser tubercle of humerus at same level as humeral head (also in *Cryptophoca maeotica*); greater trochanter of femur higher than head (also in *Praepusa vindobonenesis*, *Pusa caspica*, and *Pusa sibirica*); head of femur on narrow, long neck (also in *C. maeotica*, *L. proxima*, *Monachopsis pontica*, *Praepusa vindobonensis*, and *Sarmatonectes sintsovi*).

##### Referred specimens

**Figure 3:**
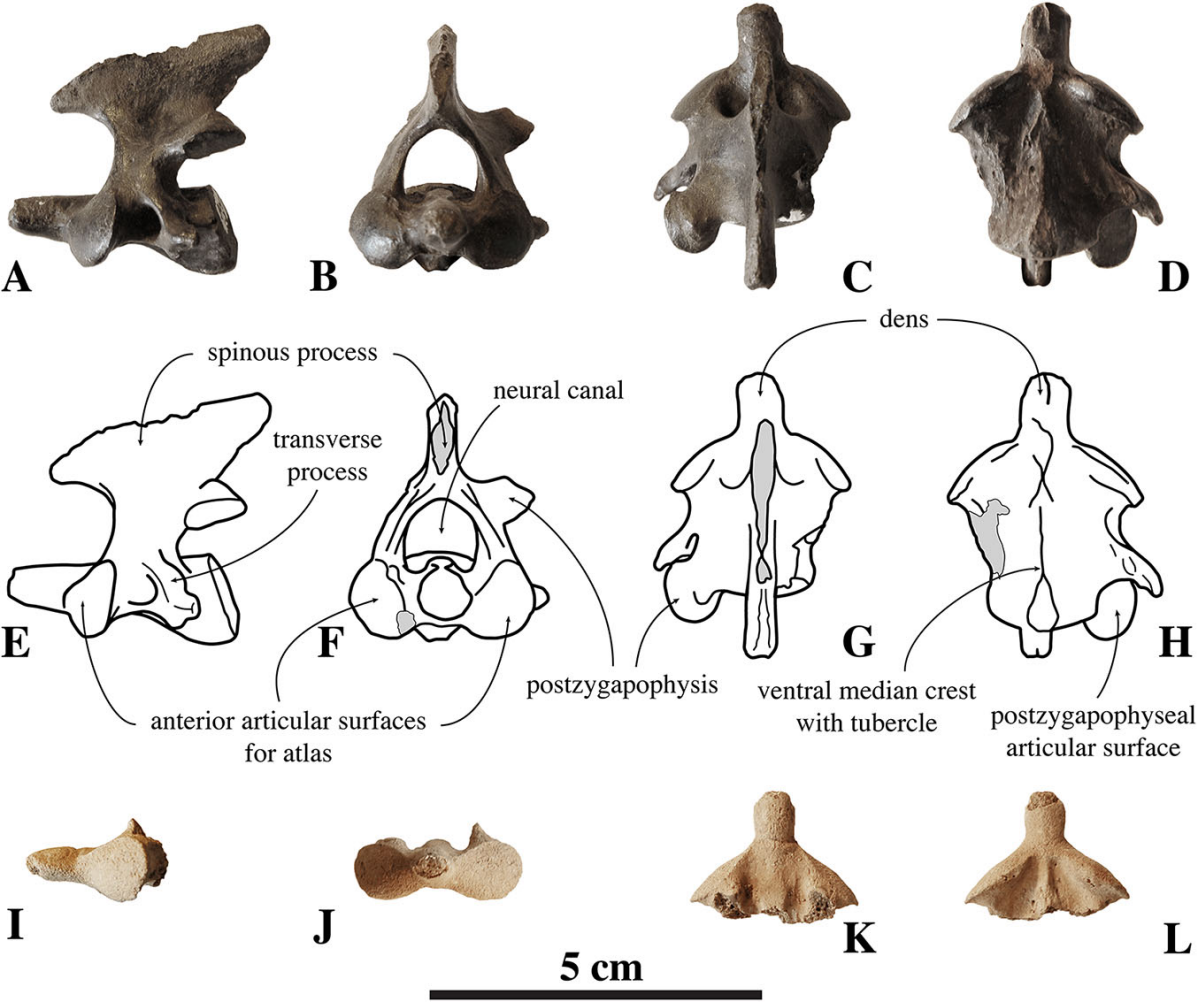
Axes of *Nanophoca vitulinoides*. IRSNB M2268 axis of *Nanophoca vitulinoides* (A–D) and corresponding drawings (E–H) in left lateral (A, E), anterior (B, F), dorsal (C, G), and ventral (D, H) view. IRSNB M2276i (neotype) axis of *Nanophoca vitulinoides* in left lateral (I), anterior (J), dorsal (K), and ventral (L) view. Broken or obliterated areas are indicated in gray.

**Figure 4:**
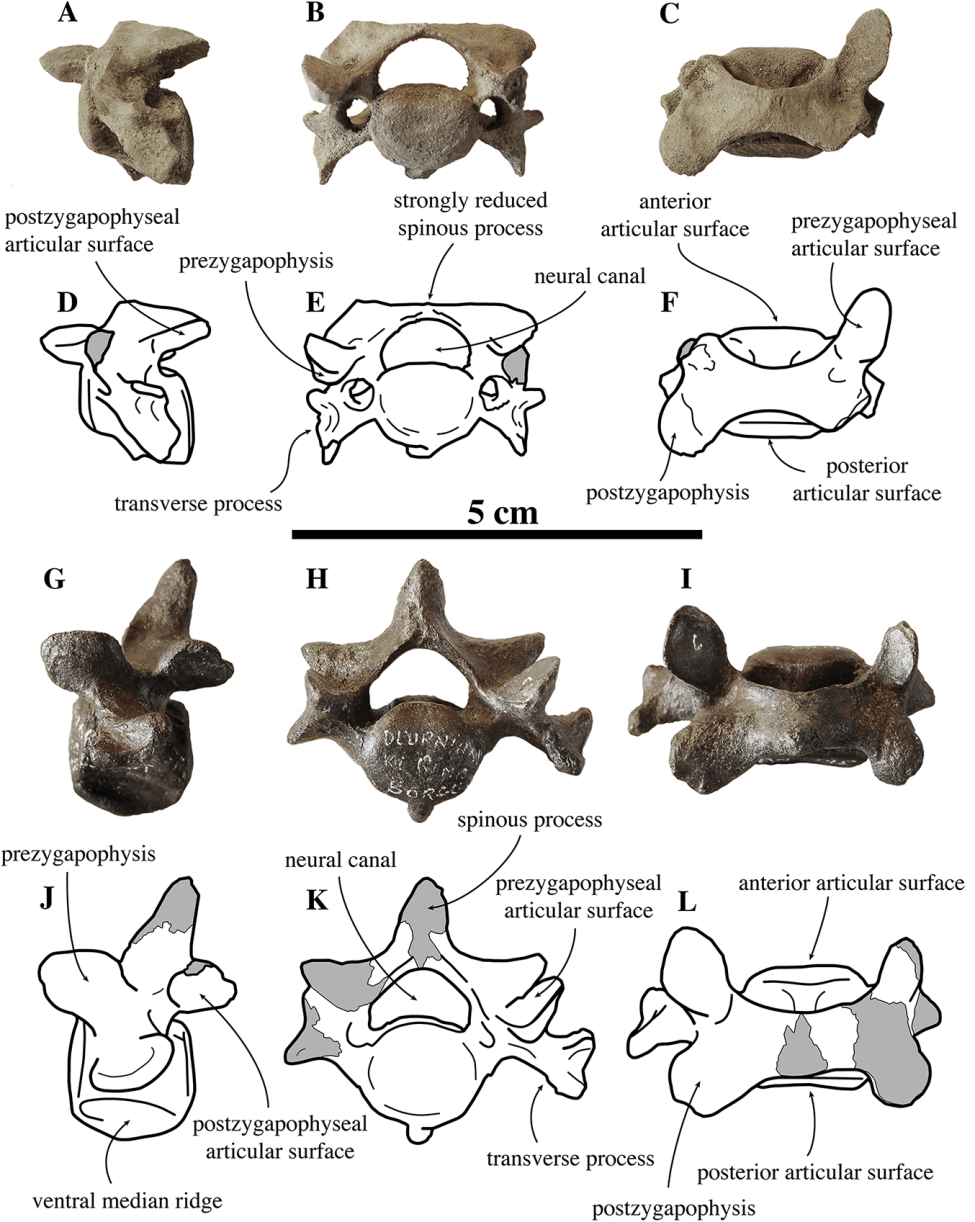
Other cervical vertebrae of *Nanophoca vitulinoides*. IRSNB M2274 third cervical of *Nanophoca vitulinoides* (A–C) and corresponding drawings (D–F) in left lateral (A, D), anterior (B, E), and dorsal (C, F) view. IRSNB M2270 seventh cervical vertebra of *Nanophoca vitulinoides* (G–I) and corresponding drawings (J–L) in left lateral (G, J), anterior (H, K), and dorsal view (I, L). Broken or obliterated areas are indicated in gray.

Associated or articulated referred specimens: **IRSNB M2276a–q**, neotype, partial skeleton with dens of axis (i), two middle thoracic vertebrae (j, k), two posterior thoracic vertebrae (l, m), three lumbar vertebrae (n, o, p), sacrum (a), ?one caudal vertebra (q), partial right scapula (f), complete right (c) and partial left (b) humeri, right and left femora (d, e), partial right tibia (g), and the right fourth metatarsal (h); from the Berchem Formation from N of the Zurenborgbrug, Antwerp, Belgium, and collected by H. Gommers and donated to the IRSNB by M. Bosselaers. **IRSNB 1059-M240a–f**, pelvic girdle with three lumbar vertebrae (d–f), sacrum (b), left and right innominates (a), and left femur (c), from the “Scaldisian” of section 3 at Borgerhout, Belgium, and illustrated by Van Beneden (1877, pl. XV, Figs. 1–4, 17, 18) (Fig. 1B). **IRSNB 1066-M243a–c**, right radius (a), right ulna (b), and right rib (c), from the “Scaldisian” of section 3 at Antwerp and illustrated by Van Beneden (1877, pl. XV, Figs. 10, 11, 29) (Fig. 1C). **IRSNB 1226-M244a,b**, one anterior thoracic vertebra (b), and left innominate (a), from the “Scaldisian” of section 3 at ?Borgerhout and illustrated by Van Beneden (1877, pl. XV, Fig. 12).

**Figure 5:**
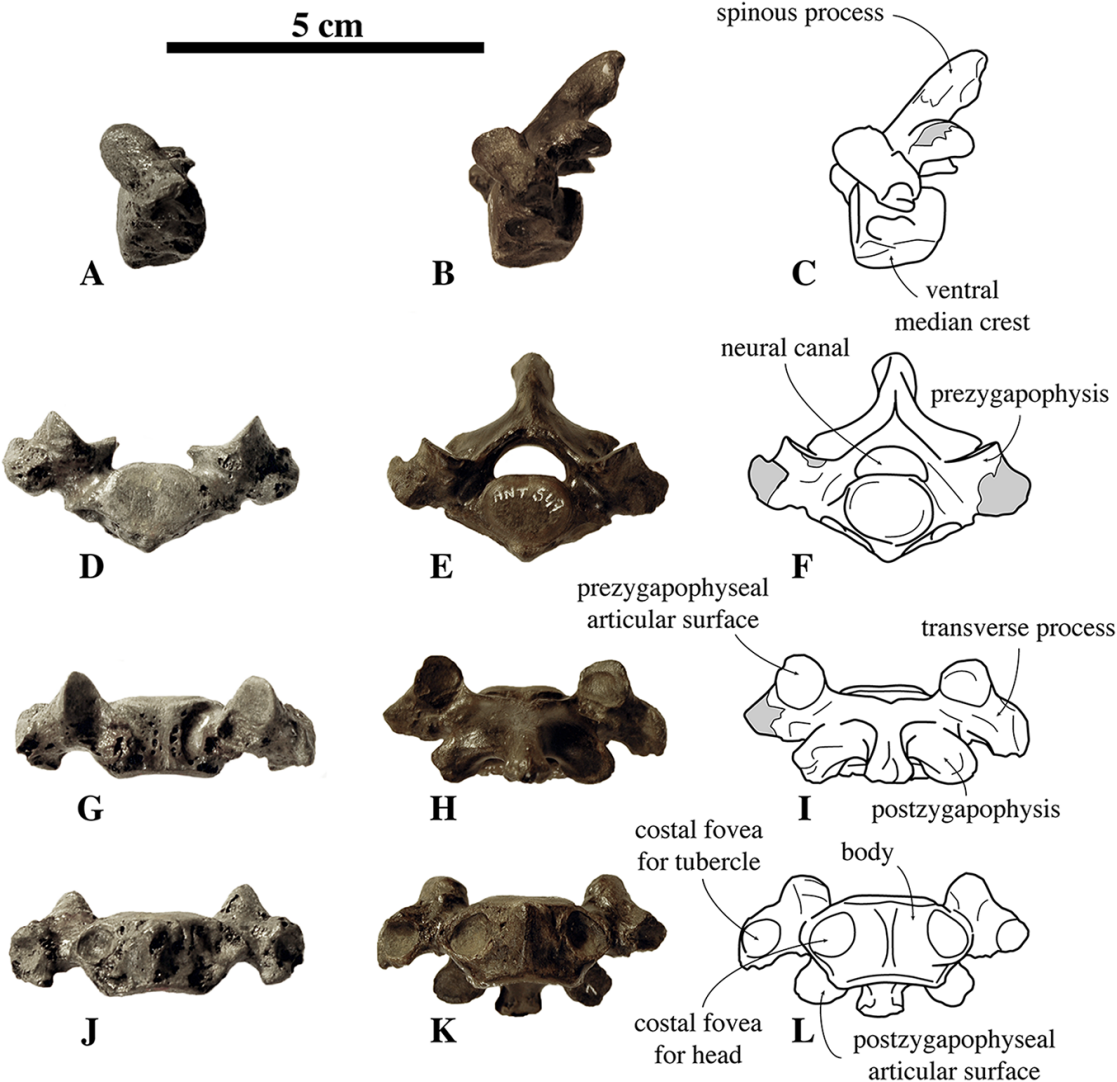
Anterior thoracic vertebrae of *Nanophoca vitulinoides*. IRSNB 1226-M244b (A, D, G, J) and IRSNB M2269 (B, E, H, K) anterior thoracic vertebrae of *Nanophoca vitulinoides*; and corresponding drawings of the latter (C, F, I, L) in left lateral (A–C), anterior (D–F), dorsal (G–I), and ventral (J–L) view. Broken or obliterated areas are indicated in gray.

**Figure 6:**
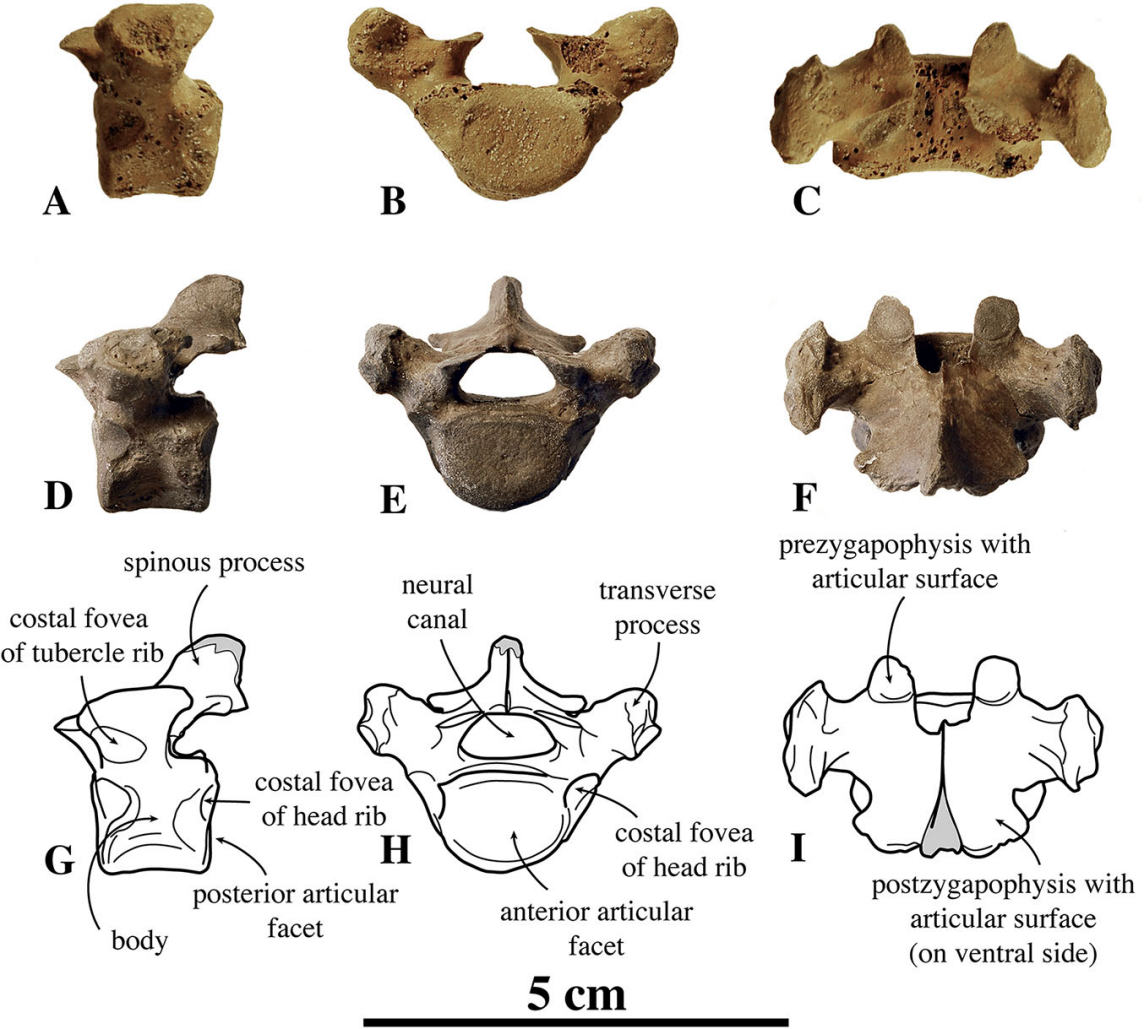
Middle thoracic vertebrae of *Nanophoca vitulinoides*. IRSNB M2276k (neotype) (A–C), IRSNB 1075-M245 (D–F) middle thoracic vertebrae of *Nanophoca vitulinoides*, and corresponding drawings of the latter (G–I); in left later (A, D, G), anterior (B, E, H), and dorsal (C, F, I) view. Broken or obliterated areas are indicated in gray.

**Figure 7:**
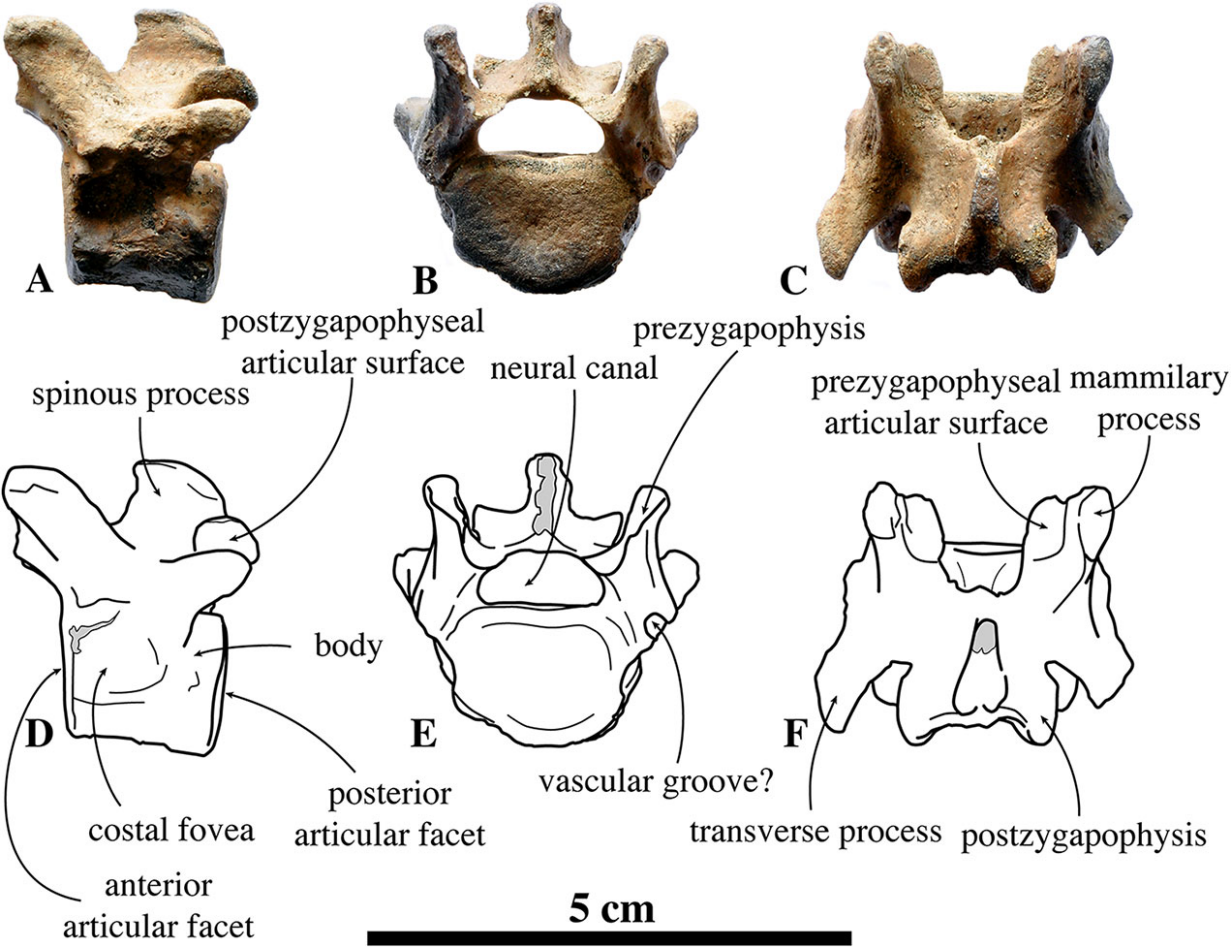
Posterior thoracic vertebra of *Nanophoca vitulinoides*. IRSNB M2273 posterior thoracic vertebra of *Nanophoca* (A–C) and corresponding drawings (D–F), in left lateral (A, D), anterior (B, E), and dorsal (C, F) view. Broken or obliterated areas are indicated in gray.

**Figure 8:**
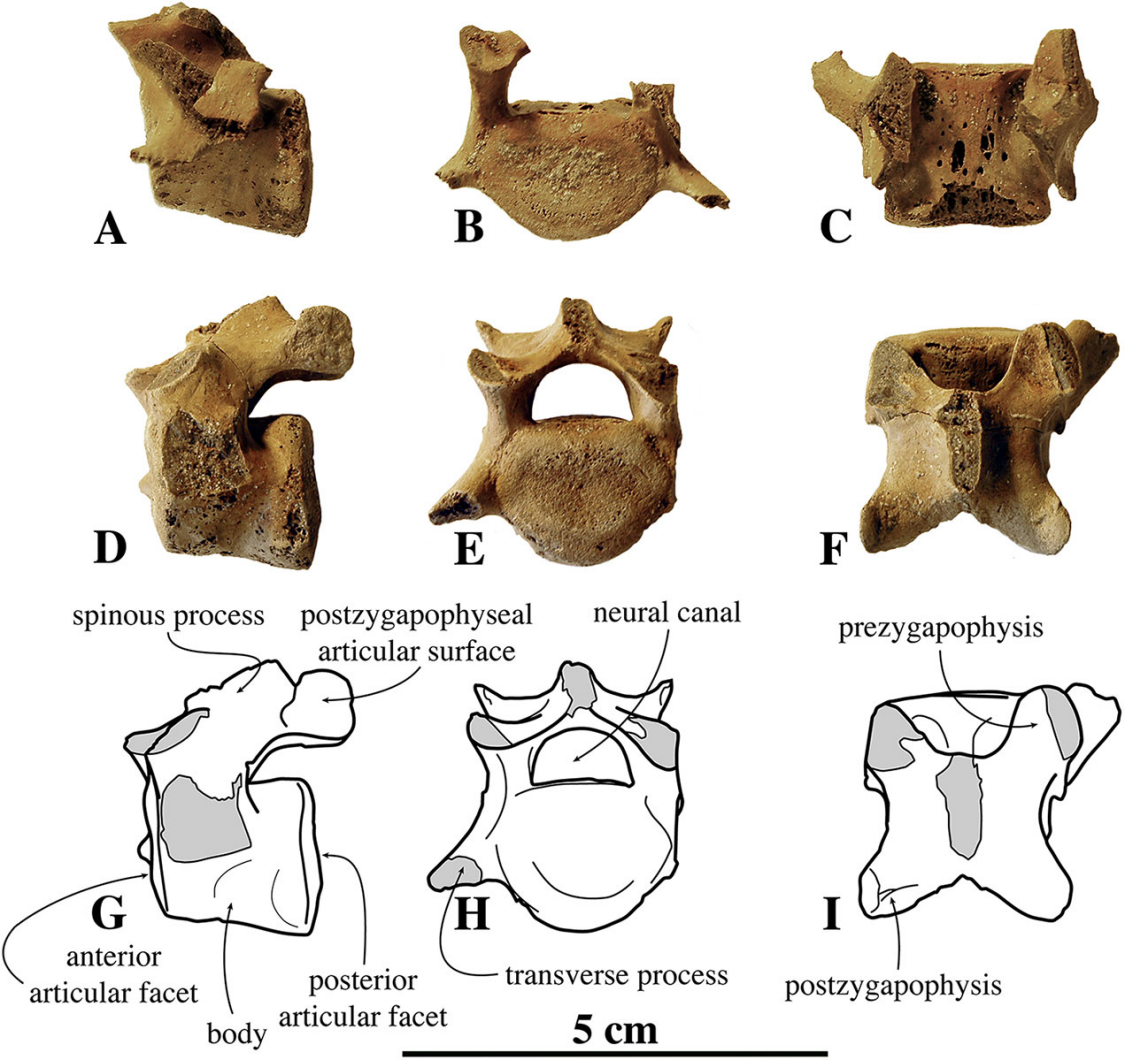
Lumbar vertebrae of *Nanophoca vitulinoides*. IRSNB M2276n (neotype) (A–C), IRSNB M2276o (neotype) (D–F), and corresponding drawings of the latter (G-I); in left lateral (A, D, G), anterior (B, E, H), and dorsal (C, F, I) view. Broken or obliterated areas are indicated in gray.

**Figure 9:**
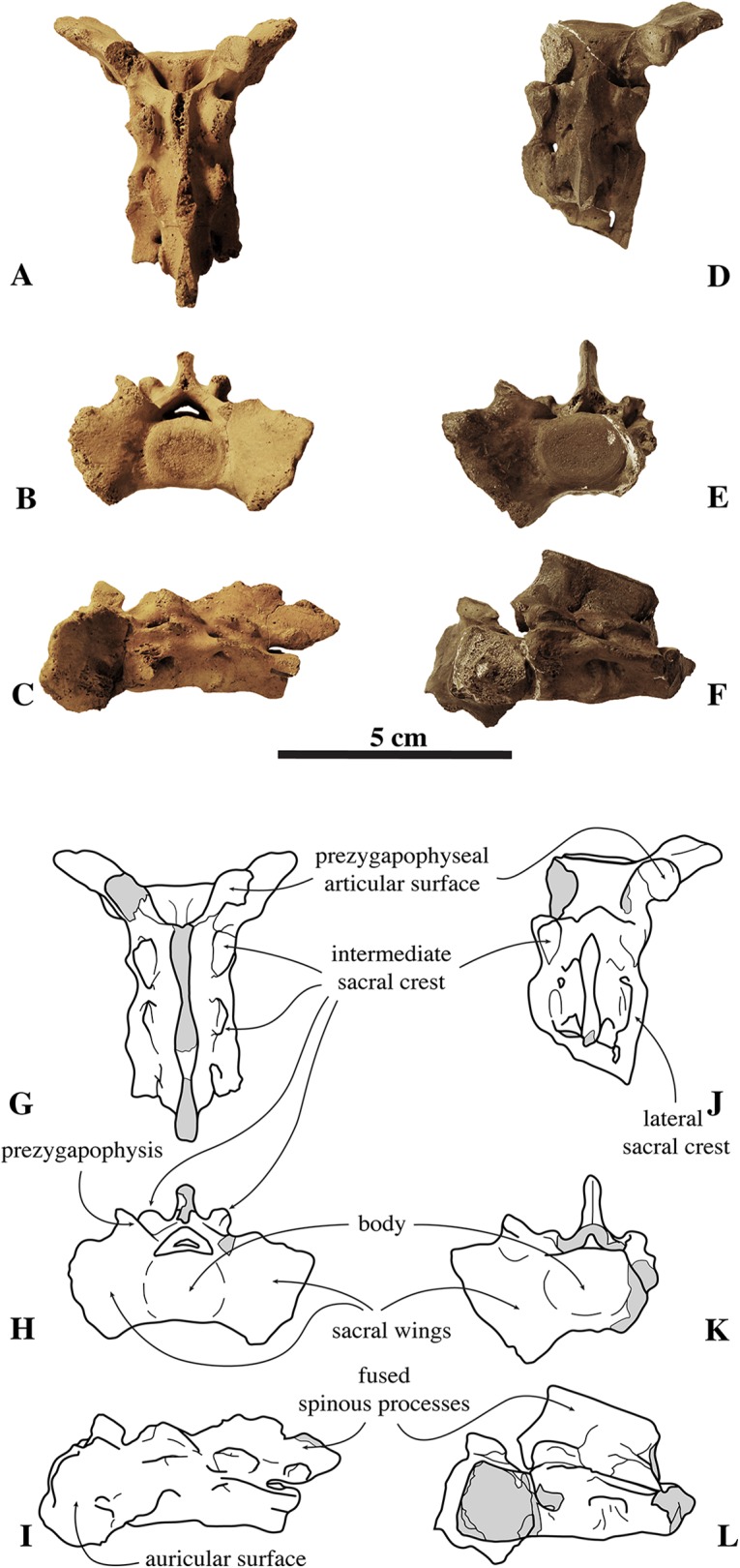
Sacra of *Nanophoca vitulinoides*. IRSNB M2276a (neotype) (A–C) and IRSNB M2277 (D–F) sacra of *Nanophoca vitulinoides*, and corresponding drawings (G–I; J–L) in dorsal (A, G; D, J), anterior (B, H; E, K), and left lateral (C, I; F, L) view. Broken or obliterated areas are indicated in gray.

Isolated referred specimens include:

*One axis*. **IRSNB M2268**, from the Miocene or earliest Pliocene reworked in a Pliocene basal gravel of either Rumst or Steendorp (Fig. 1D). *Two cervical vertebrae*. **IRSNB M2274**, third cervical vertebra, from the unnamed late middle to early late Miocene layer V at the Antwerp International Airport, Deurne, Antwerp, Belgium. **IRSNB M2270**, seventh cervical vertebra, from the late Miocene Deurne Sands Member of the Diest Formation at the former construction site of the Steenbrug at Borgerhout, Antwerp. *One anterior thoracic vertebra*. **IRSNB M2269**, from the Deurne Sands Member (Diest Formation) at Antwerp. *One middle thoracic vertebrae*. **IRSNB 1075-M245**, from the “Scaldisian” of section 3 at Borgerhout and illustrated by Van Beneden (1877, pl. XV, Figs. 12, 13). *One posterior thoracic vertebra*. **IRSNB M2273**, from the unnamed late middle to early late Miocene layer V at the Antwerp International Airport, Deurne, Antwerp. *One rib*. **IRSNB M2279**, from the “Bolderian” at Kessel, Belgium. *One lumbar vertebra*. **IRSNB 1073-M246**, from the “Scaldisian” of section 3 at Borgerhout and illustrated by Van Beneden (1877, pl. XV, Figs. 15, 16). *Three sacra*. **IRSNB 1092-M236**, from the “Scaldisian” of section 2 at Borgerhout and illustrated as *Phocanella minor* by Van Beneden (1877, pl. XIV, Figs. 18, 19). **IRSNB M2277**, from the “Bolderian” at Kessel. IRSNB VERT-8243-07, from the “Bolderian” at Kessel (biostratigraphy only). *One scapula*. **IRSNB 1068-M241**, right scapula, from the “Scaldisian” of section 3 at Antwerp, illustrated by Van Beneden (1877, pl. XV, Fig. 5). *One humerus*. **IRSNB 1063-M242**, left humerus, from the “Scaldisian” of section 3 at Borgerhout, Belgium, and illustrated by Van Beneden (1877, pl. XV, Figs. 6–9). *One radius*. **IRSNB M2278**, right radius, from the “Bolderian” at Kessel. *One ulna*. **IRSNB M2272**, left ulna, from the unnamed late middle to early late Miocene layer V at the Antwerp International Airport, Deurne, Antwerp, *Four femora*. **IRSNB M2271**, left femur, Miocene in Pliocene basal gravel of Kattendijk Formation at brickyard Swenden, Antwerp. **IRSNB 1049-M247**, right femur, from the “Scaldisian” of section 3 at Borgerhout and illustrated by Van Beneden (1877, pl. XV, Figs. 19–21). **IRSNB 1051-M251**, left femur, from the “Scaldisian” of section 3 at Borgerhout, illustrated by Van Beneden (1877, pl. XV, Figs. 26, 27). **IRSNB 1102-M238**, right femur, from the “Scaldisian” of section 3 at Borgerhout and illustrated as *Phocanella minor* by Van Beneden (1877, pl. XIV, Figs. 21–23).

*Five tibiae*. **IRSNB 1069-M248**, left tibia, from the “Scaldisian” of section 3 at Antwerp and illustrated by Van Beneden (1877, pl. XV, Fig. 22). **IRSNB 1070-M249**, proximal left tibia and fibula, from the “Scaldisian” of section 3 at Borgerhout and illustrated by Van Beneden (1877, pl. XV, Fig. 23, 24). **IRSNB 1090-M233**, proximal right tibia and fibula, from the “Scaldisian” of section 3 at ?Borgerhout, illustrated as *Phocanella pumila* by Van Beneden (1877, pl. XIV, Fig. 12). **IRSNB 1105-M239**, right tibia with proximal fibula, from the “Scaldisian” of section 3 at Borgerhout, illustrated as *Phocanella minor* by Van Beneden (1877, pl. XIV, Figs. 24, 25). **IRSNB 1300-M250**, middle and distal right tibia and middle fibula, from the “Scaldisian” of section 3 at Borgerhout, illustrated by Van Beneden (1877, pl. XV, Fig. 25). *One calcaneum*. **IRSNB M2275**, right calcaneum from the unnamed late middle to early late Miocene layer V at the Antwerp International Airport, Deurne.

##### Comments

In the first description of “*Phoca*” *vitulinoides*, Van Beneden (1871) did not assign any type specimen. The original material included one maxilla, one atlas, one ulna, one sacrum, two calcanea (illustration shows one astragalus), and one phalanx. Originally jointly curated by the Biology and Geology departments at the KUL, this collection was transferred to the IRSNB in different stages during the 20th century. Unfortunately, currently, the original material could neither be located in the collections at the IRSNB nor at the KUL. Primary possibilities for this loss are (1) destruction at the KUL during World War II bombings, and (2) disappearance during transfer from the KUL to the IRSNB. More recently, Koretsky & Ray (2008) re-investigated Van Beneden’s original description, concluding that the type material represents two different species: the illustrated sacrum represents a small species conforming the currently acceptance of “*Ph*.” *vitulinoides* as a small phocine seal, and the other illustrated bones belong to a much larger species. Thorough re-reading of the publications of Van Beneden (1871, 1876, 1877) along with the publications of Koretsky & Ray (2008) and Koretsky & Peters (2008) leads to the conclusion that the latter were mistaken, probably due to incorrect translation of French from the original publication (Van Beneden, 1871). While Van Beneden (1871) states that “*Ph*.” *vitulinoides* does not exceed the size of *Phoca vitulina*, and that the phalanx is similar in size to that of *E. barbatus*, Koretsky & Ray (2008) and Koretsky & Peters (2008) seem to have mistranslated these statements as “*Ph*.” *vitulinoides* being morphologically similar to *Ph. vitulina*, with a size comparable to or even larger than *E. barbatus*. From our study, we consider that all specimens illustrated by Van Beneden (1877) are of comparatively small size for phocine remains, just as other specimens assigned to *N. vitulinoides* [“*Ph*.” *vitulinoides*] and, hence, the illustrated specimens do not appear to belong to different taxa, based on their size alone. However, Koretsky & Ray (2008) were correct when they stated that the original material is unsatisfactory for the designation of a lectotype, with the sacrum being the least unsatisfactory. Because the lectotype designated by Koretsky & Ray (2008) is lost, we replace this lectotype by a neotype: IRSNB M2276a-q (ICZN 75.1). Furthermore, given the quality of the illustrated specimen (Van Beneden, 1871, pl. 1), care should be taken when considering this former lectotype sacrum because no sacrum is known for the geographically close *B. neerlandica*. Despite the strongly elongated wings and the small size (see Description and Comparison), its attribution to *N. vitulinoides* may be questionable. One study by Koretsky & Rahmat (2013) found *B. neerlandica* also being phylogenetically close to *N. vitulinoides*. Apart from *B. neerlandica*, only *Praepusa boeska* is a contemporaneous small phocine seal from the North Sea Basin (Tables S3 and S10) (Koretsky, Peters & Rahmat, 2015). For *Praepusa boeska*, a sacrum has been described, but this specimen had been found isolated and, hence, its assignment to *Praepusa boeska* remains questionable. Given the aforementioned similarities between the lectotype sacrum of *N. vitulinoides*, the neotype sacrum of *N. vitulinoides*, and the only known sacrum of *Praepusa boeska*, it is likely that the sacrum assigned to *Praepusa boeska* actually represents a sacrum of *N. vitulinoides*.

### Description and comparison

#### Cranial skeleton

##### Maxilla

One maxilla has been mentioned by Van Beneden (1871). However, he neither described it in detail nor provided illustrations. As stated earlier, this specimen has been lost and redescription is hence precluded.

#### Axial skeleton

##### Atlas

Van Beneden (1871, pl. 1, Fig. 1) describes and illustrates one atlas that he assigned to “*Phoca*” (*Nanophoca*) *vitulinoides*. This specimen has been lost and will not be treated in the current study. Moreover, in the absence of any other known atlas assigned to the species, it remains uncertain as to whether this atlas indeed belongs to *N. vitulinoides*.

##### Axis

Two axes have been assigned to *N. vitulinoides*: one nearly complete (IRSNB M2268), and IRSNB M2276i (neotype) that only preserves the axial dens (Fig. 3). The right postzygapophysis of IRSNB M2268 is missing and the spinous process is slightly abraded, but the left postzygapophysis is preserved and can be described. The dens is slightly flattened laterally, slightly directed dorsally, and weakly constricted at its base (maximum width 6.1 mm, width at constriction 5.7 mm). In dorsal view, the angle between the dens and the paired articular surfaces for the atlas varies, being strongly obtuse, around 120°, in the two partial axes; and less strongly obtuse in the complete axis. The paired articular surfaces for the atlas are roughly teardrop-shaped. The longitudinal ventral median crest on the body is thin and extends along the entire ventral margin of the axis, broadening posteriorly and forming a pronounced tubercle. There is a slightly elevated and contracted median crest on the dorsal side of the body, on the floor of the neural canal.

The neural canal is slightly dorsoventrally elongate in anterior view, yet not as much as in other phocines. The postzygapophysis is short and stubby, and with a circular postzygapophyseal articular surface facing lateroventrally. The transverse process bifurcates distally, with a relatively long lateral branch and a short, stubby ventral branch. To our knowledge, no other phocine, either extant or extinct, has such a bifurcating transverse process. In *N. vitulinoides*, the spinous process is more strongly anteroposteriorly elongate than in extant Phocidae, extending far posterior to the level of the posterior articular surface of the body of the axis. This character is currently only known for *N. vitulinoides* and the extinct monachine *A. longirostris* (Muizon, 1981). In other extinct and extant phocids, the spinous process is strongly tilted anteroventrally. Potentially analogous to the domestic dog, the combined *musculus obliquus capitis caudalis* has its origin on the lateral side of the spinous process of the axis and inserts on the dorsal wing of the atlas. *Musculus obliquus capitis caudalis* serves to unilaterally rotate the atlas around the dens of the axis and to bilaterally fixate the atlantoaxial joint (for the dog, see Evans & de Lahunta, 2013).

##### Other cervical vertebrae

Only one C3 is preserved (IRSNB M2274) (Fig. 4). Generally among phocines, the spinous process of C3 is consistently smaller than in the following cervical vertebrae, i.e., practically absent (L. Dewaele, 2015, personal observation). Hence, based on the strongly reduced spinous process, IRSNB M2274 is identified as C3. The vertebra is anteroposteriorly shorter than it is dorsoventrally high, compared to the elongated axis. The anterior and posterior articular surfaces of the body are sub rounded to oval and there is a prominent median crest on the ventral side of the body. The dorsoventral length of the neural arch equals that of the body (total height 20.9 mm versus body height 10 mm, Table S1). The prezygapophysis is an anteriorly oriented oval protrusion, i.e., strongly projecting anteriorly but low in dorsal direction. The prezygapophyseal articular surface is oval, and right and left articular surfaces draw an obtuse angle in anterior view. Similarly, the postzygapophyseal articular surface covers the entire ventral part of the simple and robust postzygapophysis, facing ventrally and slightly laterally. The neural canal is strongly reniform and dorsoventrally half as high as the vertebral body, in cross-section (6.4 mm versus 12.1 mm; IRSNB M2270). While other phocines have a tiny spinous process on C3, no spinous process could be detected in the C3 of *N. vitulinoides*.

The isolated C7 IRSNB M2270 shows moderate abrasion of the processes of the neural arch. This specimen is roughly of the same dimensions as the C3 vertebra described above. The vertebra is anteroposteriorly shortened, as compared to extant phocines, and with oval anterior and posterior articular surfaces. A prominent median crest runs along the ventral margin of the body. This median crest is highest in its middle portion and reduced toward the anterior and posterior margins of the body; it is mediolaterally thickest distally. The neural arch is relatively large, with simple prezygapophyses and postzygapophyses, i.e., apparently lacking mammillary processes. Prezygapophyseal and postzygapophyseal articular surfaces are subcircular in outline and cover the entire prezygapophysis and postzygapophysis, respectively. The prezygapophyseal articular surfaces are at a slightly obtuse angle from each another. C7 has transverse foramina, lateral to the vertebral body. The transverse process is spatulate, with a pronounced anterodorsally oriented concavity. The spinous process of C7 is well developed, projecting straight dorsally. C3 and C7 of *N. vitulinoides* do not differ strongly from those of other phocines.

##### Thoracic vertebrae

For the convenience of their description, the thoracic vertebrae are arbitrarily separated in anterior, middle and posterior series (Figs. 5–7). Based on analogy with the known complete series of thoracic vertebrae in extant phocines, in the anterior (T1, T2) and posterior (T11–15) series a single, subcircular costal fovea is located on the lateral side of each centrum. In the middle thoracic vertebrae series (T3–10), each vertebra has crescent-shaped costal foveae on its lateral sides: a cranial and a caudal costal fovea. Also the shape of the transverse processes gradually changes throughout the series of thoracic vertebrae, but it is not interspecifically consistent among phocines (L. Dewaele, 2015, personal observation).

##### Anterior thoracic vertebrae

We know of only two anterior thoracic vertebrae in public collections that can be assigned to *N. vitulinoides*, including IRSNB M2269, and IRSNB 1226-M244b (Fig. 5). The last specimen is associated with a left innominate, but such an association is highly unusual, hence doubtful. Consequently, assignment of anterior thoracic vertebrae is tentative and based on their small size and the overall abundance of bones assigned to *N. vitulinoides* in general (Table S1).

In general, in extant phocines T1 and T2 can easily been distinguished: the costal fovea on the transverse process is strongly concave in T1 and noticeably less concave in T2. In analogy to extant phocines, we tentatively identify IRSNB M2269 and IRSNB 1226-M244b as T1. The body of T1 is short, bearing strongly developed transverse processes and a robust and thick ventral median crest. This median crest is better developed at the cranial part of the vertebra and becomes smaller toward the caudal part of the vertebra. This differs from the cervical vertebra C3, in which the ventral median crest is better developed at the posterior margin of the vertebra, and from C7, in which the ventral median crest is better developed in the center of the ventral margin of the vertebra, as observed in vertebrae of other phocines. The vertebral body is oval to reniform in anterior view. The costal fovea on the vertebral body is strongly concave and well outlined; it faces ventrally and is dorsoventrally at the same level as the long axis through the body of the vertebra.

The transverse process is large compared to the body; the width across the whole vertebra is nearly three times the width of the vertebral body. A similar ratio is also seen in *Pusa* spp., but not in other phocines (L. Dewaele, 2015, personal observation). The prezygapophysis is short and stubby, with right and left circular prezygapophyseal articular surface drawing a slightly obtuse angle with each other, in anterior view. The transverse process is knobby with a small but deep concave transverse costal fovea facing ventrally. The postzygapophysis is not particularly well developed, consisting in a short protrusion with a ventrolaterally facing postzygapophyseal articular facet. The spinous process is transversely thick and robust, and long, compared to other phocines, and strongly projects posteriorly. Compared to other phocines, the neural canal, of reniform section, is very small in relation to the dimensions of the vertebral body: as in C3, the neural canal is dorsoventrally almost half as high as the vertebral body (6.2 mm versus 10.5 mm; IRSNB M2269). Overall, T1 of *N. vitulinoides* does not differ significantly from T1 of other phocines.

##### Middle thoracic vertebrae

The IRSNB houses few middle thoracic vertebrae that can be assigned to *N. vitulinoides*: neotype IRSNB M2276j,k and IRSNB 1075-M245 (Fig. 6). The former have been found in association with a partial skeleton assigned to *N. vitulinoides* and the latter specimen has been found isolated. Hence, assignment of these two middle thoracic vertebrae is only tentative and predominantly based on the small size fitting that of *N. vitulinoides*. Direct designation of a specific position for an isolated middle thoracic vertebra of *N. vitulinoides* as a thoracic vertebra is impossible. While the shape of the transverse process is the most prominently changing structure from T3 to T10, which should primarily be useful for identification, this feature is strongly variable intraspecifically. Hence, it remains difficult to locate an isolated middle thoracic vertebra in the absence of a complete middle thoracic vertebrae series of *N. vitulinoides*. Of lesser value for a precise localization, there is the degree of development of the median ventral crest on the body. This crest is best developed in T3 and lowers progressively backwards.

Currently, only specimens of the middle and posterior sections of the middle thoracic vertebral series are unambiguously assigned to *N. vitulinoides*. Designation of more than ten other, isolated, middle thoracic vertebrae to *N. vitulinoides* remains questionable, due to their strong degree of weathering combined with the lack of any direct association or articulation to other bones. The vertebral body is oval in anterior view and no ventral median crest is observed, contrasting with cervical and anterior thoracic vertebrae. On the anterior part of the lateral surface of the body there is a slightly concave crescent-shaped costal fovea. Based on analogy with extant closely related species, it can be assumed that a similar facet on the posterior part of the lateral surface of the body of the preceding vertebra joins this facet. The prezygapophysis is strongly reduced, basically lying on the neural arch and with the oval articular surface facing dorsally and slightly laterally. Similarly, the postzygapophysis is much reduced and the postzygapophyseal articular surface does not protrude much from the ventral surface of the neural arch. The transverse process is knobby and slightly elongate anteroposteriorly, with a tendency to bifurcate in anterior and a posterior accessory processes. Anteriorly, the transverse process bears a convex costal fovea for the articulation with the tubercle of the rib. The spinous process is short and stubby. As for the thoracic vertebra T1, the neural canal has a reniform section and is small compared to the body of the vertebra, more than in other phocines. At the contact between both halves of the neural arch, at the anterior margin of the arch, there is a small but distinct and sharp process. This process has also been observed in a number of specimens of extant phocine species, including the harp seal, *P. groenlandicus*, and *Pusa* spp., but this is not consistent within each taxon and appears intraspecifically variable. As with T1, the middle thoracic vertebrae of *N. vitulinoides* generally resemble those of other phocines. However, they are on the whole much smaller (Table S1), with a proportionally reduced neural canal.

##### Posterior thoracic vertebrae

Similar to the anterior and middle thoracic vertebrae, we only know a small number of posterior thoracic vertebrae that can be assigned to *N. vitulinoides*: two vertebrae from the neotype (IRSNB M2276l,m) and one specimen from the Antwerp Airport (IRSNB M2273) (Fig. 7). Whereas the body retains the oval to sub rounded outline in anterior view, as observed in the middle thoracic series (see above), it is proportionally longer than in the anterior and middle thoracic vertebrae. The prezygapophysis is strongly developed and it is located dorsal to the body of the vertebra, but protrudes only slightly anterior to it. The articular surface of the prezygapophysis is oval-shaped and facing medially, being nearly parallel to the opposite prezygapophyseal articular surface. The mammillary process of the prezygapophysis extends dorsal to this surface, making a pronounced thick and high tuberosity. The reduced transverse process is a blunt tuberosity projecting lateroposteriorly. The postzygapophysis is similar in size and shape to that in the anterior thoracic vertebrae, and is larger and protrudes more than that in the middle thoracic vertebrae. The postzygapophysis is strongly laterally tilted; its sub rounded articular surface faces laterally. In none of the specimens, the spinous process is completely preserved, preventing proper description. Compared to the posterior thoracic vertebrae of other phocines, the entire vertebral arch is constricted anteroposteriorly, i.e., the prezygapophysis and the postzygapophysis do not extend far anteriorly and posteriorly, respectively. The costal fovea for the articulation of the head of the rib to the body of the vertebra is moderately deep and well outlined. As with the anterior and middle thoracic vertebrae, the neural canal has a reniform section and is small relative to the body of the vertebra when compared to other phocines (neural canal height 6.6 mm versus vertebral body height 13.4 mm). In comparison with *Praepusa vindobonensis*, the anteroposterior length of the posterior thoracic vertebra of *N. vitulinoides* is noticeably shorter.

##### Lumbar vertebrae

Overall, around fifteen lumbar vertebrae have been assigned to *N. vitulinoides* (Fig. 8). However, the majority of them has been found isolated and are only tentatively assigned to that species on the basis of size. Only six lumbar vertebrae are associated with other remains assigned to *N. vitulinoides*: IRSNB M2276n,o,p (three vertebrae) and IRSNB 1059-M240d-f (three vertebrae; Fig. 8). Lumbar vertebra IRSNB 1092-M236 had originally been assigned to *Phocanella minor*, but appears to belong to *N. vitulinoides* based on our observations.

The ventral median crest on the vertebral body is slightly more robust and larger posteriorly. Hence, the anterior articular surface is subcircular, while the posterior articular surface is somewhat more triangular. The lumbar vertebrae of *N. vitulinoides* are relatively more elongate anteroposteriorly than the cervical and thoracic vertebrae in extant phocids. The prezygapophysis is well developed and projects only slightly anteriorly, but strongly dorsally. Half of the articular surface on the prezygapophysis lies at a level anterior to the anterior articular surface of the body. This surface is oval and the prezygapophysis has a strongly developed, dorsally located mammillary process. The angle between the two opposing prezygapophyseal articular surfaces, in anterior view, is less than 90°. The postzygapophysis is well developed and the circular postzygapophyseal articular surface faces ventrolaterally. The spinous process does not extend dorsally beyond the level of the prezygapophysis.

In none of the specimens the transverse process is preserved completely; nevertheless, the preserved parts indicate a strongly anteriorly projected transverse process. This anterior projection is seemingly stronger than in most other phocines, except *Pusa* spp. In *N. vitulinoides*, the lumbar transverse process is also relatively thin, anteroposteriorly. Among extant phocines, a similarly thin transverse process in only observed in *Pusa* spp. On the posterolateral margin of the vertebral body, between the base of the transverse process and the base of the prezygapophysis, there is a small and blunt, but prominent accessory process (sensu Evans & de Lahunta, 2013). The same process is present in other phocines, but it is commonly not as pronounced as in *N. vitulinoides*. The neural canal is reniform in cross-section. Lumbar vertebrae of other phocines have a proportionally larger neural canal.

##### Sacrum

The sacrum figured by Van Beneden (1871; pl. 1, Fig. 2) represented the former lectotype specimen of *N. vitulinoides* (Fig. 9). Although this sacrum has been lost, its illustration shows a combination of characteristics diagnostic of *N. vitulinoides* among Phocinae: large wings (total lateral width across wings three times the width of the promontory), slightly everted anteriorly, reniform sacral foramina, and small size. However, formal identification as *N. vitulinoides* based on these characters is not straightforward: the stratigraphic context of this specimen is very poorly resolved (Miocene–Pliocene) and currently no sacrum is known for the potentially sympatric *B. neerlandica*. Twelve specimens unambiguously assignable to *N. vitulinoides* have been identified at the IRSNB, including the sacrum of the neotype IRSNB M2276a and two other referred specimens IRSNB M2277 and IRSNB VERT-8243-07. A sacrum originally assigned to *Phocanella minor* by Van Beneden (1877, IRSNB 1092-M236) was reassigned to *N. vitulinoides*: this particular sacrum is of both size and shape markedly similar to that of other sacra of *N. vitulinoides*. The number of fused sacral vertebrae in the sacrum is one of the primary characters separating extant monachines and phocines: monachines have three fused sacral vertebrae, while phocines have four fused sacral vertebrae (Muizon, 1981). Interestingly, *N. vitulinoides* yields specimens of sacra with either three (nine specimens observed, including IRSNB M2276a (neotype), IRSNB M2277, and IRSNB VERT-8243-07) or four vertebrae (three specimens observed, including IRSNB 1092-M236, IRSNB 1059-M240). This is surprising, as noticeable intraspecific variation in the number of fused sacral vertebrae in pinnipeds is not known to us. It is possible that the fourth sacral vertebra did not fuse during growth in a significant number of individuals. Given the limited number of specimens adequately preserved, it is impossible to ascertain whether *N. vitulinoides* predominantly had three or four fused sacral vertebrae.

The wings for the articulation with the innominate are large relative to the anterior articular surface of the first sacral vertebra and their lateral projection strongly bends anteriorly. Overall, these wings are much larger in phocids than they are in other carnivorans (Muizon, 1981). The ratio of width across the wings/width across the promontory in *N. vitulinoides* is around 3 (Dewaele, Lambert & Louwye, 2017). Also, the ventral margins of the wings extend far ventral to the ventral border of the first sacral vertebra. In other phocines, these sacral wings do not tend to project as far anteriorly as in *N. vitulinoides*, whereas in monachines there is not such a strong ventral deflection of the sacral wings. The lateral margin of the sacral wings of *N. vitulinoides* is directed dorsolaterally. This is a typical phocine trait, contrasting with the dorsoventrally directed margin in monachines. The prezygapophysis of the first sacral vertebra is raised above the dorsal margins of the wing, but retains a thick base on the wing. The prezygapophyseal articular surfaces are roughly teardrop-shaped and right and left surfaces form an approximate 90° angle with each other. The lateral sacral crest is relatively thick dorsoventrally at the fused vertebral bodies and more blade-like laterally. This blade-like crest is located lateral to the third and fourth sacral vertebrae, while the part of the lateral crest located along the second sacral vertebra is robust, forming a thick and robust bridge around the first sacral foramen. Posteriorly, this crest becomes a blunt tuberous extension posterior to the fourth sacral vertebra. The sacral canal is dorsoventrally flattened, reniform in anterior view, and forming a narrow crescent posteriorly. The sacral foramina vary intraspecifically from a reniform to an hourglass shape. A thick and broad bridge covers the first sacral foramen laterally. The sacral spinous processes are all fused and their dorsal apices align to form a single median sacral crest. Anteriorly, this median sacral crest is dorsally high, and markedly lowering posteriorly. At the level of S2 and S3, this crest is laterally flattened; at the level of S4 (when present), it forms a blunt posteriorly projecting stub. In anterior view, the crest is thicker at the center of each individual spinous process and thinner at the fused margin between two spinous processes. The intermediate sacral crests are knob-like, but anteroposteriorly elongate, being thicker anteriorly, and tapering posteriorly.

The sacrum of the contemporaneous small *Praepusa boeska* from the Netherlands has been described and illustrated by Koretsky, Peters & Rahmat (2015), based on an isolated and strongly damaged specimen. It shows marked similarities with that of *N. vitulinoides*: three fused sacral vertebrae, relatively large sacral wings, and a generally small size. Hence, it is not impossible that this sacrum assigned to *P. boeska* instead represents that of *N. vitulinoides*.

##### Caudal vertebrae

**Figure 10:**
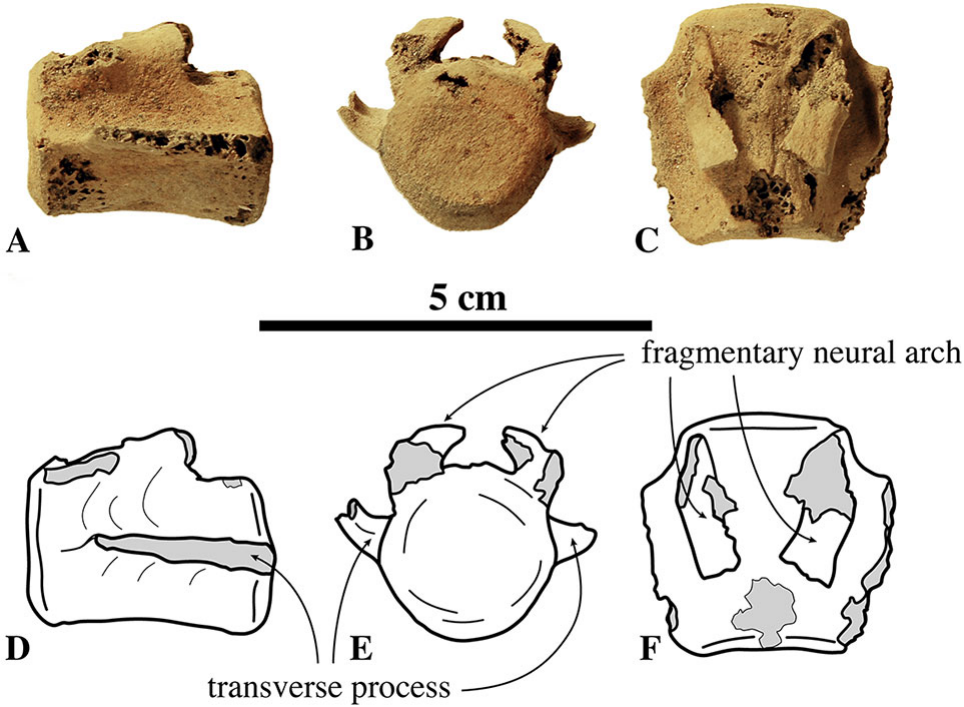
Caudal vertebra of *Nanophoca vitulinoides*. IRSNB M2276q (neotype) caudal vertebra of *Nanophoca vitulinoides*? (A–C) and corresponding drawings (D–F) in left lateral (A, D), anterior (B, D), and dorsal (C, F) view. Broken or obliterated areas are indicated in gray.

Currently, only one incomplete caudal vertebra (neotype, IRSNB M2276q) has been tentatively assigned to *N. vitulinoides* (Fig. 10). Based on its size as compared to the sacrum and on the degree of development of the transverse processes and vertebral arches, this vertebra is proposed to be the first caudal vertebra. However, it is significantly larger than S3, the last sacral vertebra of this specimen, raising doubts about the association with the other bones of this specimen, and hence about the determination of this caudal. Although the dimensions of the body seem similar to those of the lumbar vertebrae of the same specimen IRSNB M2276n,o,p (neotype), the anteroposteriorly elongate shape of the preserved portion of the transverse process prevents from considering this caudal vertebra a lumbar vertebra.

##### Rib

**Figure 11:**
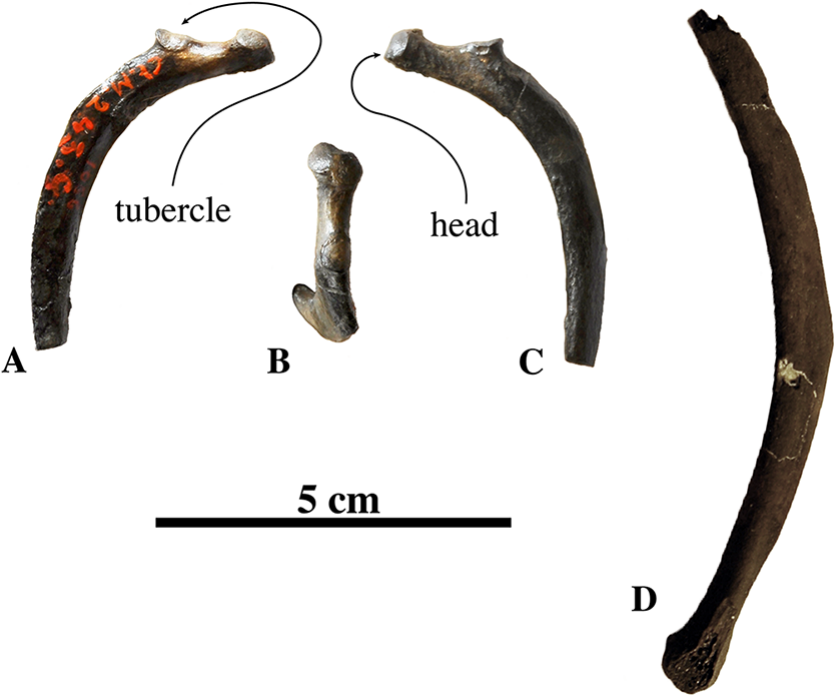
Ribs of *Nanophoca vitulinoides*. IRSNB M1066-M243c, right rib of *Nanophoca vitulinoides* in anterior (A), dorsal (B), and posterior (C) view. IRSNB M2279, rib of *Nanophoca vitulinoides* (D).

Only two ribs may be assigned to the species: IRSNB 1066-M243c (associated with radius and ulna) and IRSNB M2279 (found in association with radius IRSNB M2278) (Fig. 11). Both ribs are incomplete, with the former missing its distal half and the latter missing its proximal extremity.

Overall, the ribs of *N. vitulinoides* are relatively slender and strongly flattened anteroposteriorly. Flattening is most prominent proximally and tends be strongest pronounced at the medial margin of the rib, yielding a teardrop-shaped section of the rib. The head of IRSNB 1066-M243c is seated on a long neck, forming a knob-like proximal extremity. The articular surface on the head of the rib is slightly elongated anteroposteriorly. The tubercle is little pronounced, forming only a weak protuberance at the base of the neck. The tubercle is entirely covered by a convex articular surface for articulation with the corresponding costal fovea on the transverse process of the vertebra. At its distal extremity, IRSNB M2279 thickens slightly radially, forming a pronounced knob-like extremity.

#### Appendicular skeleton

##### Scapula

**Figure 12:**
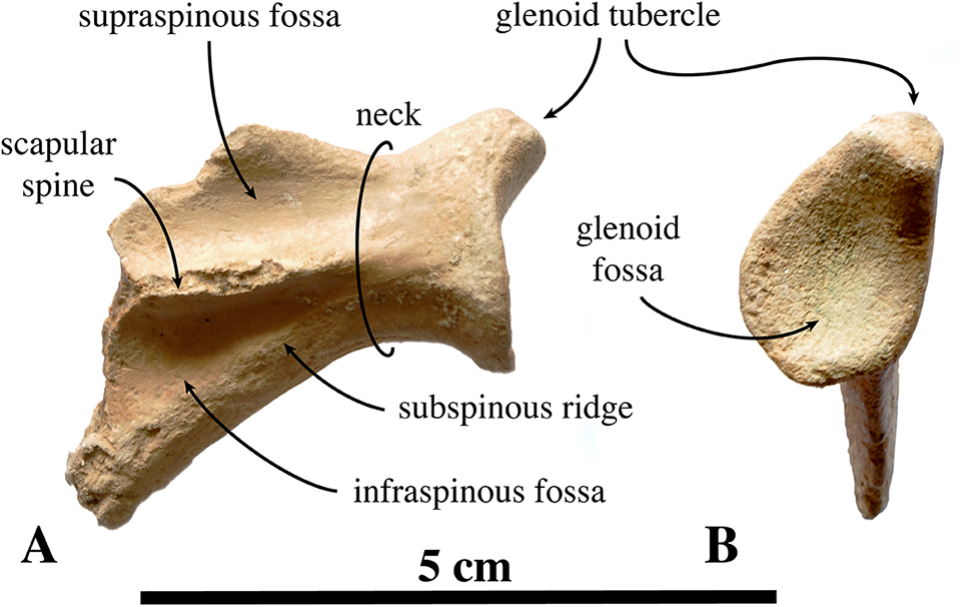
Scapula of *Nanophoca vitulinoides*. IRSNB M2276f (neotype), partial right scapula of *Nanophoca vitulinoides* in lateral (A) and anterior (B) view.

Two scapulae are assigned to *N. vitulinoides*: a right scapula from the partial skeleton (neotype, IRSNB M2276f) and one isolated scapulae IRSNB 1068-M241 (Van Beneden, 1877, pl. XV, Fig. 5) (Fig. 12). Only the head and neck of the scapula are known. The scapula is similar in size to that of *Praepusa vindobonensis*. In cranial view, the glenoid fossa is roughly teardrop-shaped and slightly concave, with the glenoid tubercle at its apex. The edges of the glenoid fossa are straight in lateral view, with a craniodorsally projecting glenoid tubercle. This trait is also observed in *H. grypus*, *Phoca* spp., *Praepusa vindobonensis*, and *Pusa* spp., providing an enlarged origin for *musculus biceps* (Toula, 1897; Howell, 1929; Bryden, 1971; Muizon, 1981). In lateral view, the glenoid tubercle is square. The surface for the origin of *musculus triceps brachii* on the ventrolateral margin of neck of the scapular spine is well defined and relatively deep. Muizon (1981) identified this surface as being the origin of the long head of *musculus triceps brachii* (for *P. pacifica* and *A. longirostris*), while Howell (1929) identified it to be the origin of the lateral head of *musculus triceps brachii* (for *Pusa hispida*). At the scapular neck, the scapular spine appears to fuse with an infraspinous ridge (=secondary spine in Tedford (1976)) lying ventral to the scapular spine. Hodgetts (1999) refers to this ridge and the scapular spine as “two ridges of bone.” Savage (1957) identified this condition as being present in the extinct mustelid *Potamotherium* Geoffroy, 1860, referring to this feature as “secondary spine,” and Tedford (1976) observed a similar condition in the extant *Phoca vitulina* and *Pusa* spp. However, we only observed a lowly raised ridge in Phocidae and consider it a “ridge” rather than a “spine,” which is only noticeably developed in the anterior portion of the scapula. Moreover, referring to this structure as the infraspinous ridge avoids confusion with the secondary scapular spine observed in the middle of the supraspinous fossa of otariids (Berta & Wyss, 1994). The infraspinous ridge separates the origins of *musculus infraspinatus* (dorsal) and *musculus teres major* (ventral), both rotator muscles of the humerus.

##### Humerus

**Figure 13:**
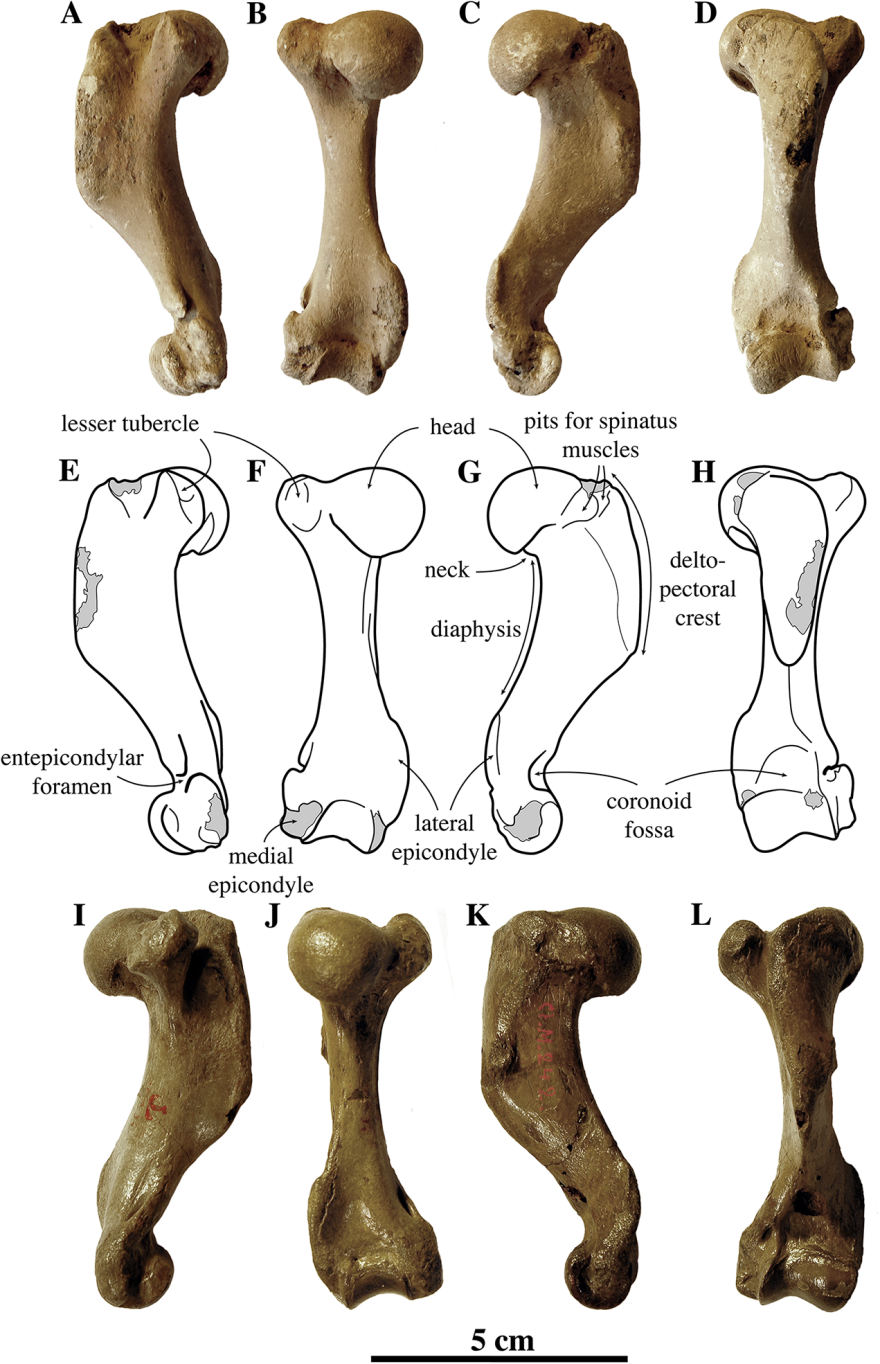
Humeri of *Nanophoca vitulinoides*. IRSNB M2276c (neotype) left humerus of *Nanophoca vitulinoides* (A–D) and corresponding drawings (E–H) in medial (A, E), anterior (B, F), lateral (C, G), and posterior (D, H) view. IRSNB 1063-M242 right humerus of *Nanophoca vitulinoides* in medial (I), anterior (J), lateral (K), and posterior (L) view. Broken or obliterated areas are indicated in gray.

**Figure 14:**
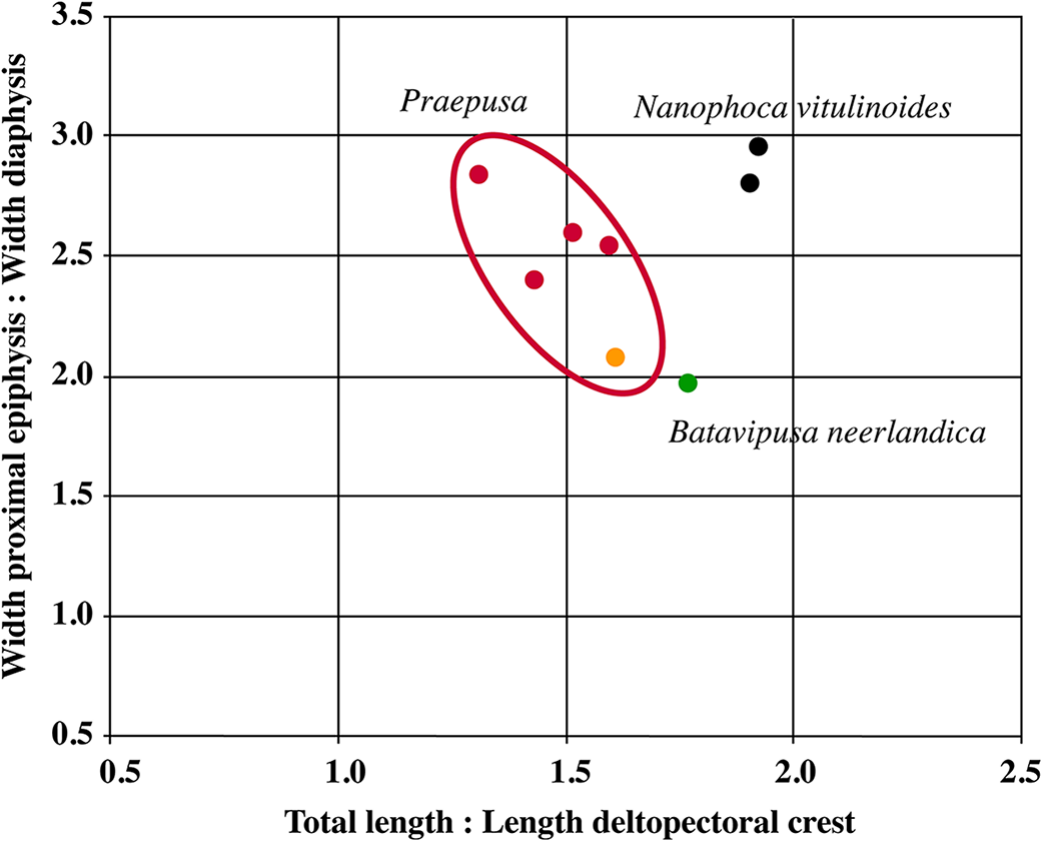
Scatterplot of humerus measurements of contemporaneous small Phocinae from the Neogene North Sea and Paratethys basins. Scatterplot based on the humerus measurements shown in Table S10. Green represents *Batavipusa neerlandica*, black *Nanophoca vitulinoides*, orange *Praepusa boeksa*, and red *Praepusa vindobonensis*. The red ellips encompasses both *Praepusa* species. For the selected characters, the humerus of *N. vitulinoides* differs noticeably from that of the other considered taxa.

**Figure 15:**
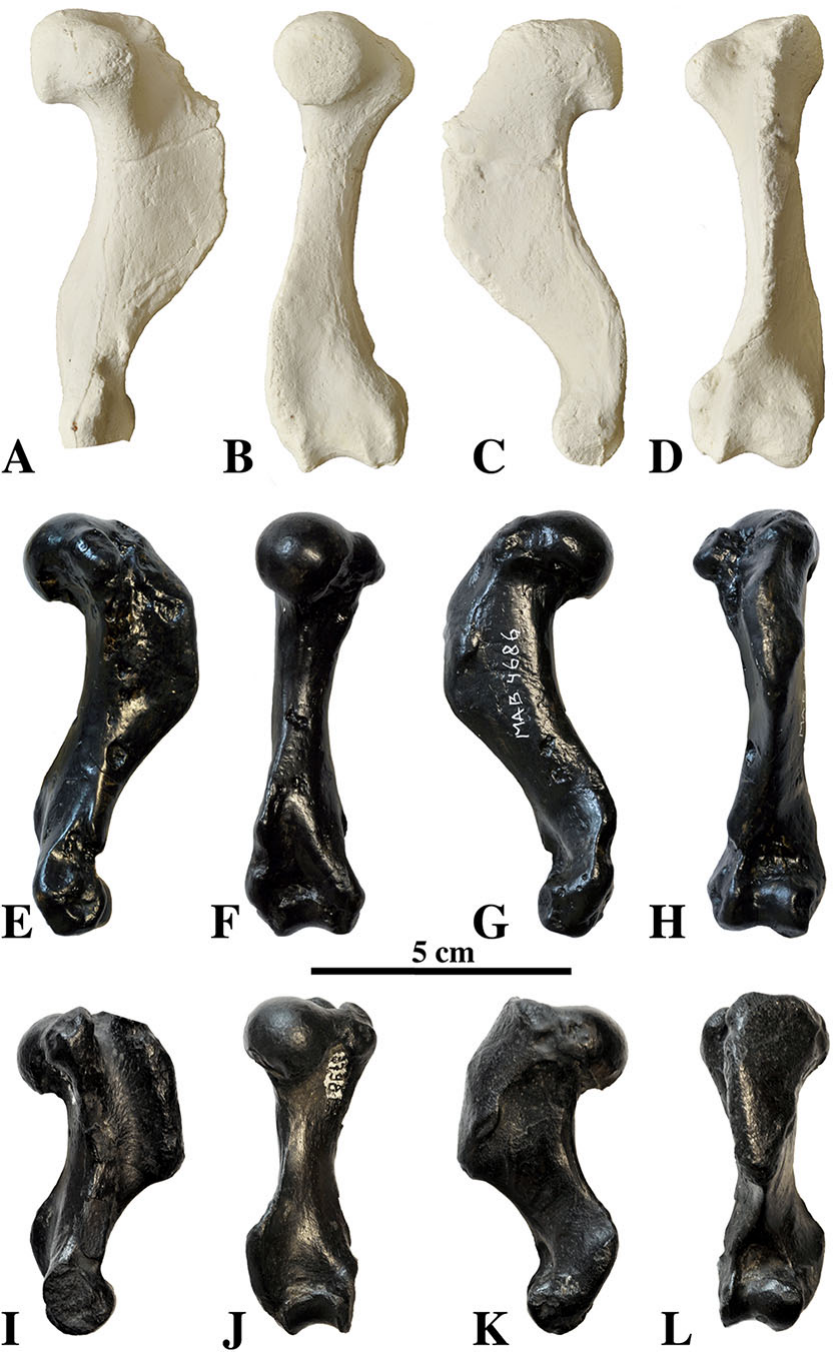
Comparison of humeri. Humeri of *Praepusa vindobonensis* (A–D) (cast), *Praepusa boeska* (E–H) (holotype MAB 4686), and *Batavipusa neerlandica* (I–L) (holotype MAB 3789) for comparison with the humerus of *Nanophoca vitulinoides*, shown in Fig. 13. Humeri in medial (A, E, I), posterior (B, F, J), lateral (C, G, K), and anterior view (D, H, L). Note the relatively small size of the humerus of *Batavipusa neerlandica*.

**Figure 16:**
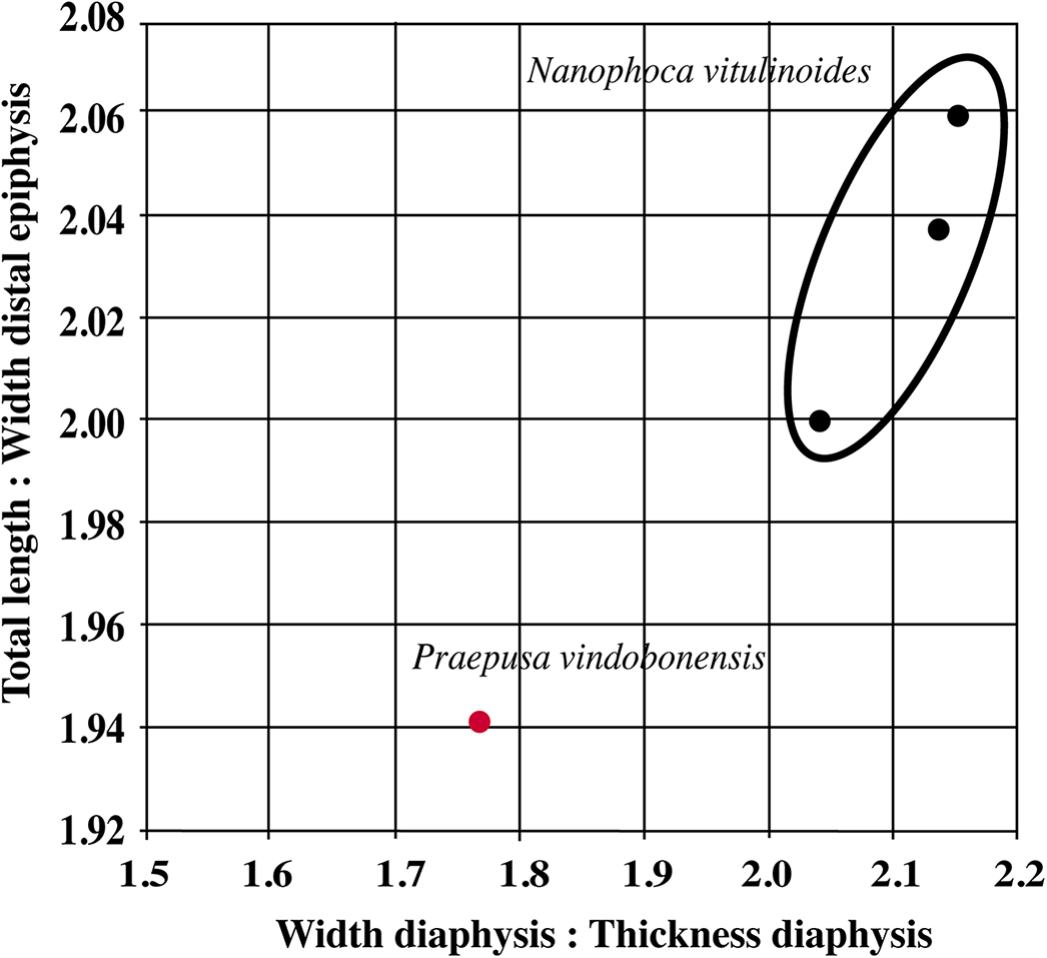
Scatterplot of femur measurements of contemporaneous small Phocinae from the Neogene North Sea and Paratethys basins. Scatterplot based on the femur measurements shown in Table S11. Black represents *Nanophoca vitulinoides*, and red the average of over 20 *Praepusa vindobonensis* specimens. The black ellips encompasses all *N. vitulinoides* measurements. For the selected characters, the femur of *N. vitulinoides* differs noticeably from that of the average of *P. vindobonensis* specimens.

Humeri of *N. vitulinoides* are some of the most frequently collected phocid remains from the Neogene of the Antwerp area (Fig. 13). The collection at the IRSNB yields over 40 small phocine humeri of varying degrees of preservation and completeness. Unfortunately, only about 20 of these specimens could be attributed to *N. vitulinoides* unambiguously. The poor state of preservation of other specimens inhibited unambiguous attribution to the species, due to the absence or abrasion of diagnostic regions. Specimens used for the description in this study include the right humerus of the neotype (IRSNB M2276c) and the specimen illustrated by Van Beneden (1877, IRSNB 1063-M242). The humerus is one of the most commonly found specimens of extinct seals in general (L. Dewaele, 2015, personal observation), and it should be noted that the humerus of *N. vitulinoides* can easily be compared with contemporaneous extinct seals from the North Sea Basin and Paratethys. Given the small size of *N. vitulinoides*, only a limited number of other taxa should be considered for close comparison: *B. neerlandica* and *Praepusa boeska* from the Netherlands, and *Praepusa vindobonensis* from the Paratethys. A scatterplot of a quantitative analysis of the humerus shows that *N. vitulinoides* is morphologically distinct from the other three species (Figs. 14 and 15; Table S10). Similar comparisons are more difficult for the femur given the very poor preservation of the femur of *B. neerlandica* (Koretsky & Peters, 2008), and the lack of femora in the fossil record of *Praepusa boeska* (Koretsky, Peters & Rahmat, 2015). However, a preliminary comparison between three femora of *N. vitulinoides* and the averaged femur of *Praepusa vindobonensis* shows that both are morphologically distinct (Fig. 16; Table S11).

The head of the humerus is roughly hemispherical and strongly overhangs the diaphysis posteriorly. Related to this projection, the area of the diaphysis just distal to the neck is correspondingly well-excavated, indicating relatively strong development of the lateral head of the *musculus triceps brachii*. A similarly strongly overhanging head has been observed in the extinct *Praepusa vindobonensis* and extant *Pusa*, but not in *Praepusa magyaricus*. In posterior view, the proximal tip of the head of the humerus reaches slightly more proximal than both the lesser and the greater tubercles. This contrasts with most extant phocids, in which the lesser tubercle is much more prominent and reaches a much more proximal level than both the head and the greater tubercle. Among extant seals, only the genus *Monachus* has a reduced lesser trochanter, whereas this character is more common in extinct phocids, for example the monachines *A. longirostris*, *Monotherium* spp. and *P. pacifica*, and the phocines *B. neerlandica*, *L. proxima* [*Leptophoca lenis*], *Pachyphoca* spp. Koretsky & Rahmat, 2013, and *Praepusa* spp. (Muizon, 1981; Koretsky, 2001; Koretsky & Peters, 2008; Koretsky & Rahmat, 2013; Koretsky, Peters & Rahmat, 2015; Dewaele, Lambert & Louwye, 2017). An enlarged greater tubercle, exceeding the height of the head proximally, has been observed in some extinct species, such as *B. neerlandica*, *Praepusa magyaricus*, and *Praepusa vindobonensis* but not in extant species. However, in all species of Phocidae, the greater tubercle is still significantly smaller than in Otariidae (L. Dewaele, 2015, personal observation). The short lesser tubercle strongly deviates from the long axis of the bone and the base of this deviation is located very proximally on the diaphysis, similar to *Praepusa vindobonensis* and other extinct phocines, but contrasting to extant phocines in which the deviation of the lesser tubercle off the diaphysis starts more distal on the bone. A proximodistally elongated rugose surface on the posterior surface of the lesser tubercle is the attachment site of the *musculus subscapularis*. The bicipital groove is U-shaped, deep, and narrow. In transverse section, the deltopectoral crest is thick, only slightly thinner than the diaphysis. The deltopectoral crest extends along the proximal half of the bone. In lateral view, the deltopectoral crest of *N. vitulinoides* is equally wide throughout, to slightly wider proximally. This contrasts with the contemporary *Praepusa vindobonensis* and *Praepusa magyaricus*, having a deltopectoral crest that is wider distally. In anterior view, the deltopectoral crest of *N. vitulinoides* is wider than that of *Praepusa vindobonensis* and *Praepusa magyaricus*, which have a relatively slender deltopectoral crest. At the proximolateral surface of the deltopectoral crest, the insertion facets for *musculus supraspinatus* and *musculus infraspinatus* are well defined, deep pits, with the pit for the *musculus supraspinatus* being the deepest and the pit for the *musculus infraspinatus* being smaller and just anteroproximal to the former. The deltopectoral crest is strongly elongated and smooth, with a marked deltoid tuberosity halfway on its lateral side. This tuberosity serves as an insertion site for *musculus deltoideus* (Howell, 1929; Muizon, 1981) or *musculus humerotrapezius* (Howell, 1929). Distally, the deltopectoral crest of *N. vitulinoides* ends more abruptly than it does in *Praepusa vindobonensis*, but not as abruptly as in *B. neerlandica*.

The diaphysis is only weakly curved in lateral view compared to most extant phocines, except the hooded seal *C. cristata* and the ribbon seal *H. fasciata*. Among extinct phocines, the geologically oldest stem phocine *L. proxima* has a relatively straight diaphysis as well (True, 1906; Koretsky, 2001; Dewaele, Lambert & Louwye, 2017). *Praepusa vindobonensis* and *Praepusa magyaricus* have relatively straight humeri, whereas the humerus of *B. neerlandica* is strongly curving. In lateral view, the diaphysis appears more slender compared to the size of the epiphyses than it is in all extant phocines, except *Phoca* and *Pusa* spp. A slender diaphysis has also been observed in the extinct phocines *L. proxima*, *Praepusa vindobonensis*, *Praepusa magyaricus* and *S. sintsovi*. The diaphysis of the humerus has a sharp edge located distal to the head of the humerus, continuing distally toward the lateral epicondyle. This edge separates the areas of origin for the *musculus triceps brachii caput medialis* posteriorly and the *musculus brachialis* laterally and has been observed in *B. neerlandica* (Koretsky & Peters, 2008), but not in other extinct and extant phocines. At the distal epiphysis, the lateral epicondyle reaches twice as far proximally as the medial epicondyle, but still ends distal to the distal end of the deltoid crest. The crest of the lateral epicondyle is thick and positioned proximally. Consequently, the attachment surface for *musculus supinator* is well developed in *N. vitulinoides*. The medial epicondyle is very robust and square, projecting well laterally, but reaching only slightly more proximal than the trochlea. The entepicondylar foramen is oval. The lateral epicondyle does not reach proximally the level of the deltopectoral crest, as it does in *Praepusa* species. In anterior view, the middle portion of the humeral trochlea is at the level of the coronoid fossa, a diagnostic trait distinguishing phocines from monachines (Koretsky, 2001). The coronoid fossa on the anterior surface of the humerus is well defined, with a semicircular outline, terminating proximally at a level intermediate to the lateral and medial epicondyles. The olecranon fossa is a very small, only about 2 mm in height and 5 mm in width, but forms a deep pit just proximal of the trochlea. Overall, the humerus of *N. vitulinoides* shares most similarities with the extinct *Praepusa vindobonensis*.

##### Ulna

**Figure 17:**
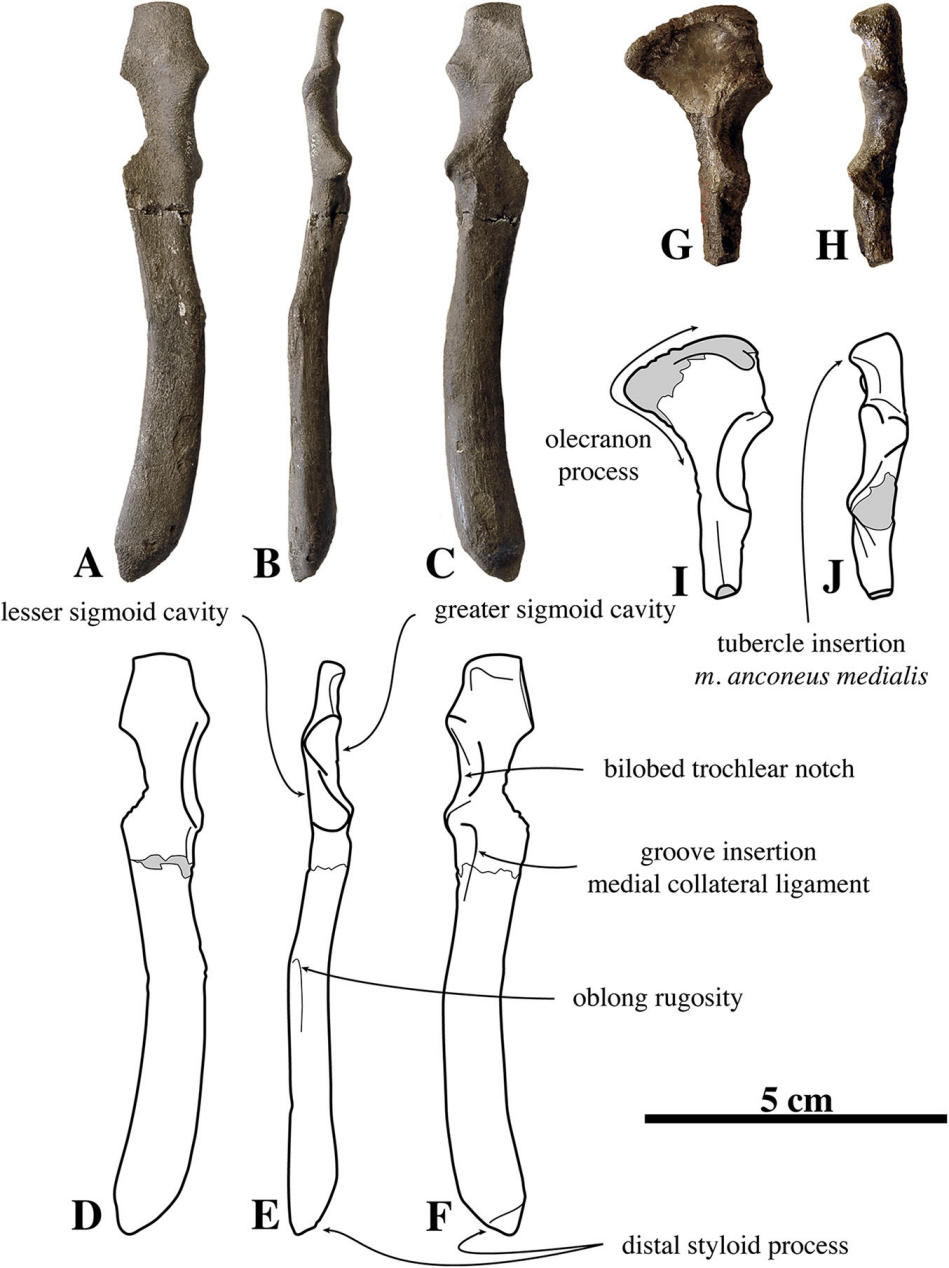
Ulnae of *Nanophoca vitulinoides*. IRSNB M2272 left ulna of *Nanophoca vitulinoides* (A–C) and corresponding drawings (D–F) in medial (A, D), anterior (B, E), and lateral (C, F) view. And IRSNB 1066-M243b right ulna of *Nanophoca vitulinoides* (G, H) and corresponding drawings (I, J) in lateral (G, I) and anterior (H, J) view. Broken or obliterated areas are indicated in gray.

Currently one right and one left ulnae have been assigned to *N. vitulinoides*: IRSNB 1066-M243b and IRSNB M2272. IRSNB 1066-M243b is associated with a partial right radius (IRNSB 1066-M243a) and a partial rib (IRNSB 1066-M243c) (Fig. 17). The ulna is of very small size, as the other remains of *N. vitulinoides*, and slender, as in *Pusa* and *Phoca* spp. The trochlear notch is bilobed, as in other phocines (except *C. cristata* and *P. groelandicus*, which have three lobes). The greater sigmoid cavity (proximal lobe) is elongate and narrow, and almost entirely restricted to the anterolateral side of the ulna. The lesser sigmoid cavity (distal lobe) is clearly defined and triangular, located along the anterior margin of the ulna. Compared to other phocines, the trochlear notch is strongly concave in lateral view. The proximal margin of the olecranon process slopes only slightly posterodistally in *N. vitulinoides*, contrasting with other Phocinae, in which the olecranon process slopes much more abruptly. The tubercle for the insertion of the *musculus anconeus medialis* on the anteromedial side of the olecranon process is well developed compared to extant phocines. The olecranon process is proportionally large compared to the width of the diaphysis, a condition approached by *Phoca*, and *Pusa*. However, the diaphysis is slender in *Phoca*, *N. vitulinoides*, and *Pusa*, which might explain the apparent relatively large size of the olecranon process in those taxa. The anconeal process is prominent, which does not appear to be significantly different from that of *Phoca vitulina*, and *Pusa*. A well-developed anconeal process is also present on the partial ulna figured by Van Beneden (1871, pl. 1, Fig. 5). Unfortunately, this specimen has been lost and could only be examined from illustrations; hence, its assignment to *N. vitulinoides* can only be considered tentative on the basis of the development of the anconeal process. On the medial side of the diaphysis, there is a marked, narrow and proximodistally elongate groove just distal to the trochlear notch. This groove most likely served for the insertion of the medial collateral ligament of the elbow joint. At midlength of the diaphysis, an oblong rugosity indicates the insertion of the interosseous ligament uniting radius and ulna. The distal styloid process is pointed. The ulna of *N. vitulinoides* differs strongly from that of its close relative *Praepusa vindobonensis*. The latter has a relatively strongly sloping olecranon process which is not particularly large, compared to extant Phocinae. The ulna of *Praepusa vindobonensis* also has a relatively thick and straight diaphysis. All this contrasts with *N. vitulinoides*.

##### Radius

**Figure 18:**
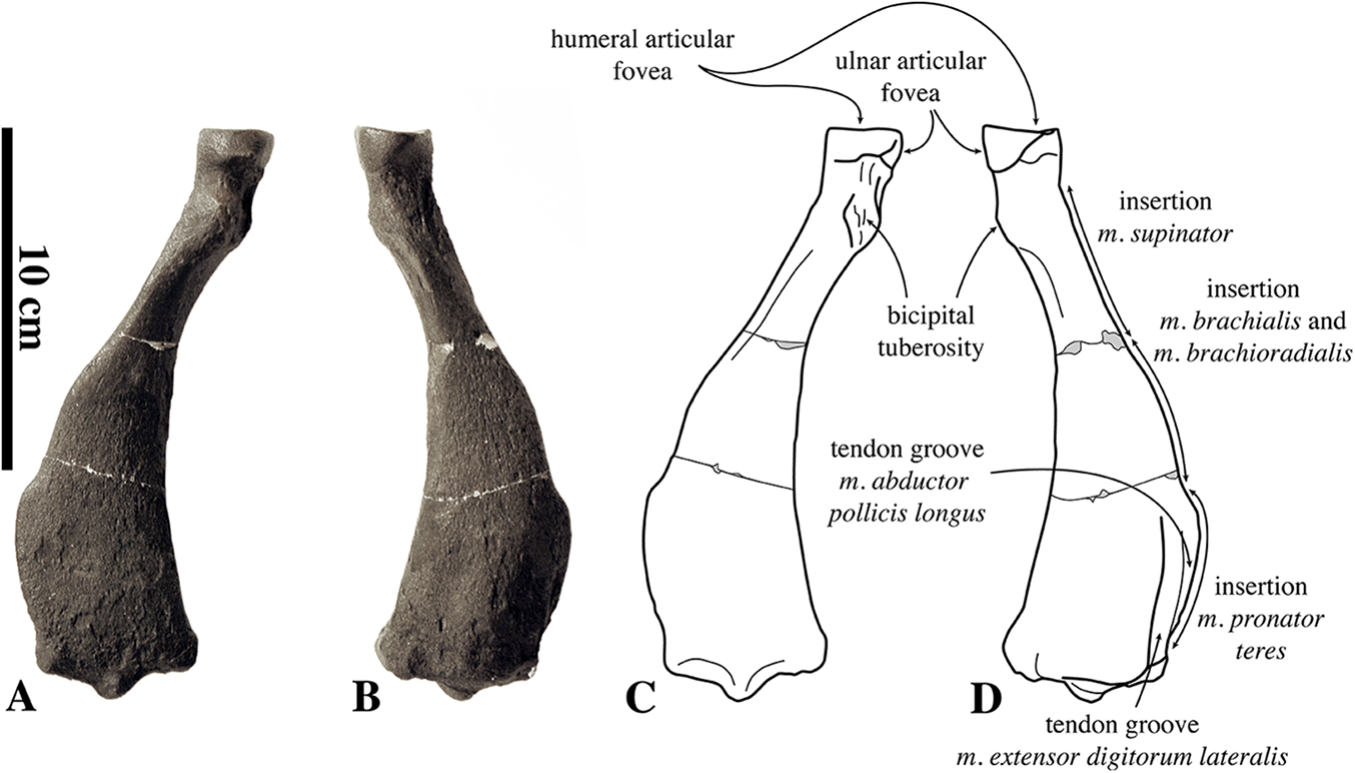
Radius of *Nanophoca vitulinoides*. IRSNB M2278 right radius of *Nanophoca vitulinoides* (A, B) and corresponding drawings (C, D) in medial (A, C) and lateral (B, D) view.

Two radii have been assigned to *N. vitulinoides* (Fig. 18). One, IRSNB M2278, is complete and associated with a partial rib (IRSNB M2279), whereas only the proximal part of the other radius (IRSNB 1066-M243a), associated with a partial rib and a partial ulna (IRSNB 1066-M243c), is preserved. Overall, the radius is small and relatively slender, relatively barely widening toward the distal end, as in the extant *Phoca* spp. and *Pusa* spp., and the extinct *L. proxima* and *Praepusa vindobonensis*. The proximal epiphysis has a slightly mediolaterally elongated humeral articular fovea. The articular fovea for the ulna is moderately developed; yet, it strongly protrudes medially off the articular fovea for the humerus. The bicipital tuberosity is strongly pronounced and is located on the posteromedial side of the shaft. This tuberosity is located relatively proximal on the shaft, nearly bordering the ulnar fovea of the proximal epiphysis. Directly distal to the bicipital tuberosity, the diaphysis takes on a rather strong cranially convex shape, which is a phocine trait (Muizon, 1981); it is yet stronger in *N. vitulinoides* than in extant phocines. The surface for *musculus supinator* on the mediolateral face of the shaft is well excavated. The eminence for the attachment of the *musculus brachialis* and *musculus brachioradialis* is well developed and located proximal on the radius when compared to other phocines. The strong development of the eminence for the attachment of *musculus brachioradialis* suggests relatively strong development of this muscle. The surface for the attachment of the *musculus pronator teres* is also moderately well developed. Although the distal part of the radius is moderately abraded, it can be observed that the grooves for the tendons of *musculus extensor digitorum lateralis* and *musculus abductor pollicis longus* are wider than deep, thus only weakly to moderately developed. The radius of *N. vitulinoides* most strongly resembles the radius of *Pusa* spp. On the other, the radius assigned to *Praepusa vindobonensis* by Toula (1897) differs strongly from the one of *N. vitulinoides*. The radius of *Praepusa vindobonensis* is much more straight and it appears that the development of the insertion areas for *musculus supinator*, *musculus pronator teres*, and *musculus brachioradialis* approaches much more the condition of the extant Lobodontini (see Muizon, 1981).

##### Innominate

**Figure 19:**
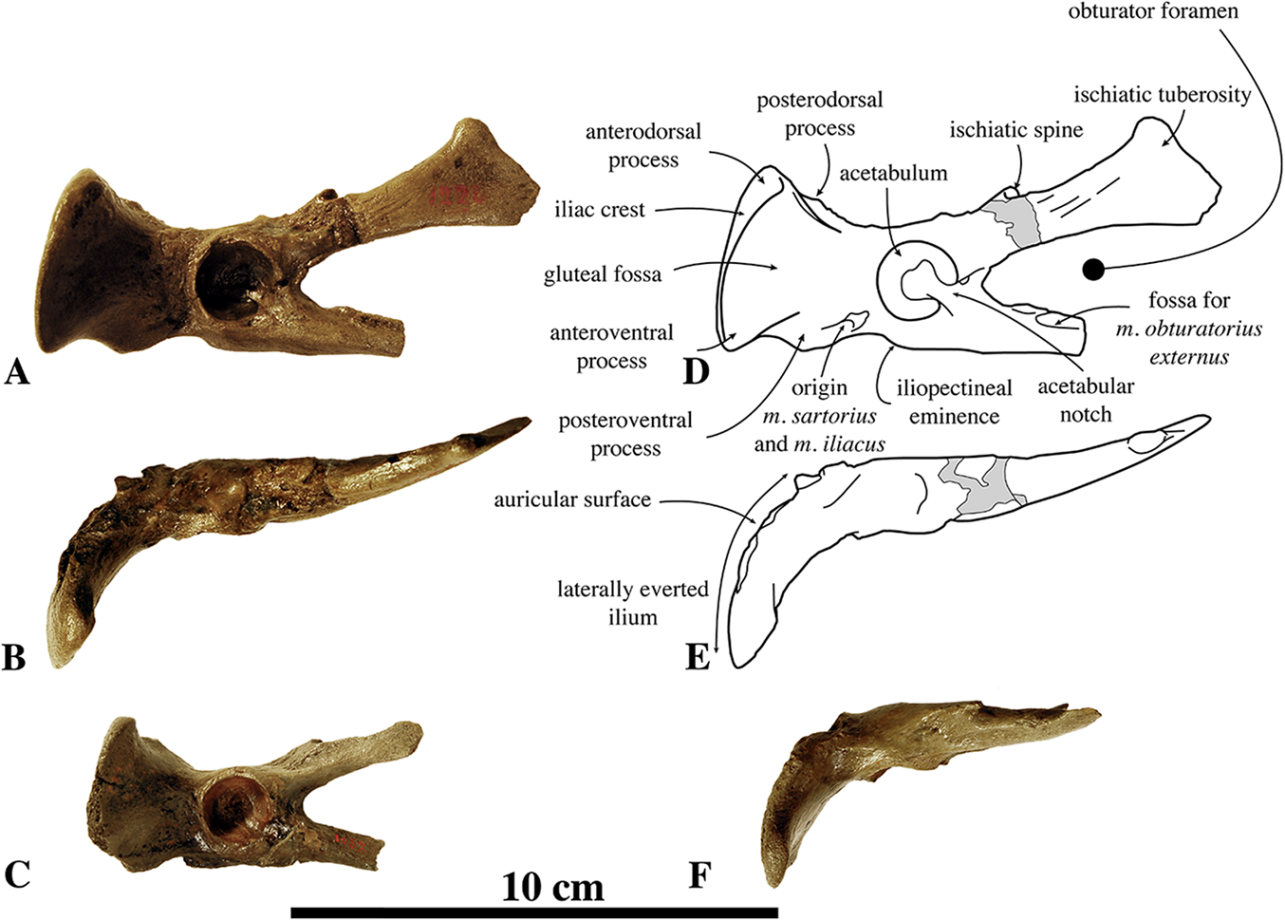
Innominates of *Nanophoca vitulinoides*. IRSNB 1226-M244a left innominate of *Nanophoca vitulinoides* (A, B) and corresponding drawings (D, E), in lateral (A, D) and dorsal (B, E) view. IRSNB 1059-M240a left innominate of *Nanophoca vitulinoides* in lateral (C) and dorsal (F) view. Broken or obliterated areas are indicated in gray.

The innominate of *N. vitulinoides* is one of the most completely known fossil phocid innominates from the Antwerp area (Fig. 19). Seven innominates from the IRSNB can be assigned to *N. vitulinoides* unambiguously. Furthermore, nearly twenty other innominates may tentatively be assigned to the species. The latter specimens are within the size range of *N. vitulinoides*, but they are all less well preserved. In none of the specimens the ischium and the pubis are completely preserved. The ischiatic tuberosity is preserved in only two innominates (IRSNB 1059-M240a and IRSNB 1226-M244a). The Phocinae are historically assumed to have a more strongly laterally everted ilium than the Monachinae (Muizon, 1981). A preliminary quantitative study (Fig. S1; Table S12) based on a small sample of phocines and monachines supports this observation, but rather loosely. Overall, there appears to be a general trend, with the average lateral eversion of the phocine ilium exceeding that of the monachine by approximately nine percent (74.6° versus 65.3°, see Table S12). Despite the overlap between the measurement ranges of both subfamilies, statistical analysis (Fig. S2) indicates a significant difference in lateral eversion between both subfamilies. Although supported by statistical evidence, the differentiation between Phocinae and Monachinae on the basis of the lateral eversion of the ilium should be considered with care: due to the strong overlap in measurements, the applicability of this character is largest at the ranges of non-overlap but gradually decreasing within the range of overlap between both taxa. From that perspective, the innominate of *N. vitulinoides* is typically phocine, with an angle of 77.2°. The gluteal fossa is only weakly concave and is in some specimens hardly distinguishable from the general concavity of the lateral surface of the ilium. In extant phocids, monachines and *E. barbatus* completely lack a gluteal fossa, while the Baikal seal *Pusa sibirica* only has a faintly developed gluteal fossa (Bininda-Emonds & Russell, 1996). *Praepusa vindonbonensis* also has a poorly developed gluteal fossa, whereas in other extant and extinct phocine taxa, e.g., *B. neerlandica*, the gluteal fossa is more developed. Consequently, the area of origin of the glutei muscles is relatively weakly developed in *N. vitulinoides*, when compared to other phocines, but still better developed than in monachines and *E. barbatus*. The auricular surface for the articulation of the wing to the first sacral vertebra is deeply excavated, yielding a firm contact between the innominate and the sacrum. The iliac crest is slightly convex. Both anteroventral and anterodorsal processes are well developed, giving the iliac blade a distinctly triangular outline. The rounded posteroventral process is well developed, yielding a strongly concave ventral edge of the iliac blade between the anteroventral and posteroventral processes. Anterior and anteroventral to the acetabulum, two small fossae mark muscle attachment surfaces. The fossa anterior to the acetabulum is shallow and rounded; it most likely serves for the attachment of the *musculus rectus femoris*. The ventralmost fossa is much deeper and slightly more elongate anteroposteriorly; it is thought to serve for the attachment of *musculus sartorius* and *musculus iliacus*. The posterodorsal process reaches the same anterior level as the posteroventral process. The posterodorsal process is robust but only poorly raised dorsal to the level of the body of the ilium, as in other phocines. The acetabulum is deep and its edges are raised over the surrounding bone. The acetabular notch is deeper than it is wide, with a pronounced fossa at the bottom of the acetabulum. The iliopectineal eminence is small and blunt, and is located anterior to the anterior margin of the acetabulum, or level to it in some specimens. Except for *H. grypus*, the iliopectineal eminence is located much more posteriorly in all extant phocines. An important characteristic of the ischium is the strong development of an ischiatic spine on the anterodorsal margin of the iliac branch of the ischium (not to be confused with the ischiatic tuberosity, see below and Adam (2009)). Whereas this ischiatic spine is strongly reduced in all extant Pinnipedia Illiger, 1811, it makes a strongly posteriorly recurved eminence in *N. vitulinoides*. The anteriormost part of the base of the ischiatic spine is approximately at the same anteroposterior level as the anteriormost margin of the obturator foramen. Posteriorly, the ischium is strongly curving medially. The dorsal tip of the ischiatic tuberosity is well developed, similar to that of other phocines. The ischiatic spine and the ischiatic tuberosity are less developed in *Praepusa vindobonensis*. The ischiatic spine of *B. neerlandica* is well developed as well. However, the innominate of *B. neerlandica* is overall morphologically strongly similar to that of *N. vitulinoides*. Moreover, because the only known innominate of *B. neerlandica* has been found isolated, its assignment to the species remains questionable, and it may potentially represent an innominate of *N. vitulinoides*.

Contrasting with other phocines, the dorsolateral margin of the iliac branch of the pubis of *N. vitulinoides* is strongly transversely flattened. This strongly flattened surface is thought to correspond to the surface of origin for *musculus obturatorius externus*. The strong degree of flattening of this region in relation to the iliac branch of the pubis as a whole may be linked to a well-developed origin of the *musculus obturatorius externus*.

##### Femur

**Figure 20:**
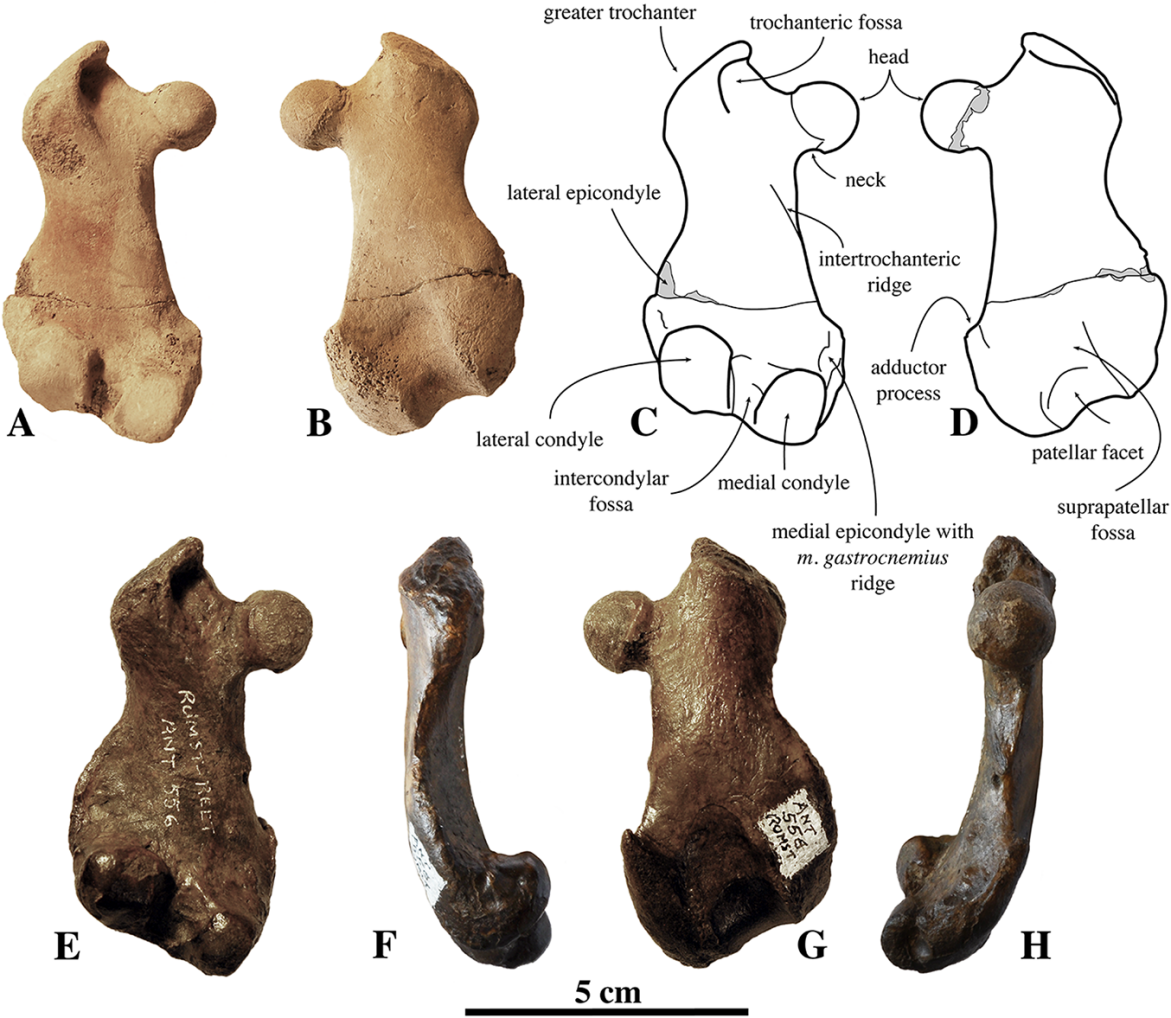
Femora of *Nanophoca vitulinoides*. IRSNB M2276e (neotype) left femur of *Nanophoca vitulinoides* (A, B) and corresponding drawings (C, D), in posterior (A, C) and anterior (B, D) view. IRSNB M2271 left femur of *Nanophoca vitulinoides* in posterior (E), lateral (F), anterior (G), and medial (H) view. Broken or obliterated areas are indicated in gray.

Apart from humeri, femora are the most commonly found bones assigned to *N. vitulinoides* (Fig. 20). Ten specimens in the IRSNB collection can be assigned unambiguously to *N. vitulinoides*, and more than 20 other femora are within the size range of the species and share several characters with the species, but are too poorly preserved to be identified unambiguously. In addition to the above specimens, Van Beneden (1877) illustrated IRSNB 1051-M251 as a left femur of *N. vitulinoides*. Although this specimen is badly preserved, a number of diagnostic characters of the femur are still visible. Similarly, specimen IRSNB 1049-M247 was also illustrated by Van Beneden (1877) as representing *N. vitulinoides*, and we concur on this identification. Finally, IRSNB 1102-M238 was identified as *Phocanella minor* by Van Beneden (1877), but in our opinion it clearly represents a specimen of *N. vitulinoides*. The head of the femur is subspherical in *N. vitulinoides*; the height/width ratio approximates 1. Multiple extinct phocines have a subspherical femoral head as well, e.g., *L. proxima*, *M. pontica*, and *Praepusa vindobonensis* (Koretsky, 2001). Most specimens of *N. vitulinoides* bear a noticeable pit for the *teres femoris* ligament on the femoral head. Such a pit is generally not developed in most Pinnipediformes and is considered to be a primitive trait. The greater trochanter reaches a much more proximal level than the head, a condition similar to the extant *P. groenlandicus*, and *Pusa*, as well as many extinct phocines such as *Leptophoca*, and *Praepusa vindobonensis* (Koretsky, 2001). The greater trochanter is roughly triangular in cross-section. In posterior view, the outline of the greater trochanter is subrectangular and the trochanteric fossa opens medially and is proximally capped by a thin lip. The area of the lesser trochanter is represented by a low, but well-defined elevation located slightly distal to the head, on the posteromedial edge of the femur. However, this feature is not present in all specimens of *N. vitulinoides* (present in, e.g., IRSNB M2276d,e; neotype). Nevertheless, no extant phocines and few extinct phocines (*C. maeotica*, *L. proxima*, *Praepusa vindobonensis*, and *S. sintsovi*) bear a lesser trochanter (Koretsky, 2001). A lowly raised but transversely broad intertrochanteric ridge connects the lesser and greater trochanters. The general shape of the diaphysis is typically pinniped, i.e., with a transverse section mediolaterally broad and anteroposteriorly flattened. The mediolateral width of the femur of *N. vitulinoides* is greater in relation to the femoral epiphyses than in *Praepusa vindobonensis*. The minimum mediolateral width is in the proximal portion of the diaphysis. The deep patellar facet is wider than it is high. While the width/height ratio of the patellar facet corresponds to that of other phocines, its deep concavity separates *N. vitulinoides* from extant phocines. The suprapatellar fossa is as large as the patellar facet; it forms also a prominent depression within the bone. This condition strongly differs from that in extant phocids, where such a suprapatellar fossa is either much reduced or absent. The patellar facet of monachines is anteriorly raised over the distal epiphysis and wider than it is high (Muizon, 1981), whereas the patellar facet of phocines is at the level of the distal epiphysis, but it has slightly elevated lateral margins. Moreover, in the latter the patellar facet is higher than it is wide. This difference between extant monachines and phocines implies a larger mobility of the patella, i.e., knee joint, in phocines as compared to monachines (Muizon, 1981). The depth of the suprapatellar fossa may have further increased the extension capability of the knee joint. As is typical for pinnipedimorphs (Berta & Ray, 1990; Berta & Wyss, 1994), the distal epiphysis of the femur of *N. vitulinoides* is strongly asymmetrical, with the medial condyle located much more distally than the lateral condyle. The lateral condyle is only slightly larger than the medial condyle. The intercondylar fossa is deep and narrow, gradually becoming narrower with increasing depth of the fossa. The medial epicondyle forms a distinct ridge and bears a prominent proximally directed adductor tubercle. A similar adductor tubercle is not uncommon among phocines, such as *Phoca vitulina*, but it is rarely as pronounced. At the base of the medial epicondyle, just proximal to the medial condyle, there is a small and low proximodistally elongated ridge in *N. vitulinoides*, approximately 5 mm in length, 1 mm in width, and 1 mm in height. This ridge serves as an attachment site for the medial head of *musculus gastrocnemius*. We observed the same feature in several specimens of *H. grypus* and *H. fasciata*. However, given the fact that this feature is not present in all studied specimens of the latter species, and given the limited number of specimens of extant seals investigated, this character may also be present in other phocine species. The lateral epicondyle does not expand far laterally in *N. vitulinoides*, but does so proximally; it has a smoothly rounded shape. The medial and lateral epicondyles are relatively thick and robust, compared to other phocines. Although both known femora attributed to *B. neerlandica*, n°10373 and MAB 4342, do not differ noticeably from those of *N. vitulinoides*, it should be noted that the state of preservation of the former is poor to moderate.

##### Tibia–Fibula

**Figure 21:**
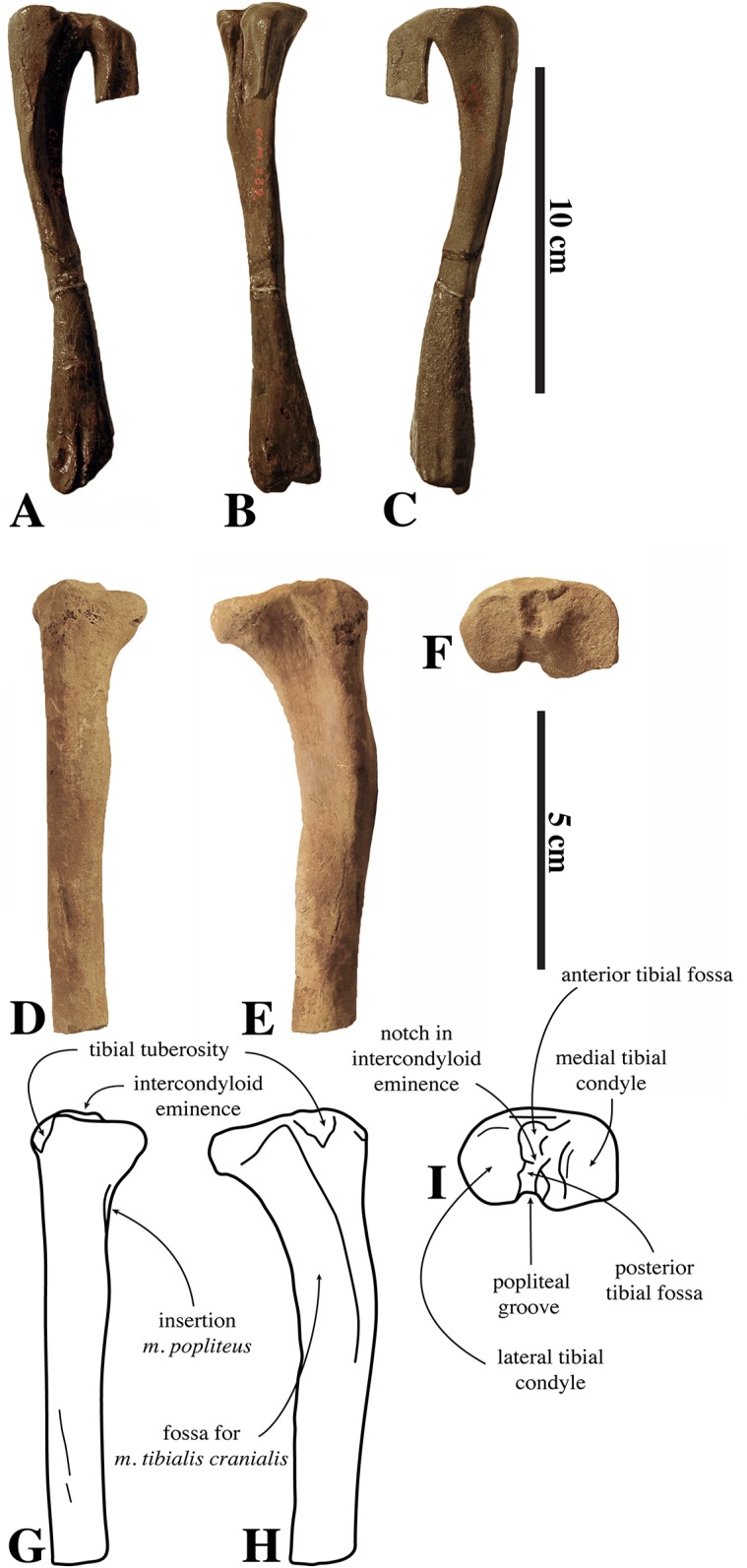
Tibiae of *Nanophoca vitulinoides*. IRSNB 1105-M239 right tibia (A–C) in posterior (A), medial (B), and anterior (C) view. IRSNB M2276g (neotype) left tibia of *Nanophoca vitulinoides* (D–F) and corresponding drawings (G-I) in lateral (D, G), anterior (E, H), and proximal (F, I) view.

Currently, there are 21 tibiae in the collection of the IRSNB that can be assigned to *N. vitulinoides* with a high degree of certainty (Fig. 21). Tibiae are assigned to *N. vitulinoides* on the combined basis of their small size, the strong concavity of the insertion for the *musculus popliteal*, and the overall morphology of the tibial plateau. Because the tibia and fibula of phocids are fused (except for the Hawaiian monk seal *Monachus schauinslandi*), some are preserved together with the fibula. Two tibiae are almost completely preserved (IRSNB 1069-M248 and IRSNB 1105-M239). Although Van Beneden (1877) identified specimens IRSNB 1090-M239 and IRSNB 1105-M239 as *Phocanella pumila* and *Phocanella minor*, respectively, we reassign both specimens to *N. vitulinoides* on the basis of their overall same size and shape (including diagnostic features) to *N. vitulinoides*. Both femoral articular facets are slightly concave and the medial condyle is noticeably smaller than the lateral condyle (see Table S6). The intercondyloid eminence is highly raised over the two adjacent tibial condyles and is split by a notch. The shape of the tibial plateau of *N. vitulinoides* conforms with that of other phocines. Anterior to this intercondyloid eminence, the anterior tibial fossa is oval, shallow compared to other Phocinae, and opens anterolaterally. On the cranial margin of the tibial plateau, the tibial tuberosity for the insertion of the quadriceps femoris muscle is a well-outlined equilateral triangle. Posterior to the intercondyloid eminence, the posterior tibial fossa is deep and sloping posteriorly into the popliteal notch. The posterior tibial fossa is predominantly located adjacent to the medial tibial condyle and mostly separated from the lateral facet by the popliteal notch. The latter is deep, relatively narrow, and semicircular in section. The diaphysis of the tibia is slender and strongly curves sigmoidally in anterior view. In the closely related *Praepusa vindobonensis*, the tibia shows a lesser curvature. Compared to other Phocinae, the diaphysis of the tibia of *N. vitulinoides* is less flattened anteroposteriorly, but rather is subcircular in cross-section. On the proximal part of the cranial surface, the fossa for *musculus tibialis cranialis* is moderately well developed but well outlined, as well as the facet for *musculus biceps femoris* on the medial surface. Posteriorly, the attachment surfaces for *musculus tibialis caudalis* and *musculus popliteus* are well developed. The latter is very well developed, yielding a relatively strong concave incursion in the bone, not observed in other phocines. The deep, short attachment surface for *musculus flexor digitorum longus* is located very proximal. At mid-length, the diaphysis is subcircular and much narrower than at its extremities. The distal fibular facet has the shape of an isosceles triangle, strongly elongated along the axis of the bone. The distal epiphysis is relatively thick and sub rectangular in cross-section. The astragalar facet is sub-triangular to sub-rounded, in distal view, and moderately sloping mediodistally. On its posterior surface, the epiphysis and the distal part of the diaphysis bear two deep grooves defined by three sharp ridges. These grooves are for the tendons of *musculus tibialis cranialis* (medial) and *musculus flexor digitorum longus* (lateral). On the anterior surface of the distal epiphysis, one deep groove most likely accommodated the tendons of *musculus extensor hallucis longus* and *musculus tibialis cranialis*. The small proximal facet of the fibula is triangular in cross-section and strongly laterally sloping.

The proximal extremity of the diaphysis of the fibula is triangular, marked by three well-outlined surfaces for muscle attachment: *musculus extensor hallucis longus* (medial), *musculus peroneus brevis* (anterolateral), and *musculus flexor hallucis longus* (posterolateral). The area to accommodate the latter is the least excavated. Posteriorly on the proximal epiphysis, a narrow but deep fossa serves to accommodate the tendon of the *musculus flexor hallucis longus*.

**Figure 22:**
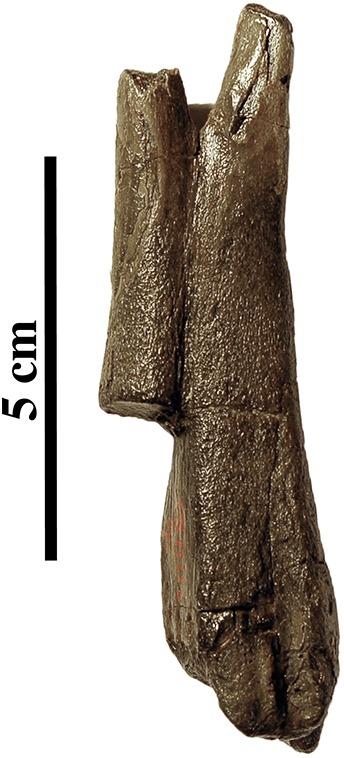
Pathologic tibia and fibula. IRSNB 1300-M250 right fibula and tibia of *Nanophoca vitulinoides* fused. Note that the bone cortex covers both bones and fusion is pathological, not diagenetic.

One specimen illustrated by Van Beneden (1877), IRSNB 1300-M250, should be noted, because the diaphyses of the tibia and the fibula are fused for the major part of the preserved portion (Fig. 22). Fusion of the distal epiphysis of the tibia points toward sexual maturity, based on comparison with skeletal growth in extant phocines (Storå, 2000). Hence, if this fusion would have happened during growth, it does not appear to have provided the individual any biological disadvantage that would have prevented it from reaching adulthood. Otherwise, fusion may also have happened pathologically in a skeletally mature individual.

##### Calcaneum

**Figure 23:**
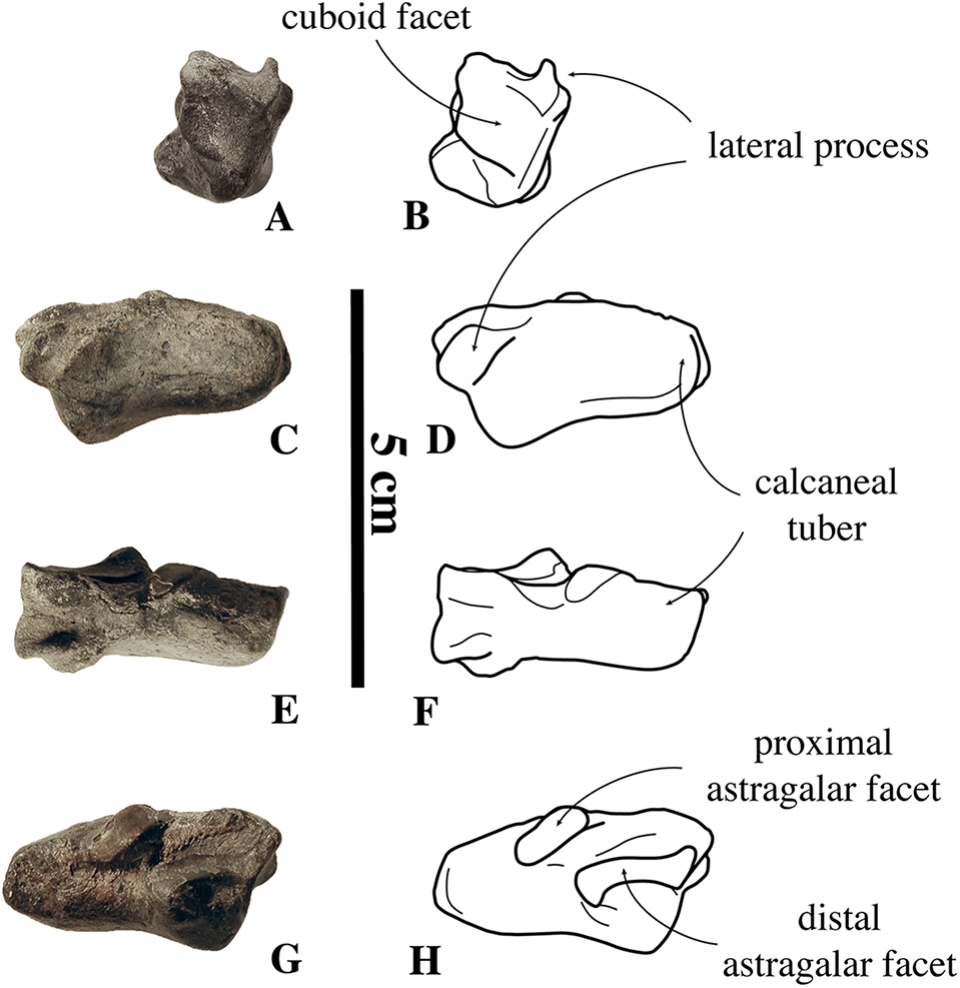
Calcaneum of *Nanophoca vitulinoides*. IRSNB M2275 right calcaneum of *Nanophoca vitulinoides* (A, C, E, G) and corresponding drawings (B, D, F, H) in distal (A, B), medial (C, D), dorsal (E, F), and lateral (G, H) view.

Currently, one right (IRSNB M2275) calcaneum has been assigned to *N. vitulinoides* (Fig. 23). Overall, the calcaneum is typically phocine: it is not particularly elongated and slightly curved in lateral view, with the calcaneal tuber slightly projecting plantarly. The proximal astragalar facet (=ectal facet) is strongly convex and short, while the distal astragalar facet (=sustentacular facet) is slender. The lateral process for the tendon of *musculus peroneus longus* is located rather dorsally, reaching only slightly plantar to the dorsal margin of the cuboid facet. The cuboid facet is sub rounded, contrasting to other phocines, which have a slightly more elongate cuboid facet and a lateral process more centrally located on the lateral margin of the calcaneum. In the absence of the astragalus, it is impossible to make any inferences on the length and shape of the calcaneum in relation to the astragalus and, hence, on the mobility of the foot.

##### Astragalus

Two or three potential astragali are known for *N. vitulinoides*. Two have been mentioned by Van Beneden (1871); although mentioned and described as calcanea, he clearly illustrates an astragalus (Van Beneden, 1871, pl. 1, Fig. 3). However, the specimens have been lost and will not be considered for the current study; not in the least because their identification as belonging to *N. vitulinoides* [*Phoca vitulinoides*] is very tentative. Recently, an astragalus has been found in the collection at the KUL. It is unclear whether it represents a previously undescribed astragalus or the astragalus that Van Beneden (1871) mentioned but did not illustrate. It is large, when compared to other bones assigned to *N. vitulinoides*. Hence, we question whether this astragalus represents *N. vitulinoides* or not.

##### Metatarsals

Currently, only one right fourth metatarsal from the partial skeleton IRSNB M2276h has been assigned to *N. vitulinoides*. However, assignment of this metatarsal to the species is not without doubt. Compared to other bones of this specimen, color differences and the relatively large size cause us to question its assignment. Yet, its association with the most complete known fossil seal specimen from the Antwerp area favors the tentative assignment of this metatarsal to *N. vitulinoides*. Nevertheless, this metatarsal does not bear any remarkable visible traits to distinguish it from other phocines.

##### Phalanx

Among the original collection described by Van Beneden (1871), there was one phalanx assigned to *Phoca vitulinoides*. Unfortunately, the original specimen is lost and one illustration of the palmar/plantar side of the phalanx cannot be redescribed and reassessed critically. Moreover, in the absence of phalanges associated to other specimens of *N. vitulinoides* the designation of this isolated phalanx as belonging to *N. vitulinoides* [*Phoca vitulinoides*] remains doubtful.

### Body length estimates

The body length estimates for *N. vitulinoides* yield an average extrapolated body length of 55.4 ± 3.06 cm for the estimate based on *Pusa hispida* (Howell, 1929), 92 ± 3.21 cm for the estimate based on *Pusa sibirica* (this study), 104.5 ± 5.33 cm for the estimate based on *Phoca vitulina* (this study), 122.4 ± 11.36 cm for the estimate based on *L. weddelli* (Piérard, 1971), and 134.4 ± 12.40 cm for the estimate based on *O. rossi* (Piérard & Bisaillon, 1975) (Table 1). Given the large discrepancy between the body length extrapolated from *P. hispida* versus *L. weddelli* and *O. rossi*, based on data from previous studies it is inappropriate to calculate a gross average before the discussion of the results (see Discussion).

**Table 1:** Body size estimations for *Nanophoca vitulinoides*. Body size estimations for *Nanophoca vitulinoides* based on comparison with *Pusa hispida* (Howell, 1929), *Leptonychotes weddelli* (Piérard, 1971), and *Ommatophoca rossi* (Piérard & Bisaillon, 1975).

Specimen Number	Bone	Bone length (mm)	Percentage (Howell, 1929)	Estimated total length (mm) (Howell, 1929)	Percentage (Piérard, 1971)	Estimated total length (mm) (Piérard, 1971)	Percentage (Piérard & Bisaillon, 1975)	Estimated total length (mm) (Piérard & Bisaillon, 1975)	Percentage (*Phoca vitulina*) (this study)	Estimated total length (mm) (*Phoca vitulina*) (this study)	Percentage (*Pusa sibirica*) (this study)	Estimated total length (mm) (*Pusa sibirica*) (this study)
IRSNB M2276c	Humerus	72.4	14	51.7	6.50	111.4	5.95	121.7	7.76	93.3	8.12	89.2
IRSNB 1063-M242	Humerus	78.2	14	55.9	6.50	120.3	5.95	131.4	7.76	100.8	8.12	96.3
IRSNB M2278	Radius	77.1	14	55.1	7.10	108.6	6.00	128.5	N/A	N/A	N/A	N/A
IRSNB M2271	Femur	71.5	12	59.6	5.22	137.0	4.71	151.8	6.47	110.5	7.73	92.5
IRSNB M2276d	Femur	69.5	12	57.9	5.22	133.1	4.71	147.6	6.47	107.4	7.73	89.9
IRSNB 1105-M239	Tibia	151.9	29	52.4	12.28	123.7	12.14	125.1	N/A	N/A	N/A	N/A

**Note:**

Measurements expressed in mm.

### Phylogenetic analysis

No phylogenetic analyses including *N. vitulinoides* have been published before. The aim of the phylogenetic study presented here is to resolve the affinities of *N. vitulinoides* and to reassess the phylogenetic position of the genus *Praepusa* among Phocidae. The character matrix is presented in Data S1.

**Figure 24:**
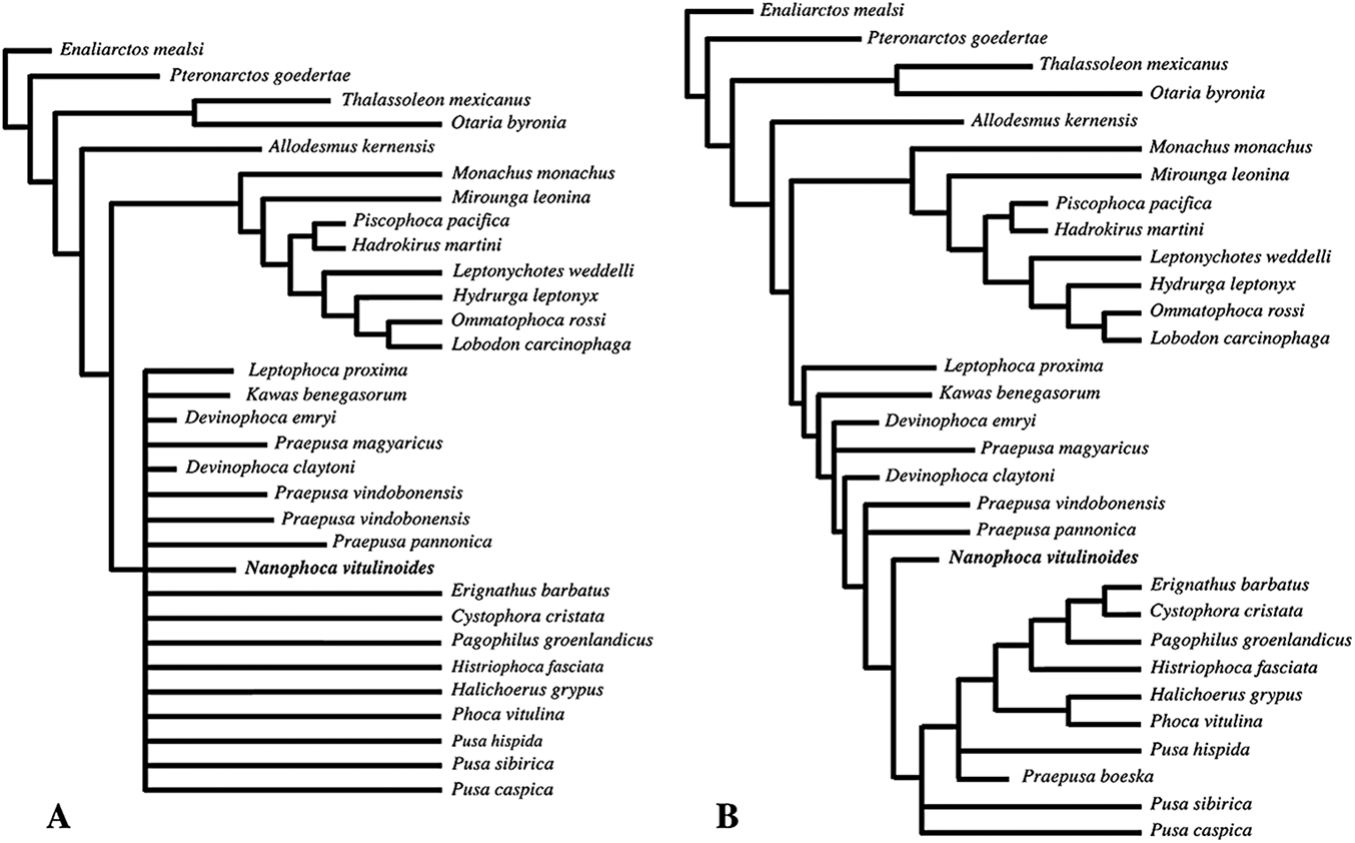
Primary phylogenetic trees. Strict (A) and 50% majority (B) consensus phylogenetic trees including *Praepusa magyaricus* and *Praepusa panonnica* based on 736 most parsimonious trees with score 254. In the strict (A) consensus tree, Phocinae are poorly resolved. In the 50% majority (B) consensus tree, all extinct Phocinae return as stem phocines, but the phylogenetic relationships of extant Phocinae differ from molecular and other morphological phylogenetic analyses (see text).

**Figure 25:**
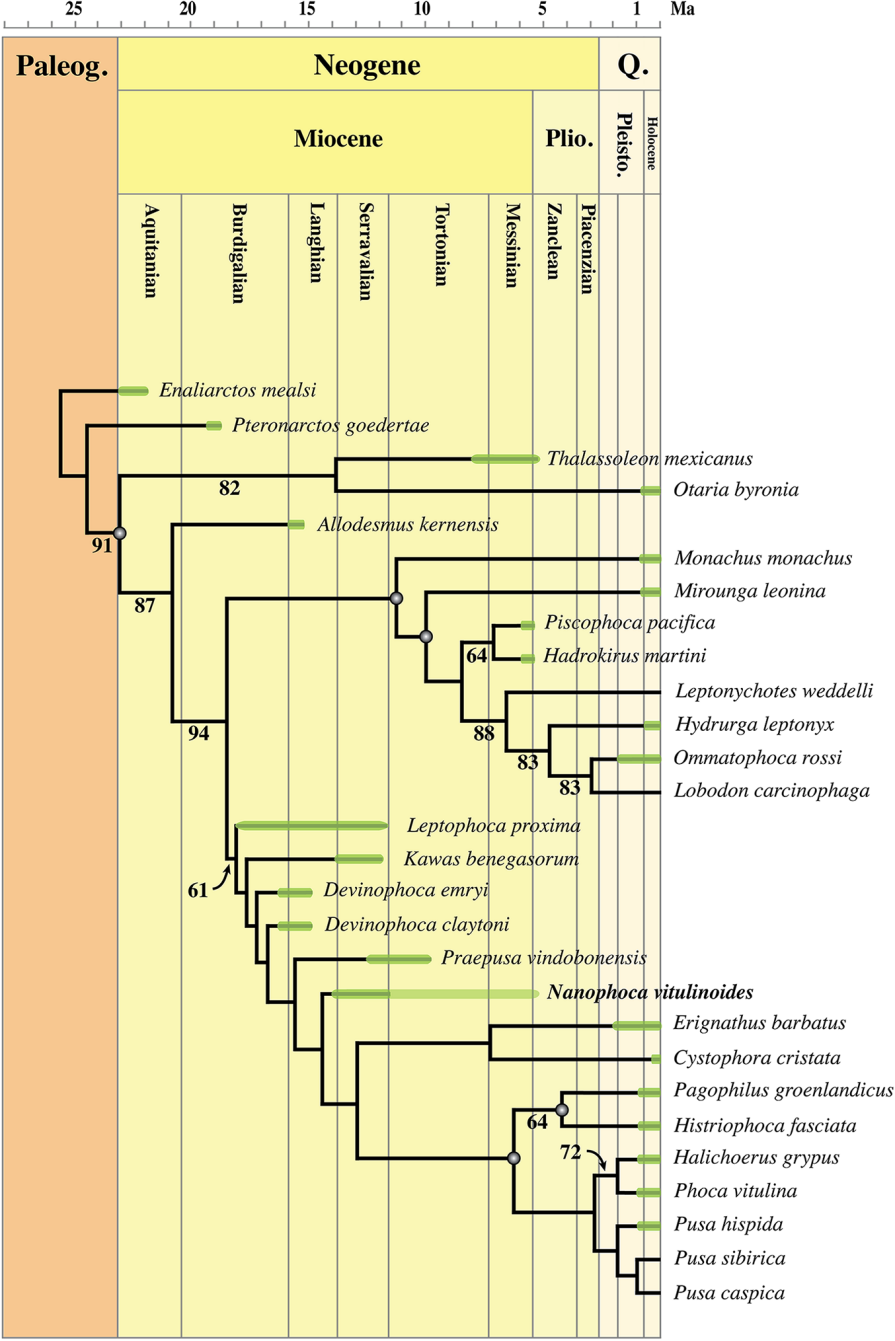
Phylogenetic tree of Phocidae. Most parsimonious phylogenetic tree used to elucidate the phylogenetic relationships of *Nanophoca vitulinoides* among other genera of Phocinae. Time-calibration of nodes is performed using Higdon et al. (2007) and all corresponding time-calibrated nodes are indicated by a gray dot. The age ranges for extinct OTUs are expressed as a green bar over each relevant terminal branch; uncertainty regarding the upper age of *Nanophoca vitulinoides* is expressed as a light green bar. Bootstrap values exceeding 50% are indicated on the relevant branches. Geochronologic ages for the included specimens, whenever fossil or subfossil specimens have been documented: *Allodesmus kernensis* (Barnes, 1988), *Cystophora cristata* (Andreasen, 1997), *Devinophoca claytoni* (Koretsky & Holec, 2002), *Devinophoca emryi* (Koretsky & Rahmat, 2015), *Enaliarctos mealsi* (Mitchell & Tedford, 1973), *Erignathus barbatus* (West, 1980; Harington, 2008), *Hadrokirus martini* (Amson & Muizon, 2014), *Halichoerus grypus* (Repenning, 1983), *Histriophoca fasciata* (Repenning, 1983), *Hydrurga leptonyx* (Kellogg, 1922; Fleming, 1968), *Kawas benegasorum* (Cozzuol, 2001), *Leptophoca proxima* (Dewaele, Lambert & Louwye, 2017), *Mirounga leonina* (Avery & Klein, 2011), *Monachus monachus* (Stringer et al., 2008), *Nanophoca vitulinoides* (this study), *Ommatophoca rossi* (Fleming, 1968), *Otaria byronia* (Drehmer & Ribeiro, 1998), *Pagophilus groenlandicus* (Repenning, 1983), *Phoca vitulina* (Repenning, 1983), *Piscophoca pacifica* (Amson & Muizon, 2014), *Praepusa vindobonensis* (Koretsky, 2001), *Pteronarctos goedertae* (Berta, 1994), *Pusa hispida* (Repenning, 1983; Harington, 2008), and *Thalassoleon mexicanus* (Deméré & Berta, 2005).

The phylogenetic analysis including all 31 OTUs, without Goloboff criterion, resulted in 736 most parsimonious trees, with a best score of 254 steps after 3,909,435 tried rearrangements. The strict consensus and 50% majority consensus trees are shown below (Fig. 24). In the strict consensus tree (Fig. 24A), the Phocinae remain unresolved, while in the 50% majority consensus tree (Fig. 24B), most extinct Phocinae appear as successive branches of stem Phocinae. *Praepusa boeska* is nested among the extant Phocinae, but the tree of extant Phocinae contrasts markedly with previously published morphological and molecular analyses (Muizon, 1981; Bininda-Emonds & Russell, 1996; Higdon et al., 2007; Fulton & Strobeck, 2010). Therefore, a second analysis has been performed with the exclusion of *Praepusa boeska*, *Praepusa magyaricus*, and *Praepusa pannonica*. These three OTUs have been scored for relatively few characters. Additionally, the *k*-value of the Goloboff criterion has been set at three, for down-weighting homoplastic characters. The analysis resulted in one most parsimonious phylogenetic tree (Fig. 25) with score −63.07 after 9,409 tried rearrangements. Consistency index (CI) is 0.42 (0.415 excluding parsimony-uninformative characters), homoplasy index (HI) is 0.58 (0.585 excluding parsimony-uninformative characters), retention index (RI) is 0.74, and rescaled consistency (RC) index is 0.31.

All extinct phocine taxa included in this analysis (*N. vitulinoides*, *K. benegasorum*, *L. proxima*, and *Praepusa vindobonensis*) as well as the Devinophocinae (*Devinophoca claytoni* and *Devinophoca emryi*) return as stem phocines in our analysis. *L. proxima* and *K. benegasorum* are the first and second stem phocines to branch off, followed by *Devinophoca emryi* and *Devinophoca claytoni*, and finally *Praepusa vindobonensis* and *N. vitulinoides* before crown Phocinae.

A complete list of the apomorphies that resulted from the phylogenetic analysis is provided as Fig. S3 and Table S13. In the phylogenetic analysis, the identification of *N. vitulinoides* as a separate taxon is supported by one equivocal and two unequivocal autapomorphies: two ridges on lateral side of scapula join near glenoid (character 44, state “0” to “1;” unequivocal); distal epiphysis wider than proximal epiphysis (character 72, state “1” to “0;” equivocal); and greater trochanter of femur reaches more proximal than head (character 74, state “1” to “2;” unequivocal).

## Discussion

### Body length

**Figure 26:**
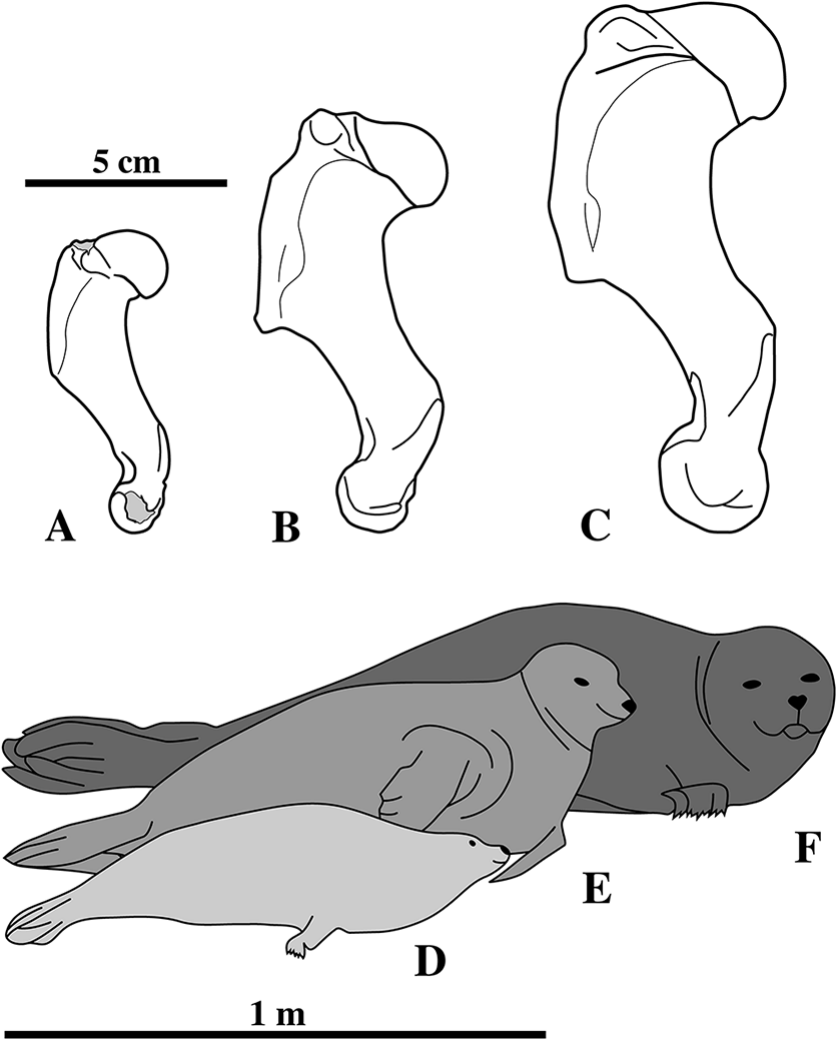
Size variations in Phocinae. Size comparison of the humerus of *Nanophoca vitulinoides* (A) with *Pusa sibirica* (B) and *Phoca vitulina* (C) and corresponding total body size reconstructions (D, E, and F, respectively).

Bones of *N. vitulinoides* are among the smallest among pinnipeds (Table 1; Fig. 26; Tables S1–S8). Only the extinct *B. neerlandica* and *M. pontica* have smaller limb bones (Koretsky, 2001; Koretsky & Peters, 2008).

Extrapolation from the phocine *Pusa hispida* (presented as *Phoca hispida*; Howell, 1929) points toward an estimated body length (snout to tail length) between 50 and 60 cm. In contrast, extrapolations from the monachines *L. weddelli* (Piérard, 1971) and *O. rossi* (Piérard & Bisaillon, 1975) yield a range between 110 and 150 cm. It can be argued that the large discrepancy between the extrapolated ranges based on *P. hispida* versus *L. weddelli* and *O. rossi* might be rooted in the ambiguity of the measurements for *Phoca hispida* by Howell (1929). Other than publishing direct length measurements, measurements are published by the latter as ratios to animal length. However, it is unclear whether Howell considered the thoracolumbar column length or the snout-to-tail length for total length. In order to test the validity of these extrapolations, the absolute length of the humeri and femora of *Phoca vitulina* and *Phoca sibirica* specimens from the IRSNB and MSC was measured. Because direct total length measurements were impossible due to the incompleteness of the skeletons, ratios have been calculated in relation to the average adult body size reported for each species: 150 cm for *Phoca vitulina* (see Storå, 2000) and 127 cm for *Phoca sibirica* (Ciesielski et al., 2006). The calculated ratios (Table 1) are higher than the values retrieved from Piérard (1971) and Piérard & Bisaillon (1975), but still much closer to those than to the values retrieved from Howell (1929). The extrapolated body length of *N. vitulinoides* is 92 cm based on the data from *Phoca sibirica* and 104.5 cm based on the data from *Phoca vitulina* (see also Table S14).

Given the absence of a more complete skeleton including cranial material, body length estimates for *N. vitulinoides* are based on extrapolations from single postcranial bones. Although cranial measurements prove to be valuable for body size estimation (Churchill, Clementz & Kohno, 2014), postcranial measurements appear to be less valuable and consistent (compare Howell, 1929; Piérard, 1971; Piérard & Bisaillon, 1975; this study). However, a total adult body length of approximately 100 cm (1 m) can be safely considered for *N. vitulinoides*.

Because only the extinct *B. neerlandica* and *M. pontica*, also from the late Miocene of the Netherlands and the Paratethys, respectively, have slightly smaller limb bones than *N. vitulinoides*, it can be noted that a number of middle and late Miocene phocids from Europe are considerably smaller than extant phocids. One hypothesis that may explain the small size of middle and late Miocene European phocids is the influence of unusually warm sea surface temperatures (SSTs) during the middle Miocene climatic optimum (MMCO). Because heat loss is higher in smaller organisms, it puts a lower limit on the possible body size range in marine endotherms (Whittow, 1987; Downhower & Blumer, 1988). However, different simulations for SST duing the MMCO show contrasted results, ranging from lower to similar to higher equatorial SSTs during the MMCO than today SSTs (You et al., 2009). In these simulations, maximum equatorial SST discrepancies with today’s SSTs are less than 5 °C. Also, simulated Arctic SSTs are consistently in the order of 5 °C higher than today’s Arctic SSTs. Hence, SSTs in temperate zones are difficult to infer and it can be argued that the impact of MMCO on the size of middle Miocene phocids was relatively limited. A correlation between body size and temperature variation during the middle Miocene is further mitigated by the presence of relatively large seals as well, such as *L. proxima* and *P. rousseaui* (Dewaele, Lambert & Louwye, 2017). Moreover, for pinnipedimorphs in general, Churchill, Clementz & Kohno (2015) did not observe any variation in minimum body size during their evolutionary history. In addition, there is no present-day analog for this hypothesis, because the smallest extant Phocidae include *Pusa caspica* and *Phoca sibirica*, which both live in temperate zones, while the genus *Monachus*, the only subtropical to tropical phocid, is relatively large.

Home range, and by consequence bioenergetics and nutrient availability, is a more plausible characteristic controlling mammal body size (McNab, 1963; Lindstedt, Miller & Buskirk, 1986). While most Pliocene Phocidae from Europe have also been found in North America, only the larger Miocene *L. proxima* has a trans-Atlantic range (Koretsky & Ray, 2008; Dewaele, Lambert & Louwye, 2017). All other European and Paratethyan phocids are strongly endemic and have so far not been found in North American deposits or elsewhere (Koretsky, 2001; Koretsky & Rahmat, 2013). Of these presumably endemic species, all but few species, e.g., *C. maeotica*, *Devinophoca* spp., and *P. rousseaui*, appear to be smaller than the smallest extant phocines (Koretsky, 2001; Koretsky & Holec, 2002; Koretsky & Rahmat, 2013; Rahmat & Koretsky, 2016). In support, extant marine mammals also show a correlation between body size and home range size: the phocid *Pusa sibirica*, the Galápagos fur seal *Arctocephalus galapagoensis*, and the vaquita *Phocoena sinus*, are the smallest phocid, otariid and cetacean, respectively, and they occupy the smallest ranges within their respective clades.

Nutrient availability and bioenergetics are related to the range size (McNab, 1963; Lindstedt, Miller & Buskirk, 1986). Although it is difficult to induce lifestyles and feeding behavior for fragmentary and incompletely known extinct taxa, the anatomy of *N. vitulinoides* and other extinct phocids, e.g., *B. neerlandica* and *M. pontica*, indicate different modes of locomotion (see below) and, hence, most likely, different modes of prey capture and feeding behavior compared to extant many Phocidae.

### Functional anatomy of *N. vitulinoides* with notes on lever arms in anatomy

#### Lever arms in anatomy

**Figure 27:**
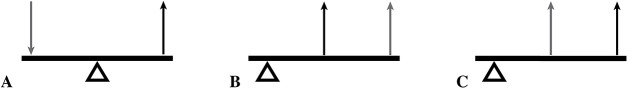
Lever classes. (A) Class one lever with fulcrum located between load and effort arm. (B) Class two lever with load located between fulcrum and effort arm. (C) Class three lever with effort arm located between fulcrum and load. The third class lever is the most commonly encountered lever class in a vertebrate animal’s body. Fulcrum = triangle, load = black arrow, effort = gray arrow. Arrows indicate direction of movement during action.

Levers are either used to obtain a mechanical advantage or to increase speed. A mechanical advantage is achieved when the distance of the effort arm to the fulcrum is large compared to the distance between the fulcrum and the load arm, enabling to translocate a larger load with less effort. An example is lifting a car by using a jack: a heavy object is lifted with relatively little effort (Fig. 27). On the other hand, speed can be increased when the effort arm is shorter than the load arm, e.g., lifting a shovel (Fig. 27). Hence, power and energy can be increased by increasing the length of the effort arm or by decreasing the load arm. The opposite is true to increase speed over mechanical advantage (see Davidovits, 2012; Fig. 27). There are three classes of lever within the vertebrate body (see Davidovits, 2012). In first class levers (Fig. 27A), the fulcrum is located between the effort and the load. First class levers are generally rare in vertebrate organisms and may either operate at a mechanical advantage or at speed. One example of a first class lever within the vertebrate body is the head-atlas joint. Acting as a fulcrum, contraction of muscles in the neck lift up the face. In second class levers (Fig. 27B), the load is applied between the effort and the fulcrum. Second class levers always act at a mechanical advantage, reducing speed and range of motion. An example of a second class lever in the body is the foot. With the toes acting as a fulcrum, force is applied to the metatarsals and phalanges, lifting the heel during running. Third class levers (Fig. 27C) are the most common levers within the vertebrate body, with the effort applied between the load and the fulcrum. Third class levers increase speed of the action. One example of a third class lever in the body is the elbow joint. When contracting the biceps, the forearm lifts up.

##### Cervical versus lumbar vertebrae

For the axial skeleton, the main anatomical differences between Phocidae and Otariidae are situated at the level of the cervical and lumbar vertebrae. The spinous processes and transverse processes of the lumbar vertebrae are better developed in Phocidae than in Otariidae, while in the latter the spinous processes and transverse processes of the cervical vertebrae are better developed than in Phocidae (Berta, Sumich & Kovacs, 2006; Pierce, Clack & Hutchinson, 2011; Kuhn & Frey, 2012). Moreover, in Phocidae, the cervical vertebrae are considerably shorter than the lumbar vertebrae, while in Otariidae, the bodies of cervical and lumbar vertebrae are of roughly equal dimensions. The better development of the processes of the lumbar vertebrae in Phocidae provides larger attachment surfaces for the hypaxial musculature (*musculus quadratus lumborum*, *musculus longissimus thoracis*, and *musculus iliocaudalis*; Berta, Sumich & Kovacs, 2006), correlating with horizontal movements in the posterior end of the body. Hence, this characteristic is an adaptation to the prominent use of hind flippers and pelvic oscillations for aquatic locomotion in Phocidae, contrasting with the Otariidae mode of swimming (Berta, Sumich & Kovacs, 2006; Pierce, Clack & Hutchinson, 2011; Kuhn & Frey, 2012). The enlarged processes of the relatively large cervical vertebrae in Otariidae provide enlarged attachment surfaces for the epaxial musculature (*musculus multifidus lumborum*, and *musculus longissimus thoracis*) (Berta, Sumich & Kovacs, 2006). Otariidae require a reinforced neck musculature, compared to Phocidae, not only because they heavily rely on pectoral oscillations for aquatic locomotion, but also for terrestrial locomotion. During terrestrial locomotion, Otariidae actively use their fore flippers to locomote on land, while Phocidae rarely do so (O’Gorman, 1963; English, 1977; Muizon, 1981; Berta & Ray, 1990; Berta, Sumich & Kovacs, 2006; Pierce, Clack & Hutchinson, 2011; Kuhn & Frey, 2012). The axial skeleton of *N. vitulinoides* is very similar to that of other Phocidae; *N. vitulinoides* has small cervical vertebral bodies in comparison to the lumbar vertebral bodies and it has small processes on the cervical vertebrae and large processes on the lumbar vertebrae (Table S1). Hence, regarding the axial skeleton, terrestrial and aquatic locomotion of *N. vitulinoides* is concordant with other Phocidae: prevailing use of the pelvis for aquatic locomotion and reduced use for terrestrial locomotion as well as globally reduced use of the pectoral girdle (i.e., fore flippers) for both aquatic and terrestrial locomotion.

#### Pectoral girdle

##### Humerus

**Figure 28:**
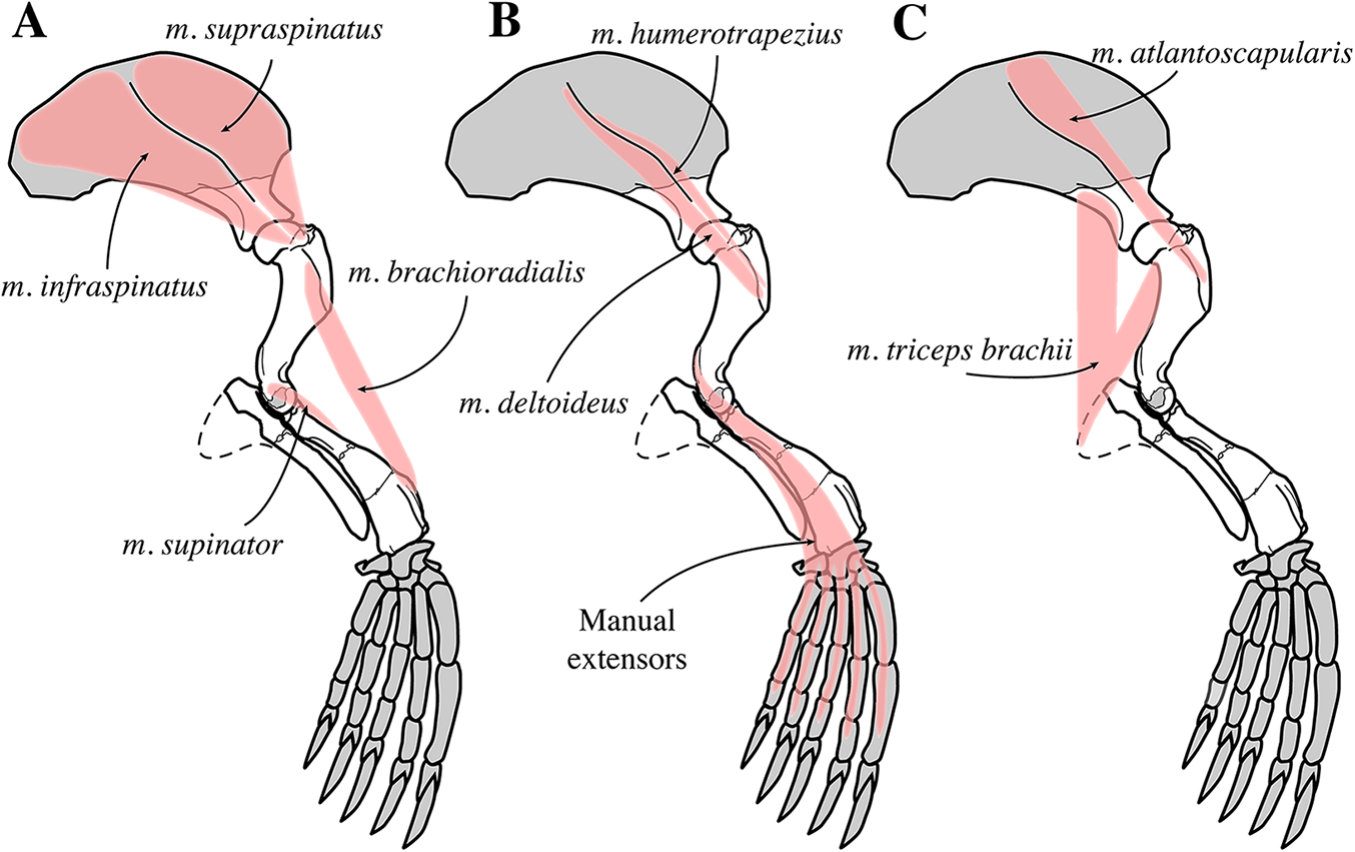
Fore limb musculature of *Nanophoca vitulinoides* in lateral view. The origin and insertion of selected muscles of the fore limb that are visible in lateral view. Muscles indicated in pink. Missing bones or bone parts of *Nanophoca vitulinoides* indicated in gray. Dashed line visually completes the ulna. This illustration focuses on the visualization of the origin and insertion of different muscles. Hence, the actual shape of the muscles may differ from this illustration.

**Figure 29:**
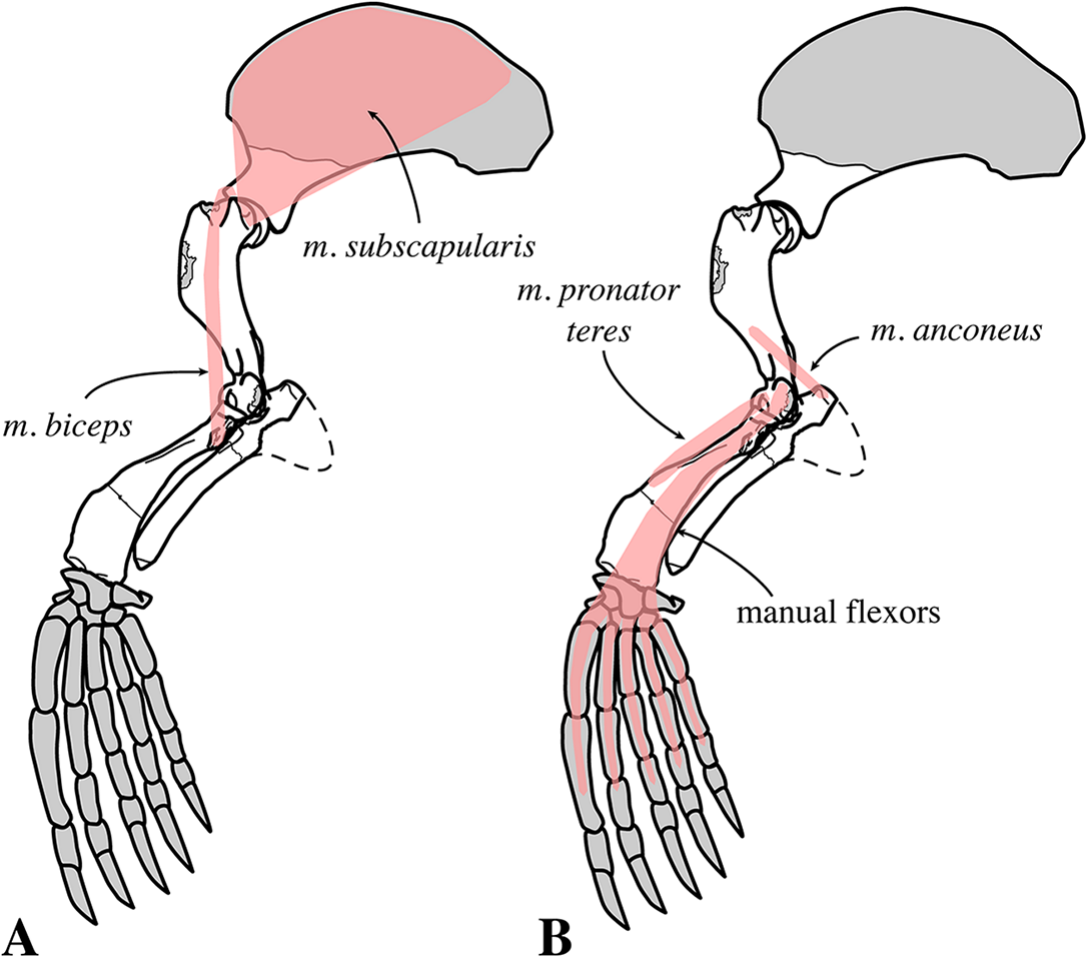
Fore limb musculature of *Nanophoca vitulinoides* in medial view. The origin and insertion of selected muscles of the fore limb that are visible in medial view. Muscles indicated in pink. Missing bones or bone parts of *Nanophoca vitulinoides* indicated in gray. Dashed line visually completes the ulna. This illustration focuses on the visualization of the origin and insertion of different muscles. Hence, the actual shape of the muscles may differ from this illustration.

**Figure 30:**
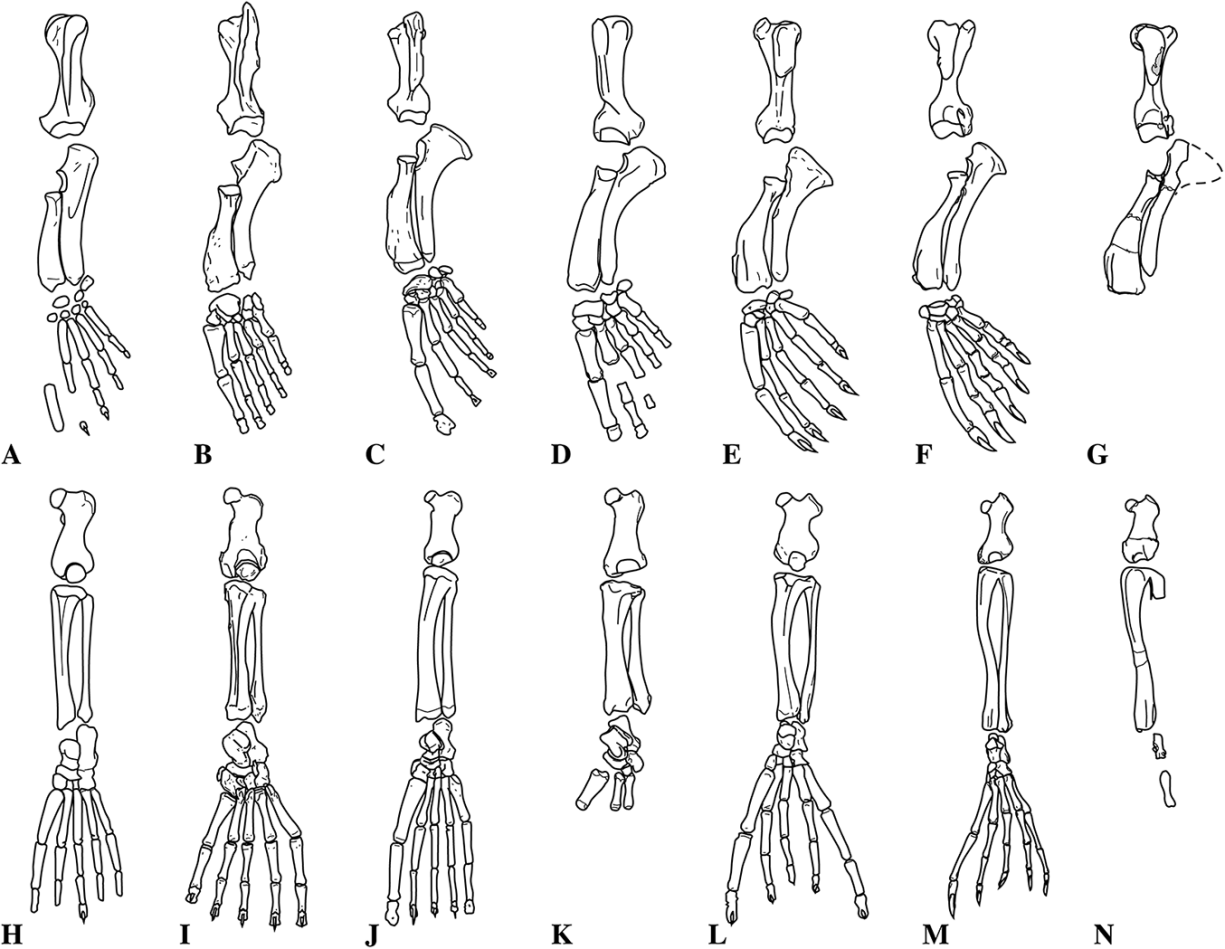
Comparison of pinnipedimorph fore and hindlimbs. Fore (A–G) and hindlimbs (H–N) of selected pinnipedimorphs: *Enaliarctos mealsi* (A, H), *Odobenus rosmarus* (B, I), *Otaria byronia* (C, J), *Allodesmus kernensis* (D, K), *Monachus schauinslandi* (E, L), *Pusa sibirica* (F, M), and *Nanophoca vitulinoides* (G, N). All illustrations rescaled to the same size. Illustrations of *E. mealsi*, *M. schauinslandi*, *O. rosmarus*, and *O. byronia* are modified from Berta, Ray & Wyss (1989) and Berta & Ray (1990).

**Figure 31:**
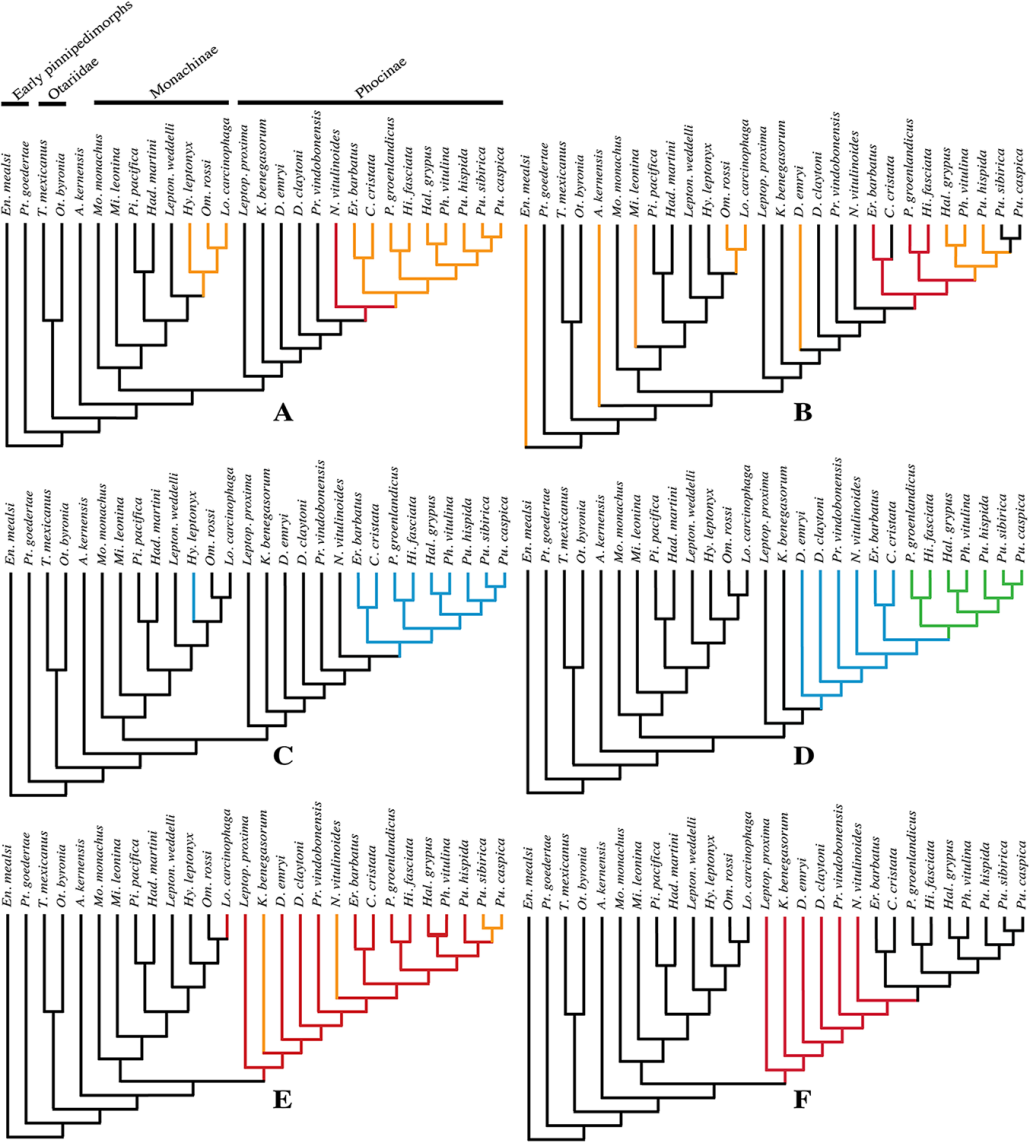
Fore and hindlimb character acquisition and loss in pinnipedimorphs. Selected characters and character states of fore (A–C) and hindlimbs (D–F) in Pinnipedimorpha. (A) Lesser tubercle of humerus lower than head (black), higher than head (orange), or equal in height (red). (B) Greater tubercle of humerus lower than head (black), equal (red), or higher than height head (orange). (C) Deltopectoral crest of humerus limited to the proximal half of the bone (blue) or not limited to the proximal half of the bone (black). (D) Lateral eversion of the ilium weak (black), moderate (blue), or prominent (green). (E) Height of greater trochanter of femur lower than height of head (black), equal (red), or higher than height of head (orange). (F) Suprapatellar fossa of femur absent (black) or present (red).

In *N. vitulinoides*, the greater tubercle of the humerus is at the level to or slightly proximal to the lesser tubercle (Figs. 28 and 29). Muizon (1981) elaborately described the functional implications of a large versus a small greater tubercle (insertion of *musculus supraspinatus* and *musculus infraspinatus*) and lesser tubercle (insertion of *musculus subscapularis*) in extant pinnipeds. Phocidae (except *Monachus* spp.) are characterized by a strongly developed lesser tubercle, exceeding the greater tubercle, while Otariidae have strongly developed greater tubercle, exceeding the height of the lesser tubercle. *N. vitulinoides* exhibits an intermediate state with a moderately well developed lesser tubercle reaching the proximal level of the head and greater tubercle reaching the proximal level of the head or slightly proximal to it, an intermediate state between extant Phocinae and Otariidae + Odobenidae (Figs. 30 and 31). Furbish (2015) noted that a reduced greater tubercle and a reduced lesser tubercle represent ancestral traits among pinnipeds (see *Enaliarctos*, Figs. 30A and 30H). Indeed, the early pinnipedimorph *Enaliarctos* has been inferred to be a hindlimb dominated swimmer, like the Phocidae (Bebej, 2009).

In extant Phocidae, fore flipper propulsion is very limited and the humerus is mostly held along the body while the hind flippers are used for propulsion (Muizon, 1981; Berta & Ray, 1990; Bebej, 2009). Therefore, the use of *musculus subscapularis* to rotate the humerus medially must be neither powerful nor rapid but energy efficient, hence the large effort arm (lesser tubercle) of this third class lever to increase energy efficiency of the action. The spinatus muscles serve to abduct the forearm. The use of fore flippers for aquatic directional (Tarasoff et al., 1972; Muizon, 1981; Berta & Ray, 1990) and braking (Muizon, 1981) purposes in extant Phocidae requires both rapidity and strength (Muizon, 1981). The use of the fore flippers for braking in Phocidae may be questionable. Tarasoff et al. (1972) and Kuhn & Frey (2012) argue that Phocinae (*P. groenlandicus* in Tarasoff et al. (1972) used their fore flippers principally for steering and to a lesser extent for aquatic propulsion and to maintain stability during inactive periods. Neither observed any indication of the use of the fore flippers for braking, contrasting with Muizon (1981). Muizon’s (1981) observations of the active use of fore flippers for aquatic braking by extending them may be related to the sharp turning (steering) at high velocity observed by Kuhn & Frey (2012). While otariids use their fore flippers actively for terrestrial locomotion, the use of fore flippers for terrestrial locomotion is strongly reduced in phocids. For Lobodontini (O’Gorman, 1963) and the phocines *C. cristata*, *E. barbatus*, *H. grypus*, *P. groenlandicus*, *Phoca vitulina*, *Pusa hispida*, and *Pusa sibirica* (Tarasoff et al., 1972; Kuhn & Frey, 2012), different modes of terrestrial locomotion have been observed, depending on the substrate, speed and taxon (O’Gorman, 1963). The three different modes are (1) a “caterpillar-like” (Kuhn & Frey, 2012) motion with vertical undulation of the vertebral column without the use of fore flippers, (2) the aforementioned “caterpillar-like” locomotion in part aided by the fore flippers to pull the body, and (3) a horizontal sinuous locomotion pushing snow posteriorly with both fore and hind flippers. Using the fore flippers to grasp the substrate requires a strongly developed *musculus infraspinatus*, to laterally rotate the humerus (Kuhn & Frey, 2012). Although the reduced size of the greater tubercle reduces the energy efficiency of the *musculus infraspinatus* compared to Otariidae, the strong development of the origin and insertion of the *musculus infraspinatus* still indicates that this muscle is powerful in Phocinae.

The small size of the greater tubercle (not reaching the proximal level of the humeral head) decreases the effort arm of the lever and increases the speed of the abduction of the fore flipper. In Otariidae, the opposite is true: rapid and powerful adduction of the fore flippers during swimming requires rapid action of a large *musculus subscapularis* through a small effort arm (small lesser tubercle). With the fore flippers as the primary organ of propulsion in Otariidae (Muizon, 1981; Berta & Ray, 1990; Bebej, 2009; Kuhn & Frey, 2012), a swift abduction of the fore flipper follows each powerful backward stroke that adducts the limb. In order to do so, the spinatus muscles of Otariidae are aided by a large greater tubercle providing a large effort arm of the lever and, hence, rapid abduction of the humerus.

In *N. vitulinoides*, the relative dimensions of the lesser and greater tubercle are intermediate between the conditions observed in extant Phocidae and Otariidae + Odobenidae. The moderate dimensions of the lesser tubercle (Fig. 31A), the greater tubercle reaching the level of the humeral head proximally (Fig. 31B), and the well-developed insertion for the spinatus muscles in *N. vitulinoides* point toward a moderately powerful and moderately rapid adduction of the humerus, intermediate between extant Phocidae and extant Otariidae, and powerful and moderately rapid abduction of the humerus. Regarding terrestrial and aquatic locomotion, the strong development of a deep pit for the insertion of *musculus infraspinatus* on the greater tubercle points toward a relatively strong development of this muscle in *N. vitulinoides* and, hence, a more frequent use of this fore flipper to crawl on land than extant Phocinae do.

In the water, the morphology of the proximal part of the humerus between Otariidae and Phocidae suggests more frequent use of the fore flipper for aquatic propulsion than extant Phocidae. However, the aforementioned reduced length of the cervical vertebrae does not support any strong resemblance to either the terrestrial or the aquatic locomotion of Otariidae, which use their fore flippers much more profoundly for terrestrial locomotion and aquatic propulsion than Phocidae. A similar condition as in *N. vitulinoides* can be seen in other extinct Phocidae (see, e.g., Muizon, 1981; Koretsky, 2001; Koretsky & Rahmat, 2013; Koretsky, Peters & Rahmat, 2015), showing a gradual increase in relative dimensions of the lesser tubercle through geologic time, as well as a gradual decrease of the relative dimensions of the greater tubercle. The latter can be considered a reversal, because it has been assumed that a reduced greater trochanter is an ancestral pinnipedimorph trait (Furbish, 2015). The lesser tubercle is relatively small in many stem phocines, such as *L. proxima* (Dewaele, Lambert & Louwye, 2017), of intermediate dimensions in other extinct phocines (e.g., *C. maeotica*) and large in other extinct and extant phocines (e.g., *M. pontica*). This points toward a significantly more frequent use of the fore flipper for aquatic propulsion in those extinct phocines than in living phocines, the latter heavily relying on hind flipper propulsion in the water and holding their fore flippers firmly against their bodies during aquatic undulating of the pelvis (Muizon, 1981; Berta & Ray, 1990; Bebej, 2009; Kuhn & Frey, 2012). Compared to other phocines, the large size of the humeral head in *N. vitulinoides* and its rather strong posterior protrusion from the body of the humerus may have allowed an increased mobility of the scapulohumeral joint (see Muizon, 1981).

In *N. vitulinoides*, the deltopectoral crest projects anteriorly only to a moderate extent. In extant pinnipeds, the deltopectoral crest is far better developed in Phocidae than in Otariidae. In extinct pinnipeds and early pinnipedimorphs, the deltopectoral crest is always relatively poorly developed compared to their extant relatives (Fig. 30) (Mitchell, 1966; Repenning & Tedford, 1977; Berta & Ray, 1990). Extant Phocidae make extensive use of the fore flippers for aquatic directional purposes as well as for stability and to a very minor extent for terrestrial locomotion, while Otariidae use their fore flippers predominantly for aquatic propulsion while using their posterior body for steering. Hence, extant Phocidae generally require a powerful but not necessarily rapid extension (*musculus atlantoscapularis* and *musculus humerotrapezius*) and abduction (*musculus deltoideus*) of the humerus (Muizon, 1981). The strongly developed deltopectoral crest provides a long effort arm for the lever of the appropriate muscles, indeed resulting in a powerful but relatively slow extension and abduction of the fore flipper. Fore flipper propulsion in Otariidae, on the other hand, requires rapid strokes and a smaller effort arm for the extension and abduction of the fore flippers. Similarly, the shape of the deltopectoral crest in *N. vitulinoides* can be regarded as functionally intermediate between extant Phocidae and Otariidae, suggesting a combined use of the fore flippers for aquatic direction purposes and propulsion. The degree of anterior projection of the deltopectoral crest in *N. vitulinoides* is intermediate between extant Phocidae and other pinnipeds + early pinnipedimorphs, a hypothesis supported by the phylogenetic analysis (Fig. 31C).

The bicipital groove of the humerus is narrow in *N. vitulinoides*. Among extant Phocidae, the relative dimensions of the bicipital groove take two forms: in Monachinae, the bicipital groove is generally very wide and shallow (wider than deep), while it is rather deep and narrow in extant Phocinae (deeper than wide). Comparing *N. vitulinoides* to extant phocines, the bicipital groove of the former is still proportionally narrower but not particularly deeper than the latter. No muscles originate from or insert on the bicipital groove (Howell, 1929; Muizon, 1981). However, this groove serves to guide *musculus biceps brachii*. Moreover, some monachines (e.g., *L. carcinophaga*) have a transverse bar within the bicipital groove to act as a pulley and increase the effort arm for *musculus biceps brachii*. Muizon (1981) discusses that *musculus biceps brachii* is predominantly used for braking in Phocidae. A wider bicipital groove accommodates a larger *musculus biceps brachii* and a transverse bar further aids in braking by increasing the effort arm, hence increasing strength of the muscle. Contrastingly, a narrow bicipital groove implies a reduced use of the fore flipper for braking or a less powerful brake. The proportionally narrow bicipital groove in *N. vitulinoides*, which is deeper than wide, implies the existence of a relatively small *musculus biceps brachii* and, thus, most likely a subordinate flexion of the elbow and subordinate use of the fore flipper for braking in *N. vitulinoides*.

The reduced olecranon fossa on the humerus limits the extension of the elbow during swimming. This last observation supports the assumption that the fore flipper could not be used for propulsion in *N. vitulinoides* as extensively as in Otariidae, in which the olecranon fossa is much deeper (Muizon, 1981).

In comparison with other phocines, the distal epiphysis of the humerus of *N. vitulinoides* bears a similarly well-developed lateral epicondylar crest, but a rather weakly developed medial epicondyle. When comparing the medial prominence of the medial epicondyle to the mediolateral width of the trochlea + medial epicondyle in posterior view, the ratio of the medial epicondyle versus the trochlea + medial epicondyle is equal to or smaller than 0.25. The *musculus pronator teres* and *musculus supinator* have their origins on the medial and lateral epicondyles, respectively, and most manual flexors and extensors have their origin on the medial and lateral epicondyle, respectively, as well (Howell, 1929; Bryden, 1971; Piérard, 1971; Piérard & Bisaillon, 1975; Muizon, 1981; Evans & de Lahunta, 2013). Hence, in *N. vitulinoides musculus supinator* and the extensors were probably well developed while the actions of the flexors and *musculus pronator teres* were less intense than it is in closely related extant phocines. The action of *musculus supinator* will be treated below. This implied decreased pronation of the fore flipper and decreased use of the manual flexors. The latter allows assuming that pronation of the fore flipper probably did not play an important role in providing forward thrust during aquatic locomotion. On the other hand, an extended wrist joint and extended digits (manual extensors) enabled the animal to push back a larger amount of water with each stroke, but also may have facilitated changes in direction during swimming, because of the larger surface the extended wrist and digits provide. This points toward a roughly similar use of the wrist during aquatic locomotion (propulsion and direction changes) to extant Phocidae.

##### Ulna

On the ulna of *N. vitulinoides*, the proximal margin of the olecranon process is oriented more perpendicular to the long axis of the ulna than it is in other phocines. The presumably powerful *musculus triceps brachii* inserts on the proximal margin of the olecranon. The triceps muscle is one of the few first class levers within the vertebrate body, with the elbow joint acting as the fulcrum and the load (forearm) and effort (insertion of triceps on olecranon of ulna) on opposite sides of it. Compared to other phocines, the increased length of the effort arm at the olecranon in *N. vitulinoides* implies a more powerful action of the *musculus triceps brachii* and thus a stronger but slower extension of the elbow. This would have enabled *N. vitulinoides* to perform powerful propulsive strokes with its fore flippers. In contrast, during terrestrial locomotion, the action of the *musculus triceps brachii* is small: when gripping the substratum with the fore flippers, the latters flex during the forward projection of the body (Thewissen & Taylor, 2007). Therefore, a stronger development of the *musculus biceps brachii* may also have enabled greater terrestrial mobility in *N. vitulinoides* compared to many extant phocids. However, this contradicts the apparently poor development of *musculus biceps brachii*.

##### Radius

The insertion surfaces for *musculus supinator*, *musculus pronator teres*, and *musculus brachioradialis* correspond to that of other Phocidae (except extant Lobodontini) and Otariidae: *musculus supinator* and *musculus brachioradialis* were most likely strongly developed and *musculus pronator teres* was relatively weakly developed in *N. vitulinoides* (see also Muizon, 1981). These similarities of *N. vitulinoides* with most other phocids suggest that supination of the forearm was relatively strong, whereas pronation was relatively weak. The relatively proximal position of the insertion area for *musculus brachioradialis* indicates a shortened load arm and more powerful use of this muscle in comparison to extant Phocidae. Hence, supination of the forearm is relatively strong, while pronation is relatively weak. This points toward a roughly similar use of the fore flipper for steering and braking as extant Phocidae (except extant Lobodontini) and Otariidae. This does not contradict the presumed improved use of the fore flipper for propulsion, as stated above. Neither does it a priori contradict the relatively modest use of the fore flipper for aquatic braking proposed in this study. Kuhn & Frey (2012) implicitly state that active braking in phocids may have been very limited and that the animals just cease propulsion and use drag or turn around.

#### Pelvic girdle

##### Sacrum

**Figure 32:**
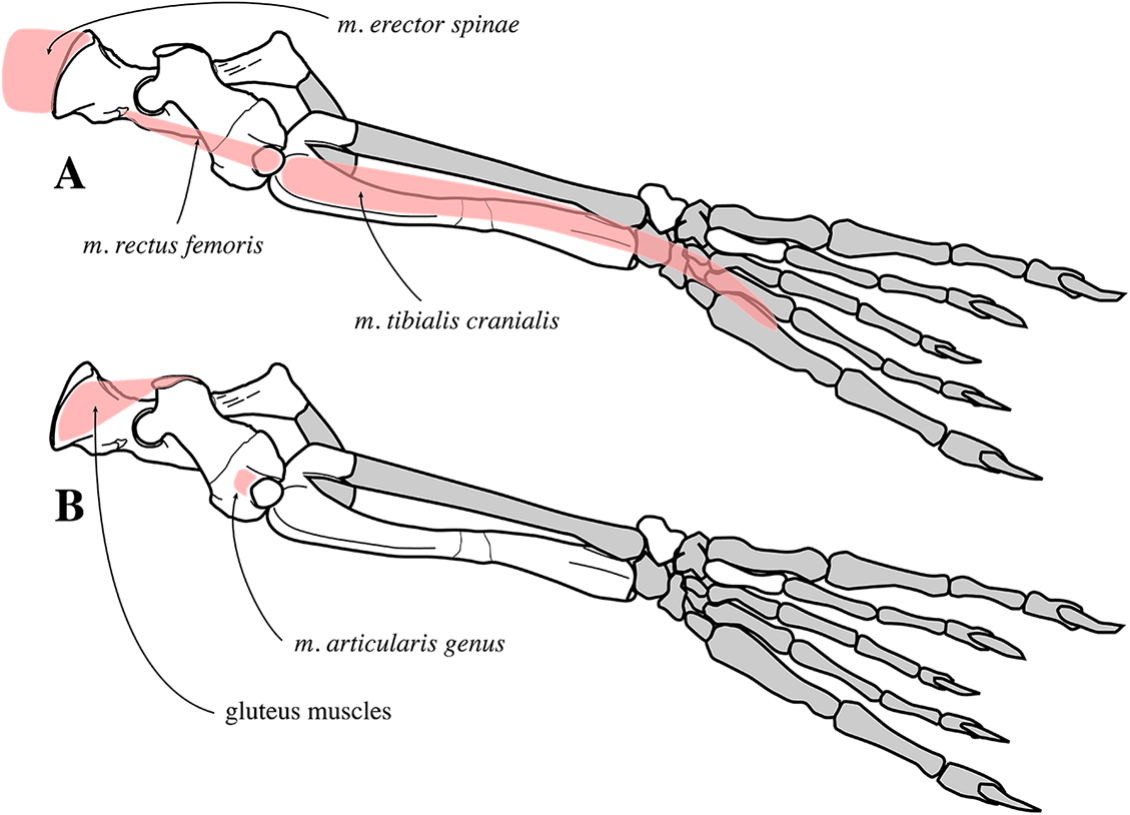
Hindlimb musculature of *Nanophoca vitulinoides* in anterior view. The origin and insertion of selected muscles of the hindlimb that are visible in anterior view. Muscles indicated in pink. Missing bones or bone parts of *Nanophoca vitulinoides* indicated in gray. This illustration focuses on the visualization of the origin and insertion of different muscles. Hence, the actual shape of the muscles may differ from this illustration. Note that *musculus erector spinae* (A) has its origin on the lumbar vertebrae and is not illustrated and that *musculus articularis genus* (B) inserts on soft tissues and is also not indicated in this illustration.

**Figure 33:**
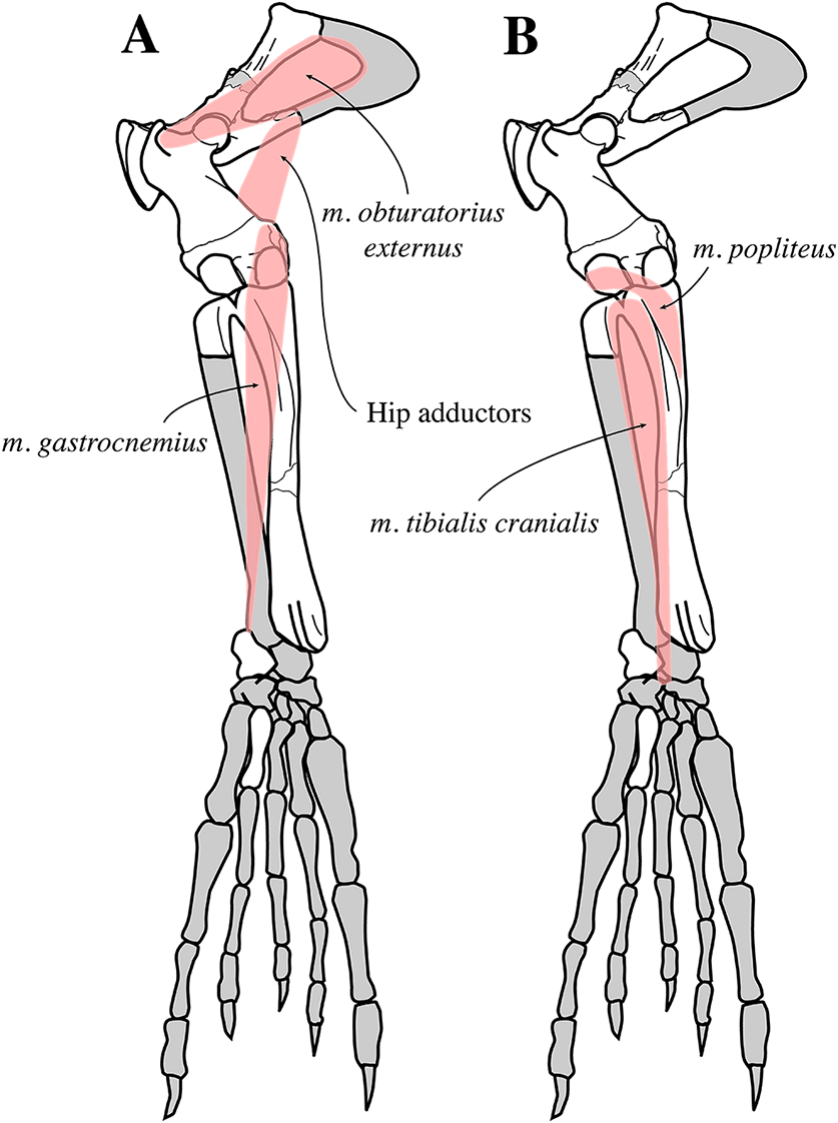
Hindlimb musculature of *Nanophoca vitulinoides* in posterior view. The origin and insertion of selected muscles of the hindlimb that are visible in posterior view. Muscles indicated in pink. Missing bones or bone parts of *Nanophoca vitulinoides* indicated in gray. This illustration focuses on the visualization of the origin and insertion of different muscles. Hence, the actual shape of the muscles may differ noticeably from this illustration.

Strongly developed sacral wings, joined by their strong ventral projection, further distinguish *N. vitulinoides* from extant Phocidae (Figs. 32 and 33). Having large sacral wings is an apomorphic trait shared by the Phocidae as it is not found in other Carnivora. Muizon (1981) noted that, in extant Phocidae, these sacral wings are consistently larger in Phocinae than in Monachinae. As noted earlier in this paper, our preliminary measurements do not support such a clear distinction between both subfamilies (see also Dewaele, Lambert & Louwye, 2017). Nevertheless, the sacral wings of *N. vitulinoides* are relatively large, reaching the upper portion of the range of observed ratios, hence of typical phocine disposition. Muizon (1981) proposed that the *musculus erector spinae* originated on the anterior surfaces of the sacral wings of *A. longirostris*. Contrastingly, Howell (1929) found that the *musculus erector spinae* originated on the anterior border of the iliac crest in the ringed seal, *Pusa hispida*. However, Howell (1929) noted the strong development of the *erector spinae* muscles in *Pusa hispida* compared to the Californian sea lion, *Zalophus californianus*, the latter having much smaller sacral wings (Howell, 1929); King (1964) observed the strong development of *musculus iliocostalis lumborum* (one of the erector spinae muscles) in phocids, and Bryden (1971) observed the insertion of the *musculus longissimus lumborum* (also one of the erector spinae muscles) in the southern elephant seal, *Mirounga leonina*. Hence, the development of the sacral wings may tentatively be linked to the development of the powerful erector spinae muscles and the ability to flex, extend and rotate the dorsum during horizontal aquatic pelvic undulations. However, iliac wings that are strongly everted laterally reduce the effort arm of this third class lever. With the lumbar region acting as the fulcrum, the effort arm (insertion area on sacrum) is located between the fulcrum and the load arm (hindlimbs and pelvis). The decreased length of the effort arm implies an increased speed and slightly reduced power of the action. Hence, having both a strongly enlarged insertion area for the erector spinae muscles and the reduced effort arm of the lever, both aquatic and terrestrial flexion and extension of the pelvis were powerful and fast in *N. vitulinoides*. On the other hand, it can be tentatively assumed that the strong anterior projection of the sacral wings in *N. vitulinoides* strongly physically reduced the amplitude of the lateral oscillations of the pelvis compared to extant seals. In addition, the sacral wings generally serve to attach the ventral sacroiliac ligament and to connect the sacrum with the innominate (for the domestic dog, see Evans & de Lahunta, 2013). Larger sacral wings may have supported a larger ventral sacroiliac ligament and may, hence, tentatively correlate to a stronger contact between sacrum and innominate. Such a stronger contact may be required to keep the pelvis stable during the quick and powerful use of it during terrestrial locomotion (vertical undulation) and aquatic locomotion (horizontal movement).

The spinous processes of the sacrum are high and fused in *N. vitulinoides*. No such condition has been observed in any extant species of Phocidae. Hence, comparison with extant relatives is excluded. However, the extant sea otter, *Enhydra lutris* Fleming, 1828 has high but separate sacral spinous processes (see Howard, 1975) and the extinct early pinnipedimorph genus *Enaliarctos* Mitchell & Tedford, 1973 has fused sacral spinous processes (Berta & Ray, 1990). Inferences on the musculature of different taxa are not straightforward. Evans & de Lahunta (2013) showed that parts of *musculus multifidus*, a fixator of the vertebral column inserts on the lateral side of the separate sacral spinous processes of the domestic dog. For *Enhydra lutris*, Howard (1975) noted that the origin of *musculus piriformis*, a rotator of the thigh has its origin on the separate, first sacral spinous process. For *Enaliarctos* spp., Berta & Ray (1990) did not infer the musculature of the sacrum.

Because it has been shown that *musculus piriformis* does not originate on the sacral spinous processes in Phocidae (Howell, 1929; Muizon, 1981), we tentatively assume that the fused and dorsally elongated sacral spinous processes provided a physically stronger insertion area for the multifidus muscles (origin on lumbar vertebrae), hence allowing to assume that the multifidus muscles in *N. vitulinoides* was more strongly developed than in extant Phocidae. This allows strong fixation of the vertebral column at the level of the lumbar region and the pelvis in *N. vitulinoides*. Hence, it can tentatively be assumed that the rapid and powerful motion of the pelvis of *N. vitulinoides* and lumbar vertebrae (through the action of the *erector spinae* muscles) during terrestrial and aquatic locomotion required profound stabilization through the *multifidus* muscles.

##### Innominate

The gluteal fossa on the ilium is relatively weakly developed (i.e., little concave) in *N. vitulinoides*. Among extant Phocidae, Monachinae lack a gluteal fossa, while extant Phocinae have a well-developed fossa, with the exception of *C. cristata* and *E. barbatus* displaying a very shallow fossa. Compared to pinnipedimorphs in general, the gluteal fossa of *N. vitulinoides* is still well developed. A weakly developed gluteal fossa implies less powerful gluteus muscles, and hence a relatively weak external rotation and extension of the hip joint.

On the other hand, the strong development of the lateral eversion of the innominate in extinct and extant phocines expands the insertion area for the erector spinae muscles anterior to it (Fig. 31D). Hence, increased lateral eversion of the innominate, in Phocidae in general, but even more in Phocinae, implies more strongly developed erector spinae muscles in these taxa and, thus, increasedly powerful lateral movements of the pelvis for aquatic locomotion (see, Berta, Sumich & Kovacs, 2015; Kuhn & Frey, 2012). As shown in Fig. 31D, earlier branching Phocinae had slightly less laterally everted innominates than later branching taxa, an observation suggesting that Phocinae gradually adapted to aquatic pelvic locomotion through their evolutionary history.

The femur of *N. vitulinoides* has a greater trochanter that is proximally raised over the femoral head, increasing the effort distance to the fulcrum (coxo-femoral joint) and hence increasing power and energy efficiency of the extension of the hip and lateral rotation of the thigh. Among extinct Phocinae, the moderately strong development of the gluteal fossa, the lateral eversion of the ilium, and the high greater trochanter reaching proximal to the level of the head are not uncommon (Figs. 31D and 31E). However, an enlarged greater trochanter physically decreases the amplitude of the actions on the coxo-femoral joint (Muizon, 1981). An energy-efficient extension and external rotation of the hip joint during aquatic locomotion helps in preserving energy for the powerful flexion in *N. vitulinoides* (i.e., swim stroke), when water is pushed backwards to produce the aquatic thrust (see Muizon, 1981; Kuhn & Frey, 2012).

*Nanophoca vitulinoides* also has a strongly marked fossa on the dorsolateral side of the anterior portion of the iliac branch of the pubis. This fossa serves as an origin for part of *musculus obturatorius externus*, an adductor of the thigh, and it is generally either absent or only weakly developed in extant Phocidae. Although the strong development of this fossa in *N. vitulinoides* cannot be easily compared to extant Phocidae, it can be assumed that *musculus obturatorius externus* was a relatively powerful muscle in *N. vitulinoides*, providing powerful adduction of the thigh. A lateral adduction of the thigh has been predicted above when assuming a powerful “swimming stroke” of the hind flipper opposing the energy-efficient extension and abduction of the hind flipper at the coxo-femoral joint.

A weak depression is present anteroventral to the acetabulum of *N. vitulinoides*. A similar facet is present in extant Phocinae, but missing in extant Monachinae; in the former it marks the origin of *musculus rectus femoris* on the ilium, which is relatively strongly developed in extant Phocinae, compared to extant Monachinae, and acts to extend the knee joint. Hence, a relatively deep origin of *musculus rectus femoris* implies that the extension of the knee joint is more powerful in extant Phocinae and *N. vitulinoides* than in Monachinae.

*Nanophoca vitulinoides* also has the unique characteristic of a hook-like strongly developed ischiatic spine. Koretsky & Peters (2008) describe an innominate attributed to *B. neerlandica* with a well-developed ischiatic spine. However, this innominate had been found isolated from other specimens of *B. neerlandica* and cannot be assigned to the species in the absence of an innominate associated with other bones. Hence, no other extant or extinct phocid species has an ischiatic spine similar to *N. vitulinoides* and most taxa only have a marked rugosity or a blunt process on the ischium (L. Dewaele, 2015, personal observation). The skeletally archaic *P. rousseaui* has a sharp, elongated, ridge-like tubercle (Dewaele, Lambert & Louwye, 2017). Based on the analogy to the domestic dog (Evans & de Lahunta, 2013), this ischiatic spine may have served to attach and guide the dorsal sacroiliac ligament. Hence, strong development of this tubercle in *N. vitulinoides* may indicate a strong ligament uniting the sacrum and the innominate. This interpretation is in accordance with the presumed large ventral sacroiliac ligament proposed in this species based on the enlargement of the sacral wings, as compared to most extant phocids (see above).

##### Femur

The trochanteric fossa on the femur is proportionally deep in *N. vitulinoides* as well as in other extinct phocines (see Koretsky, 2001; L. Dewaele, 2015, personal observation) and the extant *Pusa* species compared to other, extant, phocines, whereas it is either absent or strongly reduced in extant and extinct Monachinae. Because the trochanteric fossa serves as the insertion area for *musculus obturatorius externus*, the great depth of the fossa in *Praepusa* further supports the hypothetic presence of a powerful *musculus obturatorius externus* as proposed above.

The femoral head of *N. vitulinoides* forms much more than a hemisphere (i.e., it is more spherical), with a clearly outlined neck. The width of the neck is less than 90% the height of the head (Table S6). In extant Phocidae, the shape and dimensions of the femoral head differ slightly among different species, with varying degrees of sphericity. In extant Monachinae the femoral head is hemispherical, with an ill-defined neck, while in extant Phocinae this head forms more than a hemisphere and the neck is better pronounced. Muizon (1981) suggested that this differentiation between extant Monachinae and Phocinae indicates greater amplitude in the motion of the coxo-femoral joint in Phocinae than in Monachinae. Similarities in the shape of the femoral head and neck between extant Phocinae and *N. vitulinoides* allows to assume that the latter showed a rather great amplitude in the motion of the coxo-femoral joint as well. This assumption contrasts with the presumed reduced mobility of the coxo-femoral joint due to the enlarged greater trochanter in *N. vitulinoides* (see above).

Moreover, most specimens of *N. vitulinoides* have a noticeable pit on the femoral head. Although this feature is only very weakly developed in extant Phocidae, if developed at all, the primitive pinnipedimorph *Enaliarctos* spp. also display a strongly marked pit on the femoral head, interpreted as an attachment site for the teres femoris ligament (Berta & Ray, 1990). The authors suggest that this marked pit—and the associated strong development of the teres femoris ligament—in *Enaliarctos* spp. indicates strong fixation of the femoral head during terrestrial locomotion as well as a relatively high degree of maneuverability of the hindlimb on land for *Enaliarctos* spp., compared to extant Phocidae. A similar reasoning may be applied to *N. vitulinoides*, with a greater terrestrial maneuverability of the hindlimb relative to extant Phocidae. Extending this reasoning to *N. vitulinoides* and many other extinct phocines, such as *Praepusa vindobonensis*, it can be assumed that these taxa spent much more time on land than most extant phocids.

A prominent adductor tubercle located on the medial epicondyle of the femur, as described in *N. vitulinoides* is not uncommon among extant and extinct Phocinae (e.g., *C. maeotica* (Koretsky, 2001), *H. fasciata*, and *P. groenlandicus*). Nevertheless, its strong development in *N. vitulinoides* corroborates the hypothesis of a strong development of the hip adductor muscles in the latter and, hence, the probable great performance of powerful backwards swimming strokes of the hind flippers.

Just proximal to the medial condyle of the femur of *N. vitulinoides*, a small but clearly visible ridge contrasts with the indistinct elevation observed in extant Phocidae. In the latter, this area is the site of origin of the lateral and medial heads of *musculus gastrocnemius*, a flexor of the knee and plantar-flexor of the ankle. Thus, the ridge observed in *N. vitulinoides* is interpreted as a marked separation between both muscle heads, suggesting their strong development. The lateral side of the lateral condyle of the femur of *N. vitulinoides* bears a deep pit, approximately 1 mm deep. This pit serves as the origin of *musculus popliteus*, a flexor and lateral rotator of the knee joint. Hence, with a powerful *musculus gastrocnemius* and a powerful *musculus popliteus*, flexion of the knee may have been more intensively performed in *N. vitulinoides* than in extant phocids. This hypothesis corroborates the foregoing assumption that the hindlimb is highly involved in aquatic propulsion in this extinct species, while in extant Phocidae the aquatic propulsion is mainly performed by the lumbar and pelvic regions, with the hind flippers being used more passively (Kuhn & Frey, 2012). Yet, Tarasoff et al. (1972) observed rotation of the hindlimb and knee of *P. groenlandicus* during aquatic locomotion. The increasingly powerful lateral rotation of the knee in *N. vitulinoides* may also be linked to a more extensive use of the hind flippers during terrestrial locomotion, compared to extant Phocidae. In extant phocids, Antarctic seals have been observed to use their hind flippers for pushing away snow during “sinuous” locomotion on firm snow and ice, but not on rocky and sandy beaches, where all phocids appear to perform an undulatory terrestrial locomotion with only very limited aid of the hind flippers (O’Gorman, 1963). Whereas the patellar facet is a deep concave surface in *N. vitulinoides*, with a marked suprapatellar fossa proximal to it, extant Phocidae, lack a pronounced suprapatellar fossa (Fig. 31F). However, the patellar facet attains different shapes in different taxa: extant Phocinae have slightly concave suprapatellar fossae, while the patellar fossae in extant Monachinae is only very faintly concave, if not flat. According to Muizon (1981), this difference may point toward an increased mobility of the knee joint and thus more frequent use of the knee in extant Phocinae compared to Monachinae. As the patellar facet is more strongly concave in *N. vitulinoides* than in extant Phocinae, an even greater mobility of the knee joint can be proposed in *N. vitulinoides*. Contrasting with the very weakly developed (i.e., very little concave or flat) suprapatellar fossa in extant Phocinae, a number of extinct Phocinae have a marked fossa (e.g., *Phocanella pumila, N. vitulinoides*, and *P. rousseaui*). Although the functional significance of such a suprapatellar fossa for both aquatic and terrestrial locomotion in these extinct seals is difficult to elucidate, comparisons with the domestic dog (Evans & de Lahunta, 2013) suggests that the site serves as the origin of *musculus articularis genus*, an extensor of the so-called stifle joint, i.e., the knee joint. The depth of the suprapatellar fossa also increases the accommodation space for an increased flexibility of the knee joint in *N. vitulinoides*, further supporting the presumed enhanced mobility and flexibility of the knee joint in this species over extant Phocinae.

##### Tibia

The well-defined fossa marking the proximal portion of the posterior side of the tibia of *Praepusa vitulinoides* is absent in extant Phocidae. However, some extant species do have a slightly concave surface at the site (e.g., *Pusa* spp.), serving for the insertion of *musculus popliteus*. This supports the aforementioned presumed strong development of *musculus popliteus*—a flexor and rotator of the knee—in *N. vitulinoides*. In *Enaliarctos* spp., Berta & Ray (1990) assume that the possibility of powerful rotation of the hindlimb suggests active use of the hind flippers during terrestrial locomotion. Analogous to *Enaliarctos*, the possibility of a powerful rotation of the hindlimb of *N. vitulinoides* may also suggest active use of the species’ hind flippers during terrestrial locomotion.

#### Overall functional anatomy

The functional anatomical interpretations of the axial skeleton and the pectoral and pelvic girdles of *N. vitulinoides* indicate a lifestyle that markedly differs from that of extant Phocidae. Although the axial skeleton of *N. vitulinoides* does not differ strongly from that in extant species, the anatomy of both the pectoral and the pelvic girdles in the former points toward and increased mobility of the fore and hind flippers, compared to all extant phocids. Hence, although it would be presumptuous to draw firm conclusions about the locomotion strategies in *N. vitulinoides*, it can be proposed that it used more actively the fore and hind flippers during both aquatic and terrestrial locomotion, compared to extant phocid species. Nevertheless, the overall postcranial anatomy of *N. vitulinoides* is typically phocine, and the inferred more active use of its fore and hind flippers should be considered with care, certainly not implying a terrestrial locomotion mode as performed by Otariidae. *N. vitulinoides* presumably rather used its fore and hind flippers for grasping and crawling on the substratum. In the water, *N. vitulinoides* most likely relied more on its fore flippers for swimming than extant Phocidae (that use more predominantly their lumbus and pelvis to perform in pelvic oscillations). An alternative hypothesis that may explain the more active use of fore flippers in *N. vitulinoides* than in living Phocidae may relate to a more prominent use during prey capture and manipulation. As shown by Hocking et al. (2017), feeding strategies of aquatic mammals follow an evolutionary sequence, going from terrestrial feeding, via semi-aquatic feeding, raptorial feeding, suction feeding, and suction filter feeding, to ram filter feeding. While the extant *H. leptonyx* is capable of performing semi-aquatic feeding, raptorial feeding, suction feeding, and suction filter feeding, it can be argued that more ancient phocids may have been restricted to feeding strategies that fall early in the evolutionary feeding sequence (semi-aquatic and raptorial feeding). Consequently, the role of the forelimbs in prey capture, prey manipulation, and prey processing may have been more predominant than in extant Phocidae. However, this hypothesis remains difficult to test in the absence of more complete specimens of *N. vitulinoides* and given the small body of scientific studies of feeding strategies in Phocidae (see Hocking, Evans & Fitzgerald, 2013). Table 2 presents the presumable relative importance of selected muscles of *N. vitulinoides* in relation to extant relatives (including Phocidae as well as Otariidae). Overall, it can be assumed that *N. vitulinoides* was functionally very close to extant Phocidae but retained a certain number of anatomical characteristics that indicate an increased use of the fore flipper for aquatic propulsion and a more prominent use of both fore and hind flippers during terrestrial locomotion.

**Table 2:** Systematic overview of skeletal differences between *Nanophoca vitulinoides* and extant Phocinae. Skeletal differences between *Nanophoca vitulinoides* and extant Phocinae are provided, together with their myological and locomotive implications.

Bone	Character	Development compared to extant Phocinae	Muscle	Implications for *Nanophoca vitulinoides*
Cervical vertebra	Small with small spinous process^†^	=	Epaxial muscles (*musculus multifidus lumborum*, *musculus longisimus thoracics*)	Limited use of fore flippers during aquatic and terrestrial locomotion
Lumbar vertebra	Robust with well developed spinous process*^†^	=	Hypaxial muscles (*musculus quadratus lumborum*, *musculus longissimus thoracis*, *musculus iliocaudalis*)	Strong horizontal ambulation posterior end body during aquatic locomotion
Humerus	Height greater tubercle (insertion spinatus muscles)*^†^	+	*Musculus supraspinatus*, *musculus infraspinatus*	Decreased speed but increased power abduction foreflipper
	Height lesser tubercle*^†^	−	*Musculus subscapularis*	Increased speed and decreased power medial rotation and adduction foreflipper
	Anterior projection deltopectoral crest*^†^	−	*Musculus atlantoscapularis* (extension), *musculus humerotrapezius* (extension), *musculus deltoideus* (abduction)	Increased speed and decreased power extension and abduction foreflipper
	Width bicipital groove*	−	*Musculus biceps brachii*	Weakly developed flexion of foreflipper
	Development lateral epicondylar crest*	=	Most manual extensors and *musculus pronator teres*	Similarly frequent use of manual extensors and pronation of fore flipper
	Development medial epicondyle*	−	Most manual flexors	Less intense use of manual flexors
Ulna	Development olecranon process*^†^	+	*Musculus. triceps brachii*	More powerful extension fore flipper
Radius	Insertion surfaces *musculus supinator*, *musculus pronator teres*, and musculus brachioradialis*	=	*Musculus supinator*, *musculus pronator teres*, *musculus brachioradialis*	Supination and pronation of foreflipper about equally strong and weak, respectively, as in extant Phocinae
	Proximal position of insertion surface musculsu brachioradialis*	+	*Musculus brachioradialis*	Increased power (and reduced speed of) supination of the foreflipper
Sacrum	Size sacral wings*^†^	=/+	*musculus erector spinae*, ventral sacroiliac ligament	Flexion, extension and rotation of dorsum during horizontal aquatic pelvic undulations with firm contact of sacrum to innominate, similar to extant Phocinae
	Anterior projection sacral wings	+	*Musculus erector spinae*	Increased speed of flexion, extension and rotation of dorsum during aquatic locomotion but reduced amplitude
	Spinous processes	+ (incl. fusion)	?*Musculus multifidus*	?Increased stability lumbus during pelvic oscillations
Innominate	Depth gluteal fossa*^†^	=/−	Gluteus muscles	Relatively weak external rotation and extension hip joint (contra greater trochanter femur, see below)
	Development origin surface musculus obturatorius externus*	+	*Musculus obturatorius externus*	Relatively powerful adduction thigh
	Development origin fossa *musculus rectus femoris*	=	*Musculus rectus femoris*	Relatively strong extension knee joint, as in extant Phocinae
	Hook-like ischiatic spine	N/A	Dorsal sacroiliac ligament	Strong contact innominate and sacrum
Femur	Height greater trochanter*	+	Gluteus muscles	Relatively strong external rotation and extension hip joint (contra gluteal fossa ilium, see above)
	Depth trochanteric fossa*	=/+	*Musculus obturatorius externus*	Relatively powerful adduction thigh
	Sphericity femoral head (dimensions neck compared to head)*	+ (−)	N/A	Increased mobility coxo-femoral joint
	Development pit femoral head*	+	Teres major ligament	Increased fixation femoral head during terrestrial locomotion and increased maneuverability
	Adductor tubercle medial epicondyle	+	Hip adductors	Powerful adduction hip
	Ridge above medial condyle	N/A	*Musculus gastrocnemius*	?More powerful flexion knee and foot
	Pit on lateral side lateral condyle	+	*Musculus popliteus*	More powerful flexion and lateral rotation knee joint
	Presence suprapatellar fossa	N/A	?*Musculus articularis genus*	?Powerful extension knee joint and providing space for the extension of the knee joint
Tibia	Concavity proximal portion posterior margin of tibia	+	*Musculus popliteus*	More powerful flexion and lateral rotation knee joint

**Notes:**

For the comparison with extant Phocinae: “=” approximately equal, “+” better developed, “−” less developed. Muizon (1981) was the first to discuss the functional anatomy of the axial and appendicular skeleton of Phocidae.

In the “Character” column, an asterisk (*) indicates referral to Muizon (1981), a cross (^†^) indicates referral to Berta, Sumich & Kovacs (2015; and references therein), while no sign indicates an interpretation reported here for the first time. These observations may have been observed in other publications as well, but without connecting character observations with musculature implications.

The middle Miocene was a time of increased temperatures and with much less sea ice in the Northern Hemisphere (MMCO; e.g., Miller & Fairbanks, 1983; Thiede et al., 2011). Hence, in the absence of much sea ice, *N. vitulinoides* may have used its hind flippers during undulatory terrestrial locomotion or may even have employed modes of terrestrial locomotion not observed in extant phocids with a more active use of both fore and hind flippers, rendering it practically impossible to determine the precise cycle of aquatic and terrestrial locomotion in the species. Moreover, an extremely osteosclerotic inner bone structure suggests an ecology that may differ from extant seals (L. Dewaele, 2015, personal observation; work in progress).

### Implications of the new phylogenetic analysis

The extinct Phocinae and Devinophocinae included in the current analysis all return as stem phocines. The phylogenetic relationships among the extant Phocinae in the crown group of the most parsimonious tree (Fig. 25) corresponds relatively well with previously published trees (Bininda-Emonds & Russell, 1996; Higdon et al., 2007; Fulton & Strobeck, 2010), with *C. cristata* and *E. barbatus* found as the first two extant members of the subfamily to branch off. Also in our most parsimonious tree, *H. fasciata* and *P. groenlandicus* group together as the Histriophocini c, 1955. *H. grypus* and *Phoca vitulina* group together in a clade that is sister group to *Pusa*.

Given the relatively short calculated time interval between the divergence of Monachinae and Phocinae and the divergence of crown Phocinae (Higdon et al., 2007), it appears that the Phocinae witnessed rapid diversification during their early evolutionary history, as indicated by the high number of stem phocines. Because no middle Miocene phocines other than *L. proxima* are known from the east coast of North America, and because the diversity of middle and late Miocene Phocinae is high in the North Sea Basin and the Paratethys (Van Beneden, 1876, 1877; Koretsky, 2001; Koretsky & Peters, 2008; Dewaele, Lambert & Louwye, 2017), it can be argued that crown Phocinae originated in Europe during the middle to early late Miocene. Although the current analysis is the first to determine the phylogenetic position of *N. vitulinoides* as the last stem phocine to branch off before the crown group (Fig. 25), other extinct Phocinae considered in the current phylogenetic study have been analysed previously. An analysis by Koretsky (2001) included *L. proxima* (as *Leptophoca lenis*) and *Praepusa vindobonensis*, and returned both species as stem phocines: lineages to extant Phocinae appear to branch off before *L. proxima* and *Praepusa vindobonensis*, introducing long ghost lineages for all extant phocine taxa included. A more recent phylogenetic analysis by Koretsky & Rahmat (2013) shows different results, but *L. proxima* was also recovered as a stem phocine. A more recent phylogenetic study by Berta et al. (2015) considered *L. proxima* a stem monachine, instead of a stem phocine. However, Berta et al. (2015) explicitly doubted this outcome, which is indeed supported by the comprehensive phylogenetic analysis by Dewaele, Lambert & Louwye (2017), which returned both *L. proxima* and *K. benegasorum* as stem phocines, forming a polytomy with crown phocines. The only two other previous phylogenetic analysis including *K. benegasorum* were carried out by Cozzuol (2001) and Dewaele, Lambert & Louwye (2017), and considered *K. benegasorum* and *L. proxima* as stem phocines. The phylogenetic position of the Devinophocinae was examined by Koretsky & Holec (2002), Koretsky & Rahmat (2013), and Koretsky & Rahmat, 2015; in these analyses they returned as a distinct subfamily forming a polytomy with Phocinae and Monachinae (and Cystophorinae). Therefore, the nesting in our analysis of both devinophocine species among stem Phocinae allows questioning the validity of the Devinophocinae as a separate subfamily. We argue that the Devinophocinae subfamily may indeed be a junior synonym to the Phocinae, but a detailed reinvestigation of *Devinophoca claytoni* and *Devinophoca emryi* is beyond the scope of the current study.

## Conclusion

Originally considered to belong to the genus *Phoca*, the generic assignment of the extinct North Sea species “*Phoca*” *vitulinoides* has recently been contested (Koretsky & Ray, 2008). With the addition of new specimens, the study presented here assigns the species to the new genus *Nanophoca*. New biostratigraphic and lithostratigraphic data point to a middle to early late Miocene age (late Langhian to late Serravallian or possible early Tortonian) for this species, although a younger age cannot be ruled out for a few specimens. Our phylogenetic analysis suggests (1) that all included extinct Phocinae, *Devinophoca*, *K. benegasorum*, *L. proxima*, *N. vitulinoides*, and *Praepusa vindobonensis*, are stem Phocinae, and (2) that *N. vitulinoides* is the sister taxon to crown Phocinae. This points toward a strong early diversification of stem Phocinae prior to the evolution of the crown group.

Although aquatic and terrestrial locomotion strategies of *N. vitulinoides* were clearly reminiscent of that of extant phocids, the skeletal anatomy of the species and a comparison with modern pinnipeds point toward an increased use of the fore flippers and an enhanced flexibility of the hind flipper. We argue that *N. vitulinoides* is functionally intermediate between extant Phocidae and a hypothetical terrestrial ancestor, and that it had an increased terrestrial maneuverability and enhanced contribution of the fore flipper during aquatic locomotion compared to extant Phocinae. The increased mobility and strength of the fore flipper in *N. vitulinoides* may alternatively be correlated to an increased use of the paws for prey capture, manipulation, and processing compared to extant phocids, but this remains difficult to test. Body length estimates of *N. vitulinoides* indicate that the species reached an overall length averaging around one meter, making it one of the smallest known pinnipeds.

## Supplemental Information

10.7717/peerj.3316/supp-1 Supplemental Information 1 Phylogenetic matrix including *Praepusa magyaricus* and *Praepusa pannonica*.

10.7717/peerj.3316/supp-2 Supplemental Information 2 Supplemental Information to the reappraisal of *Nanophoca vitulinoides*.

## Institutional Abbreviations

IRSNB: Institut Royal des Sciences Naturelles de Belgique (“M” representing type and figured specimens from the fossil mammal collection), Brussels, Belgium
IZUAN: I.I. Schmalhausen Institute of Zoology of the Academy of Sciences of Ukraine, Kiev, Ukraine
MAB: Oertijdmuseum de Groene Poort, Boxtel, the Netherlands
MNHN: Muséum National d’Histoire Naturelle, Paris, France (“F,” fossil collection, “SAS,” South Sacaco)
MSC: National Museum Support Center, Smithsonian Institution, Suitland, Maryland, USA
USNM: Department of Paleobiology, National Museum of Natural History, Smithsonian Institution, Washington, DC, USA.

## Additional Information and Declarations

### Data Availability

The following information was supplied regarding data availability: The raw data has been supplied as Supplemental Dataset Files.

### New Species Registration

The following information was supplied regarding the registration of a newly described species: Publication LSID: urn:lsid:zoobank.org:pub:1310A48E-A725-40E7-AFFB-D0A9043CFE04 Nanophoca gen. nov. urn:lsid:zoobank.org:act:251A3A76-F85D-4905-8BF0-48BB97EE8A4D Nanophoca vitulinoides (Van Beneden, 1877) urn:lsid:zoobank.org:act:CED9E2DD-DF9E-41BD-94C6-33C5C3B6F278
