# Supplementary material for: Reappraisal of the extinct seal “Phoca” vitulinoides from the Neogene of the North Sea Basin, with bearing on its geological age, phylogenetic affinities, and locomotion
Source: PeerJ. 2017 May 16;5:e3316. doi: 10.7717/peerj.3316 (PMC5436577; doi:10.7717/peerj.3316)
Supplement: Supplemental Information 2 [file peerj-05-3316-s002.docx]

**Supplemental Information**

List of supplemental tables and figures:

**1. Comparative material**

Supplemental List 1: Extant taxa.

Supplemental List 2: Fossil taxa.

**2. Measurements**

Supplemental Table 1: Measurements of vertebrae.

Supplemental Table 2: Measurements of scapula.

Supplemental Table 3: Measurements of humerus.

Supplemental Table 4: Measurements of ulna.

Supplemental Table 5: Measurements of radius.

Supplemental Table 6: Measurements of femur.

Supplemental Table 7: Measurements of tibia.

Supplemental Table 8: Measurement of calcaneum.

**3. Dinoflagellate cyst biostratigraphy**

Supplemental Table 9: List of dinoflagellate cyst species and acritarchs.

**4. Long bone variation contemporaneous small phocines**

Supplemental Table 10: Measurements of humerus characters.

Supplemental Table 11: Measurements of humerus characters.

**5. Interspecific variation for phocine innominates**

Supplemental Figure 1: Illustration of the angle quantifying lateral eversion of the ilium.

Supplemental Table 12: Angle quantifying lateral eversion of the ilium among Phocidae.

Supplemental Figure 2: Box-plot of the measurements of Supplemental Table 12.

**6. Phylogeny**

Supplemental List 3: Character list for the phylogenetic analysis.

Supplemental Figure 3: Node numbers on the 50% bootstrap consensus tree for the apomorphy list of Supplemental Table 13.

Supplemental Table 13: List of apomorphies and character changes between nodes within the 50% bootstrap consensus tree.

**7. Body length estimation**

Supplemental Table 14: Long bone lengths of *Phoca vitulina* and *Pusa sibirica* to estimate total body length.

**8. References**

**1. Comparative material**

**Supplemental List 1:** Extant species and specimens used as comparative material for the re-investigation of *Nanophoca vitulinoides*.

***Comparative Material (extant taxa)—****Cystophora cristata* (Erxleben, 1777) (USNM 118962, USNM 550411), *Erignathus barbatus* Erxleben, 1777 (USNM 230952, USNM 300704; USNM 500250, USNM 500251), *Halichoerus grypus* (Fabricius, 1791) (IRSNB 12550, IRSNB 34548, USNM 53291), *Histriophoca fasciata* (Zimmermann, 1783) (USNM 399449, USNM 504959, USNM 504960, USNM 571367), *Hydrurga leptonyx* (Blainville, 1820) (IRSNB 15388), *Leptonychotes weddellii* (Lesson, 1826) (IRSNB 15390), *Lobodon carcinophaga* (Hombron & Jacquinot, 1842) (IRSNB 13307), *Monachus monachus* Hermann, 1779 (IRSNB 1153), *Ommatophoca rossi* (Gray, 1844) (IRSNB 1164), *Pagophilus groenlandicus* (Erxleben, 1777) (IRSNB 1555D), *Phoca vitulina* (IRSNB 39043), *Pusa hispida* (USNM 225778, USNM 341617), *Pusa caspica* (Gmelin, 1788) (USNM 341615, USNM 341616), *Pusa sibirica* (Gmelin, 1788) (IRSNB, 15264, IRSNB 21171).

**Supplemental List 2:** Extinct species and specimens used as comparative material for the re-investigation of *Nanophoca vitulinoides*.

***Comparison Material (extinct taxa)—***Not considering the “*Phoca*” (*Nanophoca*) *vitulinoides* specimens, which are listed in the ‘referred specimens’ section below and Supplemental Information. *Acrophoca longirostris* (holotype MNHN.F.SAS 563), *Batavipusa neerlandica* Koretsky & Peters, 2008 (MAB 3798, MAB 04342), *Cryptophoca maeotica* (Nordmann, 1860) (USNM 489174 cast, USNM 489179 cast), *Leptophoca proxima* (lectotype IRSNB 1146-M279, IRSNB 1145-M280a-b, USNM 5359, USNM 5361, USNM 23224, USNM 23243, USNM 23450, USNM 175578, USNM 186990, USNM 205499, USNM 263648, USNM 284721, USNM 305247 cast, USNM 321934, USNM 411889, USNM 412115, USNM 454770), *Monachopsis pontica* (Eichwald, 1850) (USNM 1802 cast, USNM 214967 cast), *Piscophoca* *pacifica* (holotype MNHN.F.SAS 564, MNHN.F.SAS 488, MNHN.F.SAS 682). *Praepusa vindobonensis* (USNM cast of humerus without number, cast USNM 214964, cast USNM 214993; original specimens figured in Koretsky, 2001), *Prophoca rousseaui* Van Beneden, 1877 (lectotype IRSNB 1147-M275, IRSNB 1149-M274, IRSNB1150-M277a-b, IRSNB 1192-M276a-d IRSNB M2234, IRSNB-VERT-3250-15), *Sarmatonectes sintsovi* Koretsky, 2001 (cast USNM 1713/146, USNM cast of femur without number).

**3. Measurements**

Measurements of selected long bones and of other skeletal remains of *Nanophoca vitulinoides* have been taken with a mechanical caliper, with a resolution of 0.1 mm. Measurements of *Praepusa boeska* and *Praepusa vindobonensis* have been adopted from Koretsky (2001) and Koretsky, Peters & Rahmat (2015). For reasons of consistency, measurements of the humerus and femur presented here follow the scheme used by Koretsky (2001) and a number of subsequent publications, e.g., Koretsky, Ray & Peters (2012), Koretsky & Rahmat (2013).

**Supplemental Table 1:** Measurements of vertebrae (in mm).

|  | IRSNB M2268 | IRSNB M2274 | IRSNB 1226 M244b | IRSNB M2269 | IRSNB M2276j | IRSNB M2276k | IRSNB M2273 | IRSNB M2276m | IRSNB M2276l | IRSNB 1059-M240F | IRSNB 1059-M240E | IRSNB 1059-M240D | IRSNB M2276n | IRSNB M2276o | IRSNB M2276p | IRSNB M2276q |
| --- | --- | --- | --- | --- | --- | --- | --- | --- | --- | --- | --- | --- | --- | --- | --- | --- |
| Absolute height | 32.2 | 20.9 | N/A | N/A | N/A | N/A | 31.2 | 30.8 | 34.5 | N/A | N/A | N/A | N/A | N/A | N/A | N/A |
| Width across transverse processes | >35.8 | c. 31.2 | 45.9 | 46.6 | 34.7 | 38.1 | 33.0 | 31.6 | N/A | N/A | N/A | N/A | N/A | N/A | N/A | N/A |
| Height body and medial ventral ridge | 13.0 | N/A | 13.4 | 13.6 | N/A | N/A | N/A | N/A | N/A | N/A | N/A | N/A | N/A | N/A | 18.0 | N/A |
| Height body | 10.8 | 10.0 | 10.9 | 10.9 | 12.6 | 12.8 | 14.0 | 13.5 | 14.0 | 16.3 | 16.7 | 18.1 | 14.9 | 16.2 | 17.0 | 20.2 |
| Width body | 14.9 | 13.8 | 14.7 | 14.5 | 14.9 | 15.1 | 18.5 | 15.6 | 18.1 | 17.6 | 20.1 | 22.0 | 19.6 | 19.2 | 19.0 | 20.0 |
| Length body | 33.0 | 15.4 | 12.0 | 13.2 | 15.3 | 13.3 | 17.5 | 16.2 | 17.3 | 21.6 | 21.4 | 20.8 | 17.9 | 19.0 | 19.3 | 29.8 |

**Supplemental Table 2:** Measurements of scapula (in mm).

|  | IRSNB M2276f | IRSNB 1068-M241 |
| --- | --- | --- |
| Mediolateral width glenoid | 13.2 | 13.5 |
| Dorsoventral width glenoid | 20.9 | 22.1 |
| Mediolateral width glenoid neck | 8.9 | 8.7 |
| Dorsoventral width glenoid neck | 14.8 | 16.4 |

**Supplemental Table 3:** Measurements of humerus (in mm).

|  | *Nanophoca vitulinoides* | | *Praepusa boeska* | *Praepusa vindobonensis* |
| --- | --- | --- | --- | --- |
|  | IRSNB M2276c | IRSNB 1063-M242 | MAB 4686 | Average values from Koretsky (2001) |
| Total length | 72.4 | 78.2 | 81.1 | 86.3 |
| Length deltopectoral crest | 38.0 | 40.7 | 50.4 | 57.0 |
| Height head | 16.5 | 17.8 | 18.6 | 17.0 |
| Height trochlea | 11.1 | 12.6 | 13.2 | 13.9 |
| Width head | 19.3 | 19.6 | 19.4 | 18.4 |
| Width deltopectoral crest | 17.5 | 16.1 | 13.5 | 18.6 |
| Width proximal epiphysis | 24.0 | 26.6 | 21.2 | 25.6 |
| Width distal epiphysis | 27.5 | 28.1 | 23.5 | 27.6 |
| Distal width trochlea | 17.5 | 19.7 | 18.2 | 15.7 |
| Anterior width trochlea | 12.5 | 12.3 | 16.7 | 13.1 |
| Transverse width mid-diaphysis | 9.8 | 9.5 | 11.3 | 10.6 |
| Anteroposterior thickness proximal epiphysis | 29.4 | 34.0 | 22.9 | 29.0 |
| Anteroposterior thickness medial condyle | 12.1 | 13.4 | 16.9 | 14.0 |
| Anteroposterior thickness lateral condyle | 12.3 | 14.2 | 13.9 | 15.2 |
| Diameter mid-diaphysis and deltopectoral crest in lateral view | 20.0 | 20.8 | 21.1 | 24.2 |

**Supplemental Table 4:** Measurements of ulna (in mm).

|  | IRSNB M2272 |
| --- | --- |
| Absolute length | 95.0 |
| Height trochlear notch | 19.4 |
| Anteroposterior width mid-diaphysis | 9.6 |
| Mediolateral width mid-diaphysis | 6.5 |

**Supplemental Table 5:** Measurements of radius (in mm).

|  | IRSNB M2278 |
| --- | --- |
| Absolute length | 77.1 |
| Mediolateral width proximal epiphysis | 10.3 |
| Anteroposterior width proximal epiphysis | 12.7 |
| Mediolateral width bicipital tuberosity | 7.5 |
| Mediolateral width below bicipital tuberosity | 5.0 |
| Anteroposterior width below bicipital tuberosity | 9.2 |
| Mediolateral width distal epiphysis | 12.1 |
| Anteroposterior width distal epiphysis | 21.5 |

**Supplemental Table 6:** Measurements of femur (in mm).

|  | *Nanophoca vitulinoides* | | | *Praepusa vindobonensis* |
| --- | --- | --- | --- | --- |
|  | IRSNB M2276d | IRSNB M2271 | IRSNB 1049-M247 | Average values from Koretsky (2001) |
| Total length | 69.4 | 71.5 | 73.6 | 72.8 |
| Medial length: proximodistal length proximal margin of head to distal margin of medial condyle | 61.5 | 62.3 | 62.5 | 69.2 |
| Lateral length: proximodistal length proximal margin greater trochanter to distal margin of lateral condyle | 62.6 | 64.3 | 65.7 | 67.3 |
| Proximodistal length medial condyle | 12.3 | 11.6 | 11.7 | 12.9 |
| Proximodistal length lateral condyle | 15.0 | 14.5 | N/A | 14.7 |
| Length greater trochanter | 21.8 | 19.7 | 20.8 | 20.3 |
| Intertrochanteric length | 24.9 | 25.9 | 28.2 | 26.7 |
| Height head | N/A | 14.2 | 15.5 | 13.3 |
| Height patella | 7.3 | 9.7 | 10.8 | 15.2 |
| Width proximal epiphysis | 33.5 | 33.6 | 34.6 | 36.5 |
| Width distal epiphysis | 33.7 | 35.1 | 36.8 | 37.5 |
| Width head | 12.7 | 13.8 | 14.9 | 14.6 |
| Width diaphysis | 19.6 | 20.3 | 19.8 | 18.4 |
| Thickness diaphysis | 9.1 | 9.5 | 9.7 | 10.4 |
| Thickness medial condyle | 17.5 | 17.0 | 19.8 | 18.7 |
| Thickness lateral condyle | 17.5 | 18.4 | N/A | 20.4 |
| Intercondylar distance | 7.2 | 7.0 | 8.5 | 11.6 |
| Diameter neck | 10.6 | 11.7 | 13.1 | 11.0 |

**Supplemental Table 7:** Measurements of tibia (in mm).

|  | IRSNB M2276g | IRSNB 1105-M239 |
| --- | --- | --- |
| Absolute length | N/A | 151.9 |
| Mediolateral width tibial plateau | 29.6 | 30.0 |
| Anteroposterior breadth tibial plateau | 19.4 | 18.1 |
| Mediolateral width mid-diaphysis | 12.4 | 12.4 |
| Anteroposterior length mid-diaphysis | 9.8 | 9.3 |

**Supplemental Table 8:** Measurement of calcaneum (in mm).

|  | IRSNB M2275 |
| --- | --- |
| Absolute proximodistal length | 34.8 |

**2. Dinoflagellate cyst biostratigraphy**

**Supplemental Table 9:** Qualitative list of the dinoflagellate cyst species and acritarchs recorded in the samples 1018/1019 and 1026 during systematic scanning. ‘X’ denotes presence in the sample.

| **Dinoflagellate cysts** | 1018/1019 | 1026 |
| --- | --- | --- |
| *Ataxodinium zevenboomii* Head 1997 | x |  |
| *Barssidinium pliocenicum* (Head 1993) | x |  |
| *Batiacasphaera deheinzelinii* Louwye 1999 | x |  |
| *Baticasphaera minuta* (Matsuoka 1983) | x | x |
| *Cleistosphaeridium placacanthum* (Deflandre and Cookson 1955) | x | x |
| *Cordosphaeridium minimum* (Morgenroth 1966) | x |  |
| *Cordosphaeridium* spp. indet. | x |  |
| *Dapsilidinium pastielsii* (Davey and Williams 1966) | x |  |
| *Filisphaera microoronata* (Head et al. 1989) | x |  |
| *Habibacysta tectata* Head et al. 1989 | x | x |
| *Hystrichokolpoma rigaudiae* Deflandre and Cookson 1955 | x | x |
| *Labyrinthodinium truncatum* Piasecki 1980 | x |  |
| *Lingulodinium machaerophorum* (Deflandre and Cookson 1955) | x | x |
| *Operculodinium*? *borgerholtense* Louwye 2001 | x |  |
| *Operculodinium*? *eirikianum* Head et al. 1989 | x |  |
| *Operculodinium israelianum* (Rossignol 1962) | x | x |
| *Operculodinium piaseckii* Strauss and Lund 1992 | x |  |
| *Operculodinium tegillatum* Head 1997 | x |  |
| *Paleocystodinium golzowense* Albert 1961 |  | x |
| *Polyshaeridium zoharyii* (Rosignol 1962) | x |  |
| *Reticulatosphaera actinocoronata* (Benedek 1972) | x | x |
| *Selenopemphix brevispinosa* Head et al. 1989 |  | x |
| *Spiniferites* spp. indet. | x | x |
| **Acritarchs** |  |  |
| *Paralecaniella indentata* Cookson and Eisenack 1970 |  | x |
| *Porcupinea collaris* Quaijtaal et al. 2015 | x |  |

**4. Long bone variation in contemporaneous small phocines**

**Supplemental Table 10:** Dataset of the selected character measurements used to quantitatively distinguish the humerus of *Nanophoca vitulinoides* from that of other contemporanous small phocine seals from the North Sea basin (*Batavipusa neerlandica* and *Praepusa boeska*) and the Paratethys (*Praepusa vindobonenesis*). Measurements in mm. Corresponding scatterplot in the main text: Figure 14.

| **Character** | ***Batavipusa neerlandica* (MAB 3798) (holotype)** | ***Nanophoca vitulinoides* (IRSNB M2276c) (neotype)** | ***Nanophoca vitulinoides* (IRSNB 1063-M242)** | ***Praepusa boeska***  **(MAB 4686) (holotype)** | ***Praepusa vindobonensis* (USNM unnumbered cast)** | ***Praepusa vindobonensis* (USNM unnumbered cast)** | ***Praepusa vindobonensis* (USNM 489187 cast)** | ***Praepusa vindobonensis average* (n) (Koretsky, 2001)** |
| --- | --- | --- | --- | --- | --- | --- | --- | --- |
| **Total length (1)** | 64.9 | 72.4 | 78.2 | 81.1 | 84.0 | 81.1 | 90.4 | 86.3 (n=19) |
| **Length deltopectoral crest (2)** | 36.7 | 38.0 | 40.7 | 50.4 | 52.7 | 56.6 | 68.9 | 57.0 (n=19) |
| **Mediolateral width proximal epiphysis (3)** | 23.3 | 27.5 | 28.1 | 23.5 | 24.4 | 29.1 | 35.6 | 27.6 (n=34) |
| **Mediolateral width diaphysis (4)** | 11.8 | 9.8 | 9.5 | 11.3 | 9.6 | 12.1 | 12.5 | 10.6 (n=19) |
|  |  |  |  |  |  |  |  |  |
| **(1) : (2)** | 1.77 | 1,91 | 1.92 | 1.61 | 1.59 | 1.43 | 1.31 | 1.51 |
| **(3) : (4)** | 1.97 | 2.81 | 2.96 | 2.08 | 2.54 | 2.40 | 2.85 | 2.60 |

**Supplemental Table 11:** Dataset of the selected character measurements used to quantitatively distinguish the femur of *Nanophoca vitulinoides* from that of *Praepusa vindobonenesis*. Measurements in mm. Corresponding scatterplot in the main text: Figure 16.

| **Character** | ***Nanophoca vitulinoides* (IRSNB M2276d) (neotype)** | ***Nanophoca vitulinoides* (IRSNB M2271)** | ***Nanophoca vitulinoides***  **(IRSNB 1049-M247)** | ***Praepusa vindobonensis average* (n) (Koretsky, 2001)** |
| --- | --- | --- | --- | --- |
| **Total length (1)** | 69.4 | 71.5 | 73.6 | 72.8 (n=22) |
| **Width distal epiphysis (2)** | 33.7 | 35.1 | 36.8 | 37.5 (n=30) |
| **Width diaphysis (3)** | 19.6 | 20.3 | 19.8 | 18.4 (n=34) |
| **Thickness diaphysis (4)** | 9.1 | 9.5 | 9.7 | 10.4 (n=31) |
|  |  |  |  |  |
| **(1) : (2)** | 2.15 | 2.14 | 2.04 | 1.77 |
| **(3) : (4)** | 2.06 | 2.04 | 2.00 | 1.94 |

**5. Interspecific variation for phocine innominates**

The sample of investigated innominates includes multiple genera of both the Monachinae and the Phocinae. The sample is limited to the material present at the IRSNB, only including skeletally adult and subadult specimens. Because most genera/species are only represented by one specimen, and because the pelvis is one of the regions that shows high sexual intraspecific variation (i.e., females need to give birth and require a wider pelvis), this study shows only preliminary data. To measure the angle of the lateral eversion of the innominate, two lines are drawn on images of the pelvis in dorsal view (Supplemental Fig. 3). Line one from the lateral tip of the anterodorsal process of the ilium to the point where the contact between the anterior margin of the contact of the posterodorsal process with the sacral wing forms an angle, and line two from the latter point to the ischiatic spine.


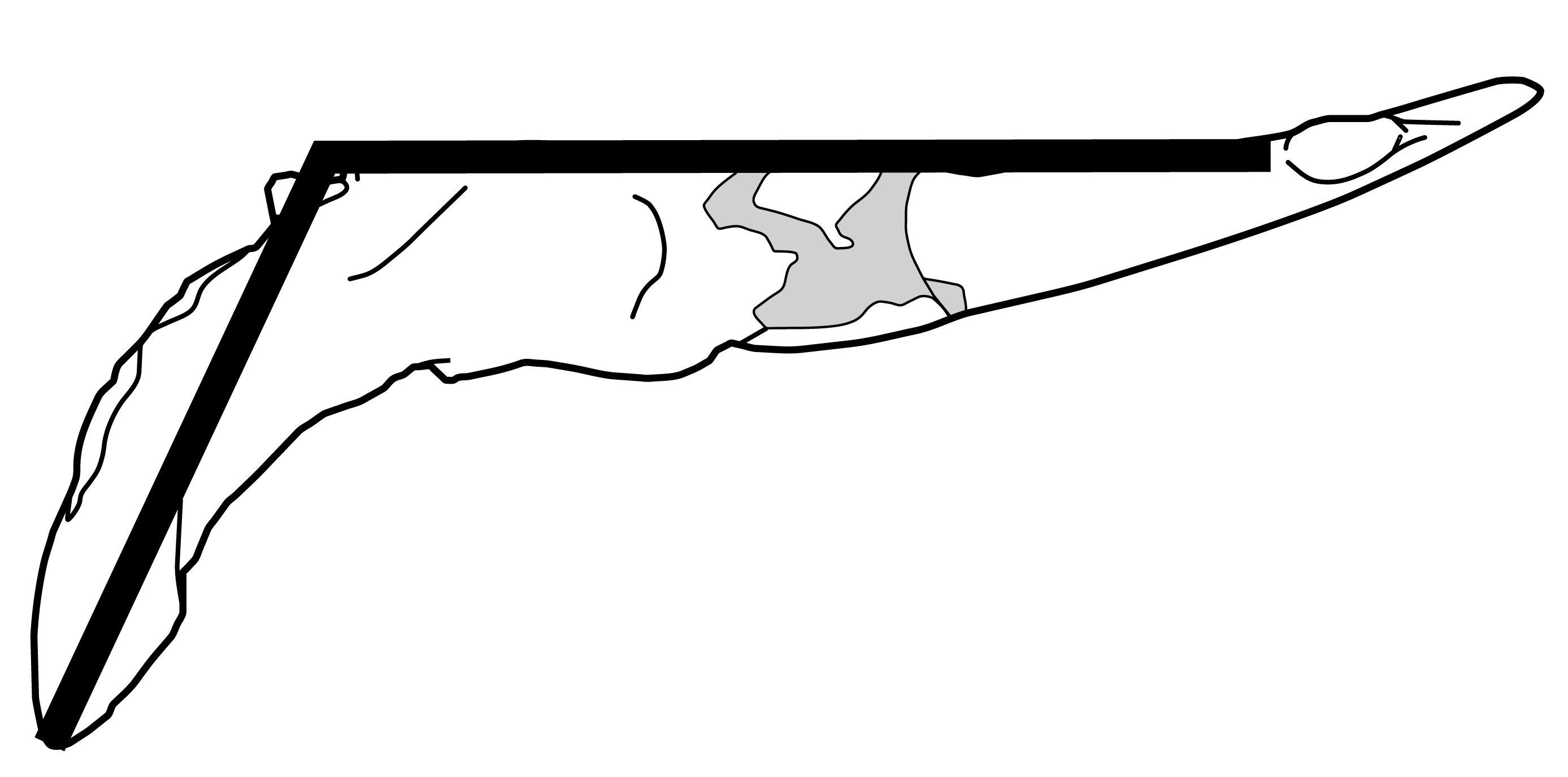


**Supplemental Figure 1:** Illustration of the angle quantifying lateral eversion of the ilium in dorsal view. Measured over the acute angle.

**Supplemental Table 12:** Angle quantifying lateral eversion of the ilium among Phocidae, including 13 extant specimens and one fossil specimen (indicated by ‘†’).

| **Specimen** | **Taxon** | **Angle eversion (°)** |
| --- | --- | --- |
| IRSNB 12550 | *Halichoerus grypus* | 79.4 |
| IRSNB 15388 | *Hydrurga leptonyx* | 66.2 |
| IRSNB 15390 | *Leptonychotes weddelli* | 74.8 |
| IRSNB 1161 | *Lobodon carcinophaga* | 61.3 |
| IRSNB 16039 | *Mirounga leonina* | 69.1 |
| IRSNB 1153 | *Monachus monachus* | 53.6 |
| IRSNB 15389 | *Ommatophoca rossi* | 68.8 |
| IRSNB 1155 | *Pagophilus groenlandicus* | 74.2 |
| IRSNB 7605 | *Phoca vitulina* | 72.3 |
| IRSNB 13098 | *Phoca vitulina* | 76.2 |
| IRSNB 17592 | *Phoca vitulina* | 63.8 |
| IRSNB 15532 | *Pusa sibirica* | 78.1 |
| IRSNB 21171 | *Pusa sibirica* | 78.3 |
| IRSNB 1059-M240 | *Nanophoca vitulinoides* † | 77.2 |

Average Phocinae (seven measurements, excluding *N. vitulinoides*): 74.6°


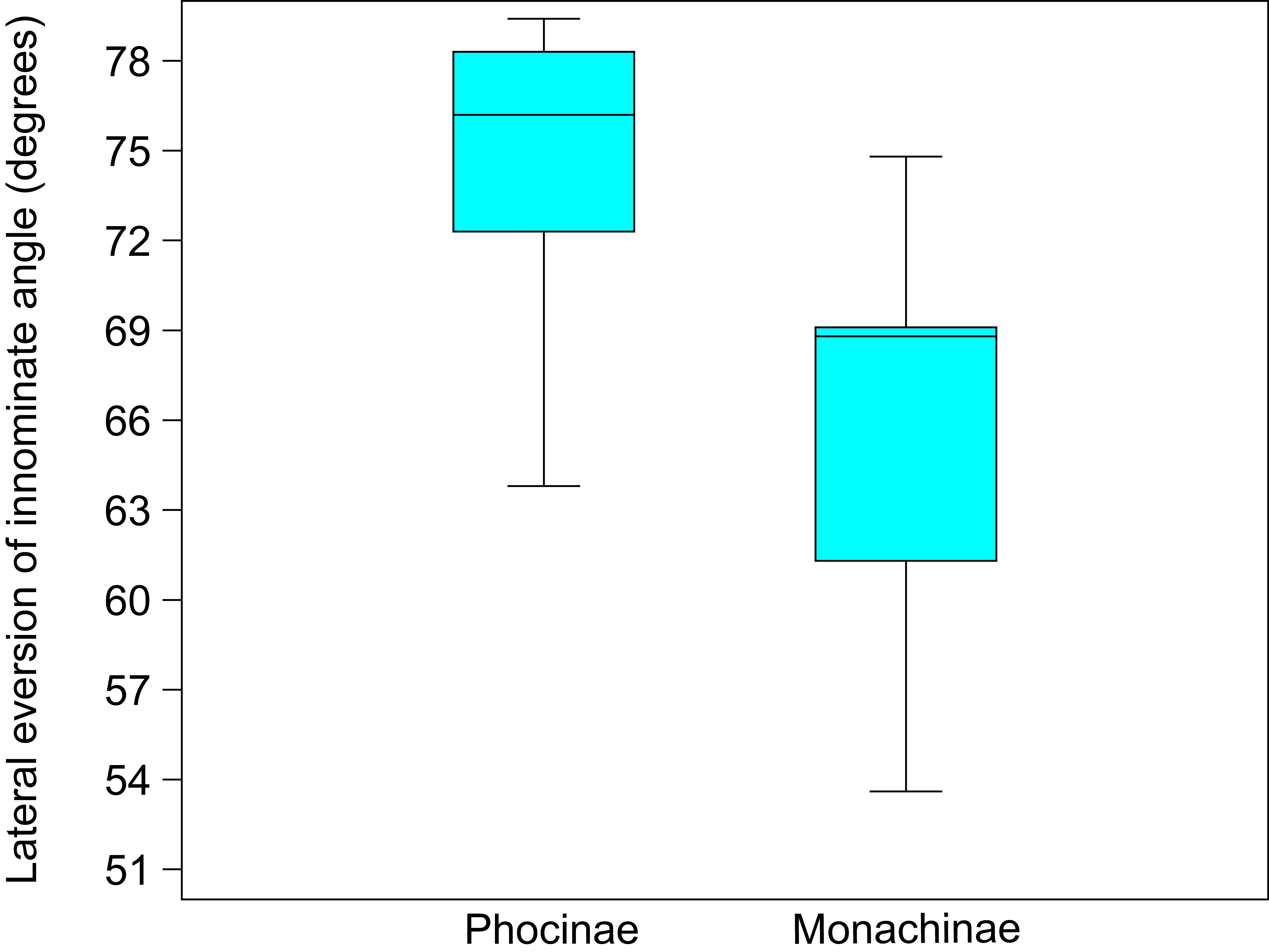
Average Monachinae: 65.6°

**Supplemental Figure 2:** Box-plot of the measurements of Supplemental Table 12, showing that the lateral eversion of the iliac wings in Phocinae is on average significantly larger than in Monachinae.

**6. Phylogeny**

**Supplemental List 3:** Character list for the phylogenetic analysis, including references to selected recent publications from which the characters have been taken, or adapted. Note that these references refer either to characters presented for the first time by these authors, or to characters adopted or adapted from other sources: see references therein.

**Cranial, mandibular, and dental characters (unordered unless stated otherwise).**

1. Premaxilla-nasal suture: (0) extensive; (1) weakly reduced; (2) strongly reduced. (Berta & Wyss, 1994; Bininda-Emonds & Russell, 1996; Cozzuol, 2001; Amson & Muizon, 2013; Berta et al., 2015)
2. Premaxilla-maxilla suture: 0) entirely lateral to nasal cavity; 1) anterior portion partially included in nasal cavity; (2) entirely within nasal cavity. (Berta & Wyss, 1994; Bininda-Emonds & Russell, 1996; Cozzuol, 2001; Amson & Muizon, 2013; Berta et al., 2015) Adjusted from other studies because the additional condition (2) has been observed in *Cystophora cristata* and *Mirounga*.
3. Nasals, shape of posterior edge: (0) pointed; (1) rounded or square; (2) frontals insert between nasals. (Berta & Wyss, 1994; Bininda-Emonds & Russell, 1996; Koretsky, 2001; Amson & Muizon, 2013)
4. Facial angle: (0) snout more anteriorly than dorsally (angle 45° or more); (1) snout opening more dorsally than anteriorly (angle 45° or less). (Bininda-Emonds & Russell, 1996; Amson & Muizon, 2013)
5. Lateral border of opening of nasal cavity in lateral view: (0) rectilinear or weakly concave; (1) strongly concave. (Amson & Muizon, 2013)
6. Position of posterior end of nasals: (0) anterior to maxilla-frontal suture; (1) posterior to maxilla-frontal suture but greatly anterior to the level of the jugal-squamosal suture; (2) almost reaches the level of the jugal-squamosal suture. (Berta & Wyss, Amson & Muizon, 2013)
7. Maxilla, swelling: (0) not present; (1) present. (Bininda-Emonds & Russell, 1996; Koretsky, 2001; Koretsky & Grigorescu, 2002; Koretsky & Rahmat, 2013)
8. Alveolar process of maxilla: (0) facing ventrally; (1) facing anteroventrally posterior to P1. (Amson & Muizon, 2013)
9. Maxillary process of jugal (at level of the anteroventral border of the orbit) in lateral view: (0) thin and low and increasing progressively posteriorly; (1) thick and high and increasing abruptly posteriorly. (Amson & Muizon, 2013)
10. Position of anterior opening of infraorbital foramen in ventral view: (0) anterior to M1; (1) level or posterior to M1. (Amson & Muizon, 2013)
11. Jugal, anterior end in dorsal view: (0) lateral to infraorbital foramen; (1) above or medial to the lateral margin of the infraorbital foramen. (Amson & Muizon, 2013)
12. Jugal, direction of arch of anterior portion: (0) downwards; (1) flat, no distinct arch; (2) upwards. (Bininda-Emonds & Russell, 1996; Amson & Muizon, 2013)
13. Squamosal-jugal articulation: (0) splintlike; (1) mortised. (Berta & Wyss, 1994; Bininda-Emonds & Russell, 1996; Amson & Muizon, 2013)
14. Ventral edge of the zygomatic arch, in anterior view: (0) higher than alveolar plane; (1) level with the alveolar plane (or very close to). (Bininda-Emonds & Russell, 1996; Amson & Muizon, 2013)
15. Supraorbital process of frontal: (0) absent or weakly developed; (1) strongly developed. (Berta & Wyss, 1994; Bininda-Emonds & Russell, 1996; Amson & Muizon, 2013)
16. Orbital vacuities: (0) absent; (1) present. (Berta & Wyss, 1994; Bininda-Emonds & Russell, 1996)
17. Interorbital, least width: 0) in posteriormost portion of interorbital septum; 1) in anterior half of the interorbital septum. (Berta & Wyss, 1994; Bininda-Emonds & Russell, 1996; Cozzuol, 2001; Amson & Muizon, 2013)
18. Interorbital least width:braincase width ratio: (0) high (i.e., much more than 20% of width of skull); (1) moderate (i.e., approximately 20%); (2) low (i.e., much less than 20%); (3) very low (i.e., 5% or less). (Koretsky, 2001; Koretsky & Grigorescu, 2002; Koretsky & Rahmat, 2013; Amson & Muizon, 2013)
19. Major axes of glenoid fossae: (0) sub-parallel; (1) slightly convergent posteriorly. (Amson & Muizon, 2013)
20. Orientation of medial margins of tympanic bullae: (0) diverging posteriorly; (1) parasagittal. (Amson & Muizon, 2013)
21. Lateral end of tympanic bulla: (0) medial to level of mid-width of glenoid fossa; (1) lateral to level of mid-width of glenoid fossa.Pterygoid process: (0) rounded with convex lateral margin; (1) flat with concave lateral margin. (Amson & Muizon, 2013)
22. Inflation of tympanic bulla: (0) weak; (1) moderate; (2) strong. (Berta & Wyss, 1994; Bininda-Emonds & Russell, 1996; Koretsky, 2001; Koretsky & Rahmat, 2013; Amson & Muizon, 2013)
23. Carotid canal, posterior opening: (0) visible in ventral view (i.e., at least partially facing ventrally); (1) not visible in ventral view (i.e., opening having very little ventral aspect). (Berta & Wyss, 1994; Bininda-Emonds & Russell, 1996; Cozzuol, 2001; Amson & Muizon, 2013)
24. Posterior opening of the carotid canal and posterior lacerate foramen: (0) clearly separated; (1) coalescent. (Amson & Muizon, 2013)
25. Mastoid: 0) not visible in dorsal view; 1) visible. (Berta and Wyss 1994; Bininda-Emonds & Russell, 1996; Cozzuol, 2001; Amson & Muizon, 2013)
26. Heavily pachyosteosclerotic mastoid: (0) absent; (1) present (Berta & Wyss, 1994; Bininda-Emonds & Russell, 1996; Amson & Muizon, 2013)
27. Relation of paroccipital process to mastoid: (0) connected by a low and discontinuous ridge; (1) connected by a high and continuous ridge; (2) well separated. (Berta & Wyss, 1994; Bininda-Emonds & Russell, 1996; Koretsky, 2001; Koretsky & Rahmat, 2013)
28. Pterygoid process: (0) rounded with convex lateral margin; (1) flat with concave lateral margin. (Berta & Wyss, 1994; Amson & Muizon, 2013).
29. Alisphenoid canal: (0) present; (1) absent. (Berta & Wyss, 1994; Bininda-Emonds and Russell, 1996; Amson & Muizon, 2013)
30. Direction of occipital condyles in occipital view: (0) ventral; (1) diverging dorsally. (Amson & Muizon, 2013)
31. Tooth rows: (0) parallel; (1) diverging posteriorly (Bininda-Emonds & Russell, 1996; Amson & Muizon, 2013)
32. Upper incisors: (0) three; (1) two; (2) one (ordered). (Berta & Wyss, 1994; Bininda-Emonds & Russell, 1996; Cozzuol, 2001; Koretsky, 2001; Koretsky & Grigorscu, 2002; Koretsky & Rahmat, 2013; Amson & Muizon, 2013; Berta et al., 2015)
33. Lower incisors: (0) three; (1) two; (2) one (ordered). (Berta & Wyss, 1994; Bininda-Emonds & Russell, 1996; Koretsky, 2001; Koretsky & Grigorescu, 2002; Koretsky & Rahmat, 2013; Amson & Muizon, 2013; Berta et al., 2015)
34. Lateral incisor, relative size: (0) incisiform; (1) intermediate shape; (2) caniniform. (Berta & Wyss, 1994; Bininda-Emonds & Russell, 1996; Koretsky, 2001; Koretsky & Grigorescu, 2002; Koretsky & Rahmat, 2013; Berta et al., 2015)
35. Upper incisor, roots: (0) strongly transversely compressed; (1) moderately transversely compressed. (Berta & Wyss, 1994; Bininda-Emonds & Russell, 1996; Cozzuol, 2001; Berta et al., 2015)
36. P2-4, p2-4, roots: (0) double-rooted; (1) single-rooted. (Berta & Wyss, 1994; Bininda-Emonds & Russell, 1996; Koretsky, 2001; Koretsky & Grigorescu, 2002; Koretsky & Rahmat, 2013; Amson & Muizon, 2013)
37. Postcanine teeth, crowns: (0) multi-cusped; (1) single-cusped. (Berta and Wyss, 1994; Bininda-Emonds & Russell, 1996; Koretsky, 2001; Koretsky & Grigorescu, 2002; Koretsky & Rahmat, 2013; Amson & Muizon, 2013; Berta et al., 2015)
38. Postcanine teeth, lingual cingulum: (0) well developed; (1) not or poorly developed. . (Berta and Wyss, 1994; Bininda-Emonds & Russell, 1996; Koretsky, 2001; Koretsky & Grigorescu, 2002; Koretsky & Rahmat, 2013; Amson & Muizon, 2013; Berta et al., 2015)
39. M1 and m1, roots: 0) double-rooted; 1) single-rooted. (Berta & Wyss, 1994; Bininda-Emonds & Russell, 1996; Amson & Muizon, 2013)
40. M2: (0) present; (1) absent. (Berta & Wyss, 1994; Bininda-Emonds & Russell, 1996; Amson & Muizon, 2013)
41. p4, size comparison m1: (0) about equal in size; (1) P4 and p4 larger than M1 and m1. (Berta & Wyss, 1994; Bininda-Emonds & Russell, 1996; Koretsky, 2001; Koretsky & Grigorescu, 2002; Koretsky & Rahmat, 2013; Berta et al., 2015)

**Postcranial characters (all are unordered).**

1. Atlas, transverse foramen: (0) visible in posterior view; (1) at least partially visible in dorsal view. (Berta & Wyss, 1994; Amson & Muizon, 2013)
2. Atlas, direction of transverse process in lateral view: (0) oblique; (1) sub-vertical. (Amson & Muizon, 2013)
3. Scapula: (0) two ridges on lateral side do not join near glenoid; (1) two ridges on lateral side join near glenoid (this study, see Hodgetts, 1999)
4. Humerus, lesser tubercle and head: (0) head higher or at same level as lesser tubercle; (1) tubercle higher. (Berta & Wyss, 1994; Koretsky, 2001; Koretsky & Grigorescu, 2002; Koretsky & Rahmat, 2013; Amson & Muizon, 2013; Berta et al., 2015)
5. Humerus, greater tubercle height: (0) below level of head; (1) at level of head or slightly above; (2) above level of head. (Berta & Wyss, 1994; Cozzuol, 2001; Koretsky, 2001; Koretsky & Grigorescu, 2002; Koretsky & Rahmat, 2013; Amson & Muizon, 2013; Berta et al., 2015)
6. Humerus, supinator: (0) strongly developed; (1) poorly developed. (Berta & Wyss, 1994; Bininda-Emonds & Russell, 1996; Cozzuol, 2001; Amson & Muizon, 2013; Berta et al., 2015).
7. Humerus, deltopectoral crest; (0) smooth distal termination; (1) sharp distal termination. (Berta & Wyss, 1994; Bininda-Emonds & Russell, 1996)
8. Humerus, deltoid crest proximal bifurcation: (0) present, either distinct or slight; (1) absent, crest has smooth edge. (this study, see Hodgetts, 1999)
9. Humerus, length of deltoid crest: (0) shorter than or subequal to one-half length of the bone, confined to the proximal half of the bone; (1) longer than one-half length of the bone. (Berta & Wyss, 1994; Bininda-Emonds & Russell, 1996; Koretsky, 2001; Koretsky & Grigorescu, 2002; Koretsky & Rahmat, 2013; Amson & Muizon, 2013; Berta et al., 2015)
10. Humerus, intertubercular groove: (0) narrow and deep; (1) intermediate state; (2) broad and shallow. (this study)
11. Humerus, transverse bar in bicipital groove: (0) absent; (1) present. (this study)
12. Humerus, entepicondylar foramen: (0) absent; (1) present. (Berta & Wyss, 1994; Bininda-Emonds & Russell, 1996; Cozzuol, 2001; Amson & Muizon, 2013; Berta et al., 2015)
13. Humerus, diameter of trochlea: (0) same as diameter of distal head; (1) considerably larger than distal head. (Berta & Wyss, 1994; Koretsky, 2001; Koretsky & Grigorescu, 2002; Koretsky & Rahmat, 2013)
14. Radius, location radial tuberosity: 0) medial side; 1) posteromedial side. (Amson & Muizon, 2013)
15. Radius, pronator teres process: 0) present, proximal; 1) present, distal. (Berta & Wyss, 1994)
16. Ulna, distal end of styloid process: 0) distally pointed; 1) flattened. (Amson & Muizon, 2013)
17. Metacarpal I, length: (0) slightly longer than mcII; (1) much longer. (Bininda-Emonds & Russell, 1996; Cozzuol, 2001; Berta et al., 2015)
18. Metapodials, head: (0) keeled with trochleated phalangeal articulations; (1) smooth, with phalanges flat, articulations hingelike. (Berta & Wyss, 1994; Cozzuol, 2001; Amson & Muizon, 2013; Berta et al., 2015)
19. Sacrum, number of fused vertebrae: (0) three; (1) four. (this study)
20. Innominate, anterodorsal iliac spine: (0) dorsal to anteroventral iliac spine; (1) posterodorsal to the anteroventral iliac spine. (Amson & Muizon, 2013)
21. Innominate, posteroventral iliac spine (=iliac tuberosity): (0) small or absent; (1) large and strongly protruding. (Amson & Muizon, 2013)
22. Innominate, iliopectineal eminence: (0) strongly developed; (1) moderately well developed; (2) small or absent. (this study)
23. Innominate, ilium: (0) shallow gluteal fossa; (1) deep gluteal fossa. (Bininda-Emonds & Russell, 1996; Cozzuol, 2001; Amson & Muizon, 2013)
24. Innominate, ilium: (0) weakly everted wing; (1) moderately everted; (2) strongly everted wing. (Bininda-Emonds & Russell, 1996; Cozzuol, 2001; Amson & Muizon, 2013)
25. Innominate, ilium: (0) long, compared to postacetabular region; (1) short. (Berta & Wyss, 1994; Bininda-Emonds & Russell, 1996; Cozzuol, 2001; Amson & Muizon, 2013)
26. Innominate, ischial spine: (0) unenlarged; (1) enlarged. (Berta & Wyss, 1994)
27. Femur, lesser trochanter: (0) present; (1) absent. (Berta and Wyss 1994; Bininda-Emonds & Russell, 1996; Cozzuol, 2001; Koretsky, 2001; Koretsky & Grigorescu, 2002; Koretsky & Rahmat, 2013)
28. Femur, neck, relative size: (0) thick; (1) narrow. (Koretsky, 2001; Koretsky & Grigorescu, 2002; Koretsky & Rahmat, 2013; Berta et al., 2015)
29. Femur, collo-diaphyseal angle: (0) high, head oriented more medially than proximally; (1) low, head oriented more proximally than medially. (Amson & Muizon, 2013)
30. Femur, distal condyles: (0) roughly similar in size or slight size difference; (1) large size difference. (Koretsky, 2001; Koretsky & Grigorescu, 2002; Koretsky & Rahmat, 2013; Berta et al., 2015)
31. Femur, epiphyses: (0) distal epiphysis wider than proximal; (1) widths of proximal and distal epiphyses about equal; (2) proximal epiphysis wider than distal one. (Koretsky, 2001; Koretsky & Grigorescu, 2002; Koretsky & Rahmat, 2013; Berta et al., 2015)
32. Femur, diaphysis: (0) minimum width less than or about equal to two-thirds width of proximal epiphysis; (1) minimum width more than two-thirds width of proximal epiphysis. (Koretsky, 2001; Koretsky & Grigorescu, 2002; Koretsky & Rahmat, 2013)
33. Femur, head and greater trochanter: (0) head reaches higher than greater trochanter; (1) both reach same level; (2) greater trochanter reaches higher than head. (Berta & Wyss, 1994; Bininda-Emonds & Russell, 1996; Cozzuol, 2001; Koretsky, 2001; Koretsky & Grigorescu, 2002; Koretsky & Rahmat, 2013; Berta et al., 2015)
34. Femur, trochanteric fossa: (0) little reduced; (1) strongly reduced or absent. (Berta & Wyss, 1994; Bininda-Emonds & Russell, 1996; Berta et al., 2015)
35. Femur, intertrochanteric crest: (0) strongly reduced; (1) relatively pronounced.
36. Femur, suprapatellar fossa: (0) absent; (1) present (this study).
37. Femur, orientation of fossa for *m. peroneus longus*: (0) lateral; (1) anterolateral. (Amson & Muizon, 2013)
38. Tibia and fibula: (0) proximal epiphyses not fused; (1) proximal epiphyses fused. (this study)
39. Tibia, development post-tibial fossa: (0) weak; (1) strong. (Berta & Wyss, 1994; Bininda-Emonds & Russell, 1996; Cozzuol, 2001; Amson & Muizon, 2013; Berta et al., 2015)
40. Astragalus, calcaneal process: (0) absent; (1) poorly developed; (2) well developed. (Berta & Wyss, 1994; Amson & Muizon, 2013)
41. Sustentacular facet of the astragalus: (0) oval-shaped and narrowed at contact with cuboid facet; (1) long (at least twice longer than wide), slender and strongly bent medially; (2) short and tongue-like with no narrowing at contact with cuboid facet. (Amson & Muizon, 2013; Berta et al., 2015)
42. Calcaneum, articular surface for fibula: (0) absent or very reduced; (1) well developed. (Amson & Muizon, 2013)
43. Metatarsal I, articular surface for metatarsal II: (0) oriented laterally; (1) oriented dorsolaterally; (2) inconspicuous. (Amson & Muizon, 2013)
44. Metatarsal III, length: (0) less than 50% shorter than metatarsal I; (1) approximately 50% shorter (or more) than metatarsal I. (Amson & Muizon, 2013)
45.
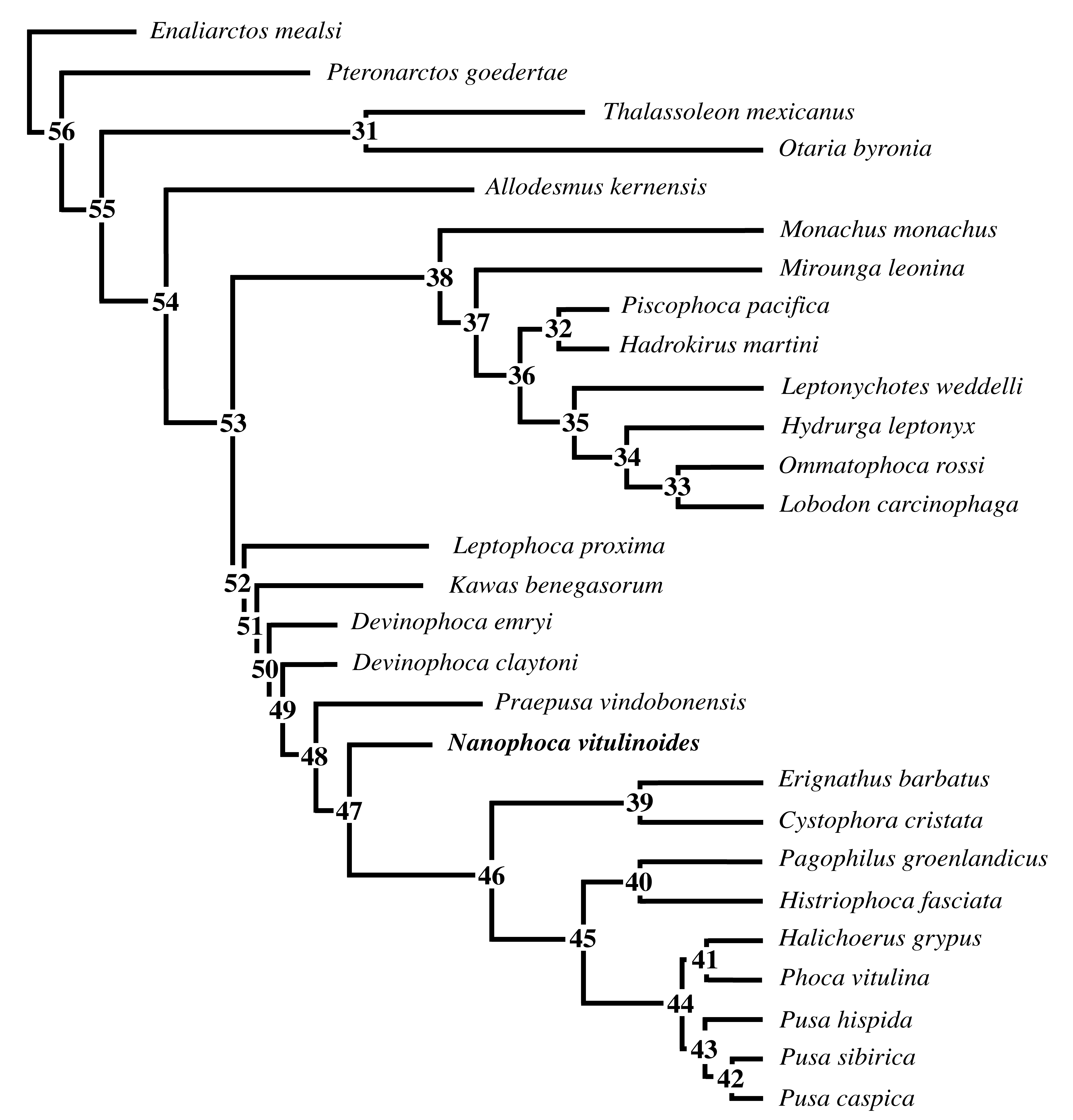


**Supplemental Figure 3:** Node numbers on the most parsimonous phylogenetic tree, excluding *Praepusa magyaricus* and *Praepusa pannonica* represent the node numbers as used to identify (syn)apomorphies in Supplemental Table 13.

**Supplemental Table 13:** List of apomorphies and character changes between nodes within the most parsimonous phylogenetic tree. The list of apomorphies results from the analysis using PAUP and as explained in the study. Ambiguous (syn)apomorphies are indicated by a simple arrow (-->), and unambiguous (syn)apomorphies by a double arrow (==>). Node numbers are specified on the tree above (Supplemental Figure 3).

| **Branch** | **Character number** | **Consistency Index** | **State change** |
| --- | --- | --- | --- |
| *Enaliarctos mealsi* <–> node 56 | 18  19  27  33  46  47  48  59  63  69  70  72  75 | 0.250  0.333  0.667  0.667  0.200  0.500  0.200  0.500  0.500  0.125  0.200  0.222  0.167 | 0 <=> 1  1 <=> 0  0 <=> 1  0 <–> 1  0 <–> 1  0 <–> 1  0 <–> 1  0 <–> 1  2 <–> 0  0 <–> 1  0 <–> 1  2 <–> 0  0 <–> 1 |
| Node 56 ––> *Pteronarctos goedertae* | 21  34  41 | 0.500  0.250  0.250 | 0 ==> 1  0 ==> 1  1 ==> 0 |
| Node 56 ––> node 55 | 3  4  9  16  31  36  37  49 | 0.500  0.167  0.250  1.000  0.500  0.333  0.143  0.200 | 1 ––> 0  0 ––> 1  1 ==> 0  0 ==> 1  1 ––> 0  0 ==> 1  0 ––> 1  1 ==> 0 |
| Node 55 ––> node 31 | 3  15  24  46  51  55  84 | 0.500  1.000  1.000  0.200  0.286  0.333  1.000 | 0 ––> 2  0 ==> 1  0 ––> 1  1 ==> 2  2 ==> 1  1 ==> 0  0 ––> 2 |
| Node 31 ––> *Thalassoleon mexicanus* | 11  37  41  48  50 | 0.200  0.143  0.250  0.333  0.333 | 0 ==> 1  1 ––> 0  1 ==> 0  0 ==> 1  1 ==> 0 |
| Node 31 ––> *Otaria byronia* | 4  6  12  34  36  39  56  72  79 | 0.167  0.667  0.400  0.250  0.333  0.200  0.500  0.286  0.500 | 1 ––> 0  1 ==> 0  0 ==> 2  0 ==> 2  1 ==> 2  0 ==> 1  0 ==> 1  0 ––> 1  0 ==> 1 |
| Node 55 --> node 54 | 1  2  10  13  18  27  28  38  53  68  71  81 | 0.400  0.400  0.500  1.000  0.333  0.667  1.000  0.250  0.500  0.500  0.500  1.000 | 0 ––> 1  0 ––> 1  0 ==> 1  0 ==> 1  1 ––> 2  1 ==> 2  0 ==> 1  1 ––> 0  0 ––> 1  0 ==> 1  0 ––> 1  0 ==> 1 |
| Node 54 ––> *Allodesmus kernensis* | 6  36  39  46  48  72  73 | 0.667  0.333  0.200  0.200  0.333  0.286  0.250 | 1 ==> 2  1 ––> 2  0 ==> 1  1 ––> 0  0 ==> 1  0 ––> 2  0 ==> 1 |
| Node 54 ––> node 53 | 1  5  21  22  26  29  30  31  34  40  63  66  67  70  79  81  82 | 0.400  0.500  0.500  1.000  1.000  1.000  0.500  0.500  0.250  1.000  0.500  1.000  0.500  0.200  0.500  1.000  0.500 | 1 ––> 2  0 ==> 1  0 ==> 1  0 ==> 1  0 ==> 1  0 ==> 1  0 ==> 1  0 ––> 1  0 ==> 1  0 ==> 1  0 ==> 1  0 ==> 1  0 ==> 1  1 ––> 0  0 ==> 1  1 ==> 2  0 ––> 1 |
| Node 53 ––> node 38 | 11  18  32  41  43  53  55  56  57  61  71  78  82  84  85 | 0.200  0.333  0.500  0.250  0.333  0.500  0.333  0.500  0.500  1.000  0.500  1.000  0.500  1.000  1.000 | 0 ==> 1  2 ––> 1  0 ==> 1  1 ==> 0  0 ==> 1  1 ––> 0  1 ==> 0  0 ==> 1  0 ==> 1  1 ==> 0  1 ––> 0  0 ==> 1  1 ––> 2  0 ==> 1  0 ==> 1 |
| Node 38 ––> node 37 | 42  58  69  76 | 0.333  0.500  0.167  0.250 | 0 ==> 1  0 ––> 1  1 ==> 0  1 ==> 0 |
| Node 37 ––> node 36 | 18  20  49  83 | 0.333  0.333  0.200  1.000 | 1 ––> 2  0 ==> 1  0 ––> 1  0 ==> 1 |
| Node 36 ––> node 32 | 8  9  34  51  52  58  63  75  82 | 0.500  0.250  0.250  0.286  0.333  0.500  0.500  0.167  0.500 | 0 ==> 1  0 ==> 1  1 ==> 0  2 ––> 1  0 ––> 1  1 ––> 0  1 ––> 0  1 ––> 0  2 ==> 1 |
| Node 32 ––> *Hadrokirus martini* | 1  12  43 | 0.400  0.400  0.333 | 2 ==> 1  0 ==> 1  1 ==> 0 |
| Node 36 ––> node 35 | 6  10  11  14  55 | 0.667  0.500  0.200  1.000  0.333 | 1 ==> 2  1 ==> 0  1 ==> 0  0 ==> 1  0 ==> 1 |
| Node 35 ––> node 34 | 18  37  38  45  49  70 | 0.333  0.143  0.250  0.500  0.200  0.200 | 2 ––> 0  1 ––> 0  0 ==> 1  0 ==> 1  1 ––> 0  0 ==> 1 |
| Node 34 ––> node 33 | 46  52  73 | 0.200  0.333  0.250 | 1 ==> 0  0 ==> 1  0 ==> 1 |
| Node 33 ––> *Lobodon carcinophaga* | 42  74  75 | 0.333  0.400  0.167 | 1 ==> 0  0 ==> 1  1 ==> 0 |
| Node 33 ––> *Ommatophoca rossi* | 2  7  8  25  37  51 | 0.400  0.500  0.500  0.500  0.143  0.286 | 1 ==> 2  0 ==> 1  0 ==> 1  0 ==> 1  0 ––> 1  2 ==> 0 |
| Node 34 ––> *Hydrurga leptonyx* | 18  50 | 0.333  0.333 | 0 ––> 1  1 ==> 0 |
| Node 37 ––> *Mirounga leonina* | 2  12  30  33  36  39  46  70 | 0.400  0.400  0.500  0.667  0.333  0.200  0.200  0.200 | 1 ==> 2  0 ==> 1  1 ==> 0  1 ==> 2  1 ==> 2  0 ==> 1  1 ==> 0  0 ==> 1 |
| Node 38 ––> *Monachus monachus* | 52 | 0.333 | 0 ==> 1 |
| Node 53 ––> node 52 | 2  12  20  38  47  51  54  59  60  62  74  75  77  80 | 0.400  0.400  0.333  0.250  0.500  0.286  0.500  0.500  1.000  0.500  0.400  0.167  0.500  1.000 | 1 ––> 0  0 ––> 1  0 ––> 1  0 ––> 1  1 ==> 0  2 ––> 0  0 ==> 1  1 ––> 0  0 ––> 1  0 ==> 1  0 ==> 1  1 ==> 0  0 ==> 1  0 ==> 1 |
| Node 52 ––> node 51 | 64  72 | 0.500  0.286 | 0 ==> 1  0 ––> 1 |
| Node 51 ––> node 50 | 65 | 1.000 | 0 ==> 1 |
| Node 50 ––> node 49 | 19  48 | 0.500  0.333 | 0 ==> 1  0 ––> 1 |
| Node 49 ––> *Devinophoca claytoni* | 1  3  4 | 0.400  0.500  0.167 | 2 ––> 1  0 ==> 1  1 ==> 0 |
| Node 49 ––> node 48 | 7  17  25  37 | 0.500  0.500  0.500  0.143 | 0 ==> 1  0 ==> 1  0 ==> 1  1 ==> 0 |
| Node 48 ––> node 47 | 20  22  23  50 | 0.333  1.000  1.000  0.333 | 1 ––> 0  1 ––> 2  0 ––> 1  1 ==> 0 |
| Node 47 ––> node 46 | 45  46  69  76  77 | 0.500  0.200  0.167  0.250  0.500 | 0 ==> 1  1 ––> 2  1 ––> 0  1 ––> 0  1 ==> 0 |
| Node 46 ––> node 39 | 3  18  34  51  64 | 0.500  0.333  0.250  0.286  0.500 | 0 ==> 1  2 ==> 1  1 ––> 0  0 ==> 1  1 ==> 0 |
| Node 39 ––> *Cystophora cristata* | 2  11  32  33  34  36  37  38  39  46  49  54  62  63  73 | 0.400  0.200  0.500  0.667  0.250  0.333  0.143  0.250  0.200  0.200  0.200  0.500  0.500  0.500  0.250 | 0 ==> 2  0 ==> 1  0 ==> 1  1 ==> 2  0 ––> 2  1 ==> 2  0 ==> 1  1 ==> 0  0 ==> 1  2 ––> 1  0 ==> 1  1 ==> 0  1 ==> 0  1 ==> 2  0 ==> 1 |
| Node 39 ––> *Erignathus barbatus* | 4  17  57  75  76 | 0.167  0.500  0.500  0.167  0.250 | 1 ==> 0  1 ==> 0  0 ==> 1  0 ==> 1  0 ––> 1 |
| Node 46 ––> node 45 | 12  35  65 | 0.400  1.000  1.000 | 1 ––> 2  1 ==> 0  1 ==> 2 |
| Node 45 ––> node 40 | 49  73  75 | 0.200  0.250  0.167 | 0 ==> 1  0 ==> 1  0 ==> 1 |
| Node 40 ––> *Histriophoca fasciata* | 4  41 | 0.167  0.250 | 1 ==> 0  1 ==> 0 |
| Node 40 –– > *Pagophilus groenlandicus* | 51  69 | 0.286  0.167 | 0 ==> 1  0 ––> 1 |
| Node 45 ––> node 44 | 44  46 | 0.500  0.200 | 0 ==> 1  2 ––> 0 |
| Node 44 ––> node 41 | 5  34 | 0.500  0.250 | 1 ==> 0  1 ––> 0 |
| Node 41 ––> *Halichoerus grypus* | 9  34  36  37  39 | 0.250  0.250  0.333  0.143  0.200 | 0 ==> 1  0 ––> 2  1 ==> 2  0 ==> 1  0 ==> 1 |
| Node 41 ––> *Phoca vitulina* | 4  11 | 0.167  0.200 | 1 ==> 0  0 ==> 1 |
| Node 44 ––> node 43 | 1  18  69  72 | 0.400  0.333  0.167  0.286 | 2 ==> 1  2 ==> 3  0 ––> 1  1 ==> 0 |
| Node 43 ––> node 42 | 46  74 | 0.200  0.400 | 0 ––> 1  1 ==> 2 |
| Node 47 ––> *Nanophoca vitulinoides* | 44  72  74 | 0.500  0.286  0.400 | 0 ==> 1  1 ––> 0  1 ==> 2 |
| Node 48 ––> *Praepusa vindobonensis* | 9  18  27  43  82 | 0.250  0.333  0.667  0.333  0.500 | 0 ==> 1  2 ==> 3  2 ==> 1  0 ==> 1  1 ––> 0 |
| Node 50 ––> *Devinophoca emryi* | 46  67  72 | 0.200  0.500  0.286 | 1 ==> 0  1 ==> 0  1 ––> 2 |
| Node 51 ––> *Kawas benegasorum* | 69  70  74  76 | 0.167  0.200  0.400  0.250 | 1 ==> 0  0 ==> 1  1 ==> 2  1 ==> 0 |
| Node 52 ––> *Leptophoca proxima* | 42  51  68 | 0.333  0.286  0.500 | 0 ==> 1  0 ––> 1  1 ==> 0 |

**7. Body length estimation**

**Supplemental Table 14:** Ratios of the humerus and femur of phocines *Phoca vitulina* and *Pusa sibirica*, in relation to the total body length. Humerus and femur lengths measured and ratios calculated against average body sizes retrieved from the literature: 150 cm for female *Ph. vitulina* (Storå, 2000) and 126.8 cm for female *Pu. sibirica* (Ciesielski et al., 2006).

|  | ***Phoca vitulina*** | | | | | ***Pusa sibirica*** |
| --- | --- | --- | --- | --- | --- | --- |
| **Specimen number** | **IRSNB 7605** | **IRSNB 1157C** | **IRSNB 36548** | **IRSNB 1165S** | **IRSNB 39043** | **MSC 175689** |
| **Humerus length (cm)** | 11.0 | 11.1 | 12.3 | 12.8 | 11.0 | 10.3 |
| **Femur length (cm)** | 10.6 | 9.9 | 10.9 | 11.3 | 9.7 | 9.8 |
| **Ratio humerus length to average body length (%)** | 7.33 | 7.40 | 8.20 | 8.53 | 7.33 | 8.12 |
| **Ratio femur length to average body length (%)** | 7.07 | 6.60 | 7.27 | 7.53 | 6.47 | 7.73 |

Average humerus ratio for *Phoca vitulina* (in %): 7.76 ± 0.57

Average femur ratio for *Phoca vitulina* (in %): 6.99 ± 0.45

**8. References**

Amson E, Muizon C de. 2013. A new durophagous phocid (Mammalia: Carnivora) from the late Neogene of Peru and considerations on monachine seal phylogeny. *Journal of Systematic Paleontology* 12:523-548.

Berta A, Kienle S, Bianucci G, Sorbi S. 2015. A Reevaluation of *Pliophoca etrusca* (Pinnipedia, Phocidae) from the Pliocene of Italy: Phylogenetic and Biogeographic Implications. *Journal of Vertebrate Paleontology* 35:e889144. DOI: 10.1080/02724634.2014.889144.

Berta A, Wyss AR. 1994. Pinniped phylogeny. *Proceedings of the San Diego Society of Natural History* 29:33-56.

Bininda-Emonds ORP, Russell AP. 1996. A morphological perspective on the phylogenetic relationships of the extant phocid seals (Mammalia: Carnivora: Phocidae). *Bonner Zoologische Monographien* 41:1-256.

Ciesielski T, Pastukhov MV, Fodor P, Bertenyi Z, Namiesnik J, Szefer P. 2006. Relationships and bioaccumulation of chemical elements in the Baikal seal (*Phoca sibirica*). *Environmental Pollution* 139:372-384.

Cozzuol MA. 2001. A ‘Northern” seal from the Miocene of Argentina: Implications for phocid phylogeny and biogeography. *Journal of Vertebrate Paleontology* 21:415-421. DOI: 10.1671/0272-4634(2001)021[0415:ANSFTM]2.0.C0;2.

Koretsky IA. 2001. Morphology and systematics of the Miocene Phocinae (Mammalia: Carnivora) from Paratethys and the North Atlantic Region. *Geologica Hungarica series Palaeontologica* 54:1–109.

Koretsky I. A., D. Grigorescu. 2002. The Fossil Monk Seal Pontophoca sarmatica (Alekseev) (Mammalia: Phocidae: Monachinae) from the Miocene of Eastern Europe; pp. 149–162 in R. J. Emry (ed.), Cenozoic Mammals of Land and Sea: Tributes to the Career of Clayton E. Ray. Smithsonian Contributions to Paleobiology 93.

Koretsky IA, Peters N, Rahmat S. 2015. New species of *Praepusa* (Carnivora, Phocidae, Phocinae) from the Netherlands supports east to west Neogene dispersal of true seals. *Vestnik Zoologii* 49:57-66. DOI: 10.1515/vzoo-2015-0006.

Koretsky, IA, Rahmat SJ. 2013. First record of fossil Cystophorinae (Carnivora, Phocidae): middle Miocene seals from the northern Paratethys. *Rivista Italiana di Paleontologia e Stratigrafia* 119:325-350.

Koretsky IA, Ray CE, Peters N. 2012. A new species of *Leptophoca* (Carnivora, Phocidae, Phocinae) from both sides of the North Atlantic Ocean (Miocene seals of the Netherlands, part I). *Deinsea* 15:1-12.

Storå J. 2000. Skeletal development in the Grey seal *Halichoerus grypus*, the Ringed seal *Phoca hispida botnica*, the Harbour seal *Phoca vitulina vitulina* and the Harp seal *Phoca groenlandica*. Epiphyseal fusion and life History. *Archaeozoologia* 11:199-222.
